# A robust framework for evaluating green mines towards sustainable development

**DOI:** 10.1038/s41598-025-21237-6

**Published:** 2025-10-29

**Authors:** Eman Sayed, Ahmed M. Ali, Ibrahim Alrashdi, Karam M. Sallam, Mohamed Abdel-Basset, Mahmoud M. Ismail

**Affiliations:** 1 Department of Decision Support, Faculty of Computers and Informatics, Zagazig University, Harayah Raznah, Zagazig, Sharqiyah 44519 Egypt; 2 Department of Computer Science, College of Computer and Information Sciences, Jouf University, Airport Road, Sakaka, Al-Jouf 2014 Saudi Arabia; 3 Department of Computer Science, University of Sharjah, University City Road, Sharjah, Sharjah 27272 United Arab Emirates; 4 Department of Computer Science, Faculty of Computers and Informatics, Zagazig University, Harayah Raznah, Zagazig, Sharqiyah 44519 Egypt

**Keywords:** Green mine evaluation, Spherical fuzzy sets, Multi-criteria decision-making (MCDM), SWOT analysis, CRITIC method, Grey relational analysis (GRA), Climate sciences, Environmental sciences, Environmental social sciences, Energy science and technology, Mathematics and computing

## Abstract

The development of green mines is essential for promoting sustainability in the mining sector due to the significant ecological impacts of resource extraction. This study proposes a novel hybrid multi-criteria decision-making (MCDM) framework that integrates Spherical Fuzzy Sets (SFSs) with SWOT analysis, the CRITIC method, and Grey Relational Analysis (GRA). The framework introduces several innovations: it applies SFS-based MCDM for the first time to green mine evaluation in Egypt, structures 37 sustainability-related criteria under SWOT dimensions, and employs SF-CRITIC for objective weighting without subjective comparisons. The model is applied to assess 20 gold mines, where the SF-GRA method is used to rank alternatives based on proximity to an ideal solution. The results show that GME20 consistently ranks highest, while GME5 ranks lowest. A sensitivity analysis is conducted by varying the Grey relational coefficient and simulating 37 weight scenarios, demonstrating stable rankings and strong model resilience. Comparative analysis against ten SFS-based MCDM methods confirms the consistency of results, with Spearman correlation coefficients exceeding 0.77. In addition to its methodological novelty, the framework supports interpretable decision outcomes by identifying key sustainability drivers such as renewable energy adoption and land reclamation. This contributes actionable insights for policymakers and stakeholders, enabling informed green investment and regulatory decisions. The study offers a transparent, reproducible, and scalable tool for sustainability evaluation in resource-intensive industries. The proposed model introduces a structured integration of SWOT-based criteria classification, objective weight computation via SF-CRITIC, and robust alternative ranking using SF-GRA. Furthermore, it contributes uniquely by applying the methodology to the underexplored context of green mine evaluation in Egypt. These distinctions articulate the methodological and application-based novelties of the proposed framework.

**Supplementary Information:**

The online version contains supplementary material available at 10.1038/s41598-025-21237-6.

## Introduction

Mineral resources are the backbone of several downstream sectors since they are vital raw materials. Nonetheless, ecological devastation, geological disasters, and land degradation are among the environmental challenges that arise during the extraction process. These challenges significantly hinder the sustainable growth of mining operations. In response, a growing number of researchers and policymakers have begun to focus on sustainable mining as a solution to resource depletion and environmental degradation^1,2^. In this context, the concept of “green mines” has been proposed. It represents a scientific and systematic approach to resource development and utilization that aims to minimize environmental impact while maximizing efficiency. Through green mine practices that follow established best practices across the mining life cycle, operators can reduce their ecological footprint, use resources more efficiently, and foster stronger community engagement^3,4^.

A thorough evaluation of operational green mines is a critical step toward planning future green mine initiatives. Evaluation of green mine performance involves the simultaneous consideration of multiple dimensions, such as environmental impact, economic viability, social responsibility, and technological readiness. These dimensions often conflict or trade off against each other, making an MCDM approach essential for structured and balanced decision-making. In the context of Egypt, such evaluations can generate economic benefits and support the development of effective policies for both governmental institutions and the mining industry. Multiple factors influence the performance of green mines, highlighting the need for a structured and systemic approach to ensure the long-term viability of the industry. Promoting green mine performance requires the development of frameworks that are both contextually relevant and methodologically robust. Establishing such systems is essential to address the complexity of sustainability challenges and to guide effective decision-making. There is a pressing need within both the corporate and public sectors for more in-depth studies that examine the internal and external factors influencing the performance of green mines across the continent. Previous research in this area has often been superficial and lacked the necessary detail to provide practical insights. There is a notable research gap concerning green mines in Egypt, where comprehensive documentation and actionable strategies for overcoming operational challenges are largely absent. Addressing this gap requires integrated studies that combine qualitative insights with robust multi-criteria decision-making (MCDM) methodologies to offer a holistic evaluation framework.

To address the identified gaps and support the development of a structured framework for evaluating green mines in Egypt, this study adopts advanced multi-criteria decision-making (MCDM) techniques. One core motivation is the increasing prominence of MCDM methods that incorporate fuzzy logic, which are particularly effective in managing uncertainty and vagueness in complex decision environments. Traditional fuzzy sets provide a basic means of modeling imprecise information, but they often fail to capture the hesitation and subjectivity present in expert judgments. Spherical Fuzzy Sets (SFSs) extend the capability of conventional fuzzy models by introducing three degrees of assessment: membership, non-membership, and hesitation. This enriched structure enables a more accurate representation of linguistic evaluations and expert uncertainty.

Another motivation relates to the importance of calculating criteria weights in MCDM processes. Methods such as the Analytic Hierarchy Process (AHP), the Best-Worst Method (BWM), Entropy, and the CRiteria Importance Through Intercriteria Correlation (CRITIC) method are widely used for this purpose. Unlike AHP, BWM, or Entropy, CRITIC does not rely on expert pairwise comparisons or subjective input. This study applies the spherical fuzzy extension of the CRITIC method, which integrates both the variation and correlation among criteria. CRITIC was chosen over other weighting methods due to its objectivity, computational simplicity, and compatibility with fuzzy environments, making it particularly suitable for integrating expert-derived decision matrices while minimizing subjective bias. Combined with the Strengths, Weaknesses, Opportunities, and Threats (SWOT) analysis, this approach enables the classification of evaluation criteria into four key categories, allowing for a more structured and insightful analysis of mining performance.

A final motivation concerns the need for a robust ranking mechanism to evaluate alternatives. Grey Relational Analysis (GRA) is a well-established MCDM method that measures the closeness of alternatives to an ideal solution. This study integrates GRA within the spherical fuzzy framework to prioritize gold mines in Egypt based on a comprehensive set of performance criteria. The proposed combination of SF-SWOT, SF-CRITIC, and SF-GRA addresses a critical gap in the literature, where the joint application of these methods remains largely unexplored in the context of green mine evaluation.

This study contributes significantly to sustainable mining evaluation by introducing a structured and uncertainty-aware decision-making framework. The data used in this research were collected through a combination of interviews, structured questionnaires, and expert consultations. The selected experts possess more than ten years of experience in the mining industry and hold academic qualifications, which ensures the reliability, validity, and contextual relevance of the data gathered for this evaluation. A significant contribution of this study is its introduction of SWOT analysis as a structured framework for assessing green mines. To the best of the authors’ knowledge, no prior research has applied SWOT analysis specifically to green mine or systematically divided the criteria into the four main SWOT categories: Strengths, Weaknesses, Opportunities, and Threats. This study places particular emphasis on analyzing weaknesses and threats, thereby filling a methodological and empirical gap in the literature. The insights generated from this approach are expected to benefit researchers and practitioners interested in the design, implementation, and management of green mine projects.

Another key contribution is the use of Spherical Fuzzy Sets (SFSs) to address hesitation, imprecision, and subjectivity in expert assessments. A total of thirty-seven evaluation criteria were developed across the four SWOT dimensions, based on both expert input and prior studies. These criteria were objectively weighted using the SF-CRITIC method, which accounts for both variability and correlation among indicators. Subsequently, the SF-GRA method was used to rank the alternatives, providing a systematic approach to selecting the most appropriate green mine. Although the study focuses specifically on gold mines in Egypt, the proposed framework is adaptable and can be extended to other mining sectors, such as coal, copper, and iron. This generalizability increases the practical utility and transferability of the methodology. In addition, the findings offer valuable decision support for government agencies and mining firms by identifying the most suitable mine for green conversion and guiding sustainability-oriented investments and policies.

The study is innovative in its integration of SWOT analysis with the SF-CRITIC and SF-GRA techniques. This combination forms a novel and cohesive framework that is capable of handling uncertainty and imprecision, while offering a structured approach to performance assessment in green mine. The real-world applicability of the model is demonstrated through a case study conducted in Egypt, which validates the framework’s effectiveness and practical value. Finally, both sensitivity and comparative analyses are carried out to evaluate the robustness and stability of the proposed methodology. These analyses confirm that the model produces consistent and reliable results under varying input scenarios and when compared against other decision-making methods. Overall, the study contributes a replicable and comprehensive approach to green mine evaluation under uncertainty. The primary objective of this study is to develop an efficient decision-making model for assessing green mine performance. This includes identifying key criteria and sub-criteria through the SWOT analysis framework and designing a structured process that supports informed and sustainable decisions under uncertainty. The proposed model is applied to a practical case study in Egypt to identify the most appropriate decision-making framework for green mine assessment, determine the most influential sustainability-related weaknesses and threats, and define a comprehensive set of evaluation factors and alternatives.

In summary, this study addresses a methodological gap in the literature by integrating SWOT analysis, the CRITIC method, and Grey Relational Analysis (GRA) within the Spherical Fuzzy Sets (SFSs) environment. This combination has not been previously applied to the evaluation of green mines. The main contributions of this research include the development of a hybrid SFS-based multi-criteria decision-making framework for assessing green mine performance under uncertainty, its application to a real-world case involving 20 gold mines in Egypt evaluated against 37 sustainability-related criteria, the use of SWOT analysis as a structured basis for criteria classification, and the validation of the framework’s robustness through both sensitivity and comparative analyses. The proposed approach supports sustainable decision-making in the mining sector and offers a replicable model that can be adapted to other contexts.

The remainder of this paper is organized as follows. “Literature review” presents a comprehensive literature review covering the theoretical foundations of Spherical Fuzzy Sets (SFSs), SWOT analysis, the CRITIC method, Grey Relational Analysis (GRA), and their application in green mine evaluation. “Problem definition” describes the development of the evaluation framework, detailing the SWOT-based criteria used in the study. The proposed methodology is outlined in “Methodology”, which integrates the SF-SWOT, SF-CRITIC, and SF-GRA techniques into a unified decision-making model. “Application” presents the case study and the results of applying the methodology to selected gold mines in Egypt. “Sensitivity analysis” and “Comparative analysis” provide the sensitivity analysis and comparative analysis, respectively, to evaluate the robustness of the results. “Analytical insights and strategic implications” discusses the findings and their practical implications. Finally, “Conclusions and future works” presents the study’s conclusions and outlines directions for future research.

## Literature review

This section presents a review of the relevant literature related to the study. The focus is on the key concepts and methodologies used in evaluating green mines, including spherical fuzzy sets (SFSs), SWOT analysis, CRITIC method, and Grey Relational Analysis (GRA). The review highlights the importance of these techniques in addressing uncertainty, vagueness, and imprecision in decision-making processes, particularly in the context of sustainable mining practices.

### Spherical fuzzy sets (SFSs)

Fuzzy set theory was introduced by Zadeh^5^ in 1965 to deal with vague, uncertain, inaccurate, and inconsistent systems. While classical fuzzy sets are suitable for representing partial membership, they are limited in capturing dissatisfaction or hesitancy in decision-making contexts. So, Atanassov^6^ introduced intuitionistic fuzzy sets (IFSs) with degrees of truth and falsity. The IFSs deal perfectly in the uncertain and vague data but this framework cannot deal with values of total membership and non-membership greater than 1, which limits their flexibility in modeling more complex forms of uncertainty. To overcome this limitation, Yager^7^ introduced the Pythagorean fuzzy sets (PFSs). The PFSs deal perfectly with a large amount of uncertainty and work well to solve the problems concerned with the IFSs. PFSs relax the constraint by allowing the squared sum of membership and non-membership degrees to remain less than or equal to one. PFSs provide a broader framework for capturing uncertainty but still do not explicitly represent hesitancy.

In parallel to this development, interval type-2 fuzzy sets (IT2FSs) were introduced to capture higher levels of uncertainty by employing secondary membership functions^8^. IT2FSs enhance the expressiveness of fuzzy modeling in situations with significant ambiguity, but they often lead to increased computational complexity and reduced interpretability in practical decision-making contexts. Similarly, hesitant fuzzy sets (HFSs) were proposed to allow decision-makers to express multiple possible membership values for an element when exact assessment is difficult^9^. HFSs are effective in capturing indecision, but they do not incorporate explicit degrees of falsity or indeterminacy, which limits their applicability in fully uncertain environments.

Subsequently, Cuong and Kreinovich^10^ developed picture fuzzy sets (PIFSs), which include a third parameter for neutrality and allow the representation of satisfaction, dissatisfaction, and abstinence. While PIFSs improve descriptive capability, they lack the geometric flexibility needed for more comprehensive uncertainty modeling. Spherical fuzzy sets (SFSs), introduced by Gündogdu and Kahraman^11^, provide a more advanced structure by incorporating three parameters: truth, falsity, and hesitation. These parameters are subject to a spherical condition, ensuring that their squared sum does not exceed one. This formulation allows SFSs to represent uncertainty more flexibly than prior fuzzy models. Applications of SFSs can be found in diverse domains such as social banking^12^, solid waste^13^, supply chain management^14^, and electric vehicle assessment^15^.

Compared to other fuzzy set extensions such as interval type-2 fuzzy sets (IT2FSs)^8^, hesitant fuzzy sets (HFSs)^9^, and Pythagorean Fuzzy Subsets (PFSs)^7^, SFSs provide a more balanced representation of uncertainty with computational efficiency. While IT2FSs offer high precision in uncertainty modeling, they often require increased computational effort. HFSs permit multiple membership values but do not explicitly represent non-membership or hesitancy in a unified structure. Although PFSs extend IFSs by broadening the membership space, they remain limited in expressing hesitation. SFSs incorporate membership, non-membership, and hesitancy simultaneously within a single normalized framework, making them particularly effective for decision-making situations that involve vagueness, expert subjectivity, and conflicting criteria. Recent developments have introduced modified SFS-based decision-making frameworks that extend traditional models to accommodate uncertainty in service evaluations and logistical platforms. These studies highlight the growing adoption of advanced fuzzy variants in real-world assessment contexts^16^.

As explored in the literature review, no prior study has applied spherical fuzzy sets (SFSs) to the evaluation of green mine performance. This study is therefore the first to integrate an MCDM approach with SFSs for assessing sustainability in the mining sector. The proposed framework incorporates multiple analytical tools, including SWOT analysis, the CRITIC method, and Grey Relational Analysis (GRA), to structure, weight, and rank evaluation criteria. The following subsection introduces the SWOT method, which serves as the foundation for organizing the internal and external factors used in the assessment.

### SWOT method

The SWOT analysis framework consists of four dimensions that help identify and evaluate both internal and external factors influencing a decision problem: strengths, weaknesses, opportunities, and threats. It provides a structured means for organizing complex decision criteria and has been widely applied in strategic planning and sustainability-related evaluations^17^. Recent applications have utilized SWOT-based criteria structuring within fuzzy decision environments to address labor market and public policy challenges^18^. These developments demonstrate the adaptability of SWOT frameworks across domains beyond industrial and environmental engineering.

In one study, SWOT was combined with the Analytic Hierarchy Process (AHP) and the MARCOS method to explore strategies for digital transformation^19^. The integration was performed within a fuzzy environment to address uncertainty in expert judgments. In this model, the AHP method was used to derive the weights of SWOT-based criteria, while the fuzzy MARCOS method was applied to rank the strategic alternatives. However, one limitation of this approach was its assumption of independence among criteria, which may not hold in practical scenarios where interrelationships exist. Another study integrated SWOT with fuzzy AHP and the Technique for Order of Preference by Similarity to Ideal Solution (TOPSIS) to develop strategic models for telemedicine^20^. SWOT analysis was employed to identify critical conditions, and the AHP method was used to assign weights to each SWOT dimension. Subsequently, fuzzy TOPSIS was applied to prioritize the strategies. Although effective in structuring the decision-making process, the study did not include comparisons with alternative MCDM methods, leaving the robustness of the proposed model unverified.

In the transportation domain, SWOT was integrated with interval type-2 triangular fuzzy AHP (ITTF-AHP) to evaluate sector-specific factors^21^. In this case, SWOT was used to define the decision criteria, and the ITTF-AHP method computed their relative importance under high uncertainty conditions. Similarly, another application of SWOT in the dimension stone industry combined it with Delphi, AHP, and fuzzy TOPSIS methods to support strategic planning^22^. The process began with expert input through Delphi, followed by AHP to calculate criteria weights, and concluded with fuzzy TOPSIS to rank the proposed strategies. All components were implemented under a fuzzy environment to address vagueness and imprecision.

The review of the existing literature demonstrates that SWOT analysis has been widely used in combination with various fuzzy and MCDM techniques across diverse application areas. However, despite the increasing attention to uncertainty modeling in decision-making, no study has yet incorporated the SWOT framework within the context of spherical fuzzy sets. This study addresses that gap by being the first to implement SWOT analysis under a spherical fuzzy environment, enabling a more nuanced and flexible representation of expert assessments involving vagueness, ambiguity, and hesitation in green mine evaluation.

### CRITIC method

The CRiteria Importance Through Intercriteria Correlation (CRITIC) method is a multi-criteria decision-making (MCDM) approach that computes objective weights for decision criteria based on the contrast intensity and the correlations among criteria within a decision matrix. It does not rely on subjective judgments, making it particularly suitable for cases where data are available and objective weighting is required. The method has been widely adopted in various application domains and has also been extended to support decision-making under fuzzy and spherical fuzzy environments. In one study, a hybrid decision-making model was proposed that integrated the CRITIC method with the REGIME method for solving a treatment selection problem in the context of breast cancer^23^. The CRITIC method was used to compute the objective weights of evaluation criteria, while the model was applied under special fuzzy sets to accommodate uncertainty in the decision-making environment. The integration allowed for a systematic and data-driven evaluation of alternative treatment options. Another study employed both the CRITIC and WASPAS methods within an interval-valued spherical fuzzy environment to assess medical waste disposal alternatives^24^. The model was designed to capture the uncertainty inherent in expert evaluations, and the CRITIC method was used to derive weights that reflect both the variability and interdependence of criteria. The WASPAS method was subsequently applied to rank the disposal options, providing a structured approach to a complex healthcare waste management problem.

In the field of supply chain and procurement, the CRITIC method was utilized to determine the relative importance of supplier selection criteria under a fuzzy environment^25^. The model supported more objective decision-making by leveraging the statistical characteristics of the data, rather than relying on expert preference alone. Another study proposed a hybrid model that combined the CRITIC and MARCOS methods under spherical fuzzy sets to assist in smartphone selection decisions^26^. In this case, the CRITIC method provided a robust weighting mechanism, while the MARCOS method enabled detailed ranking of alternatives based on the computed weights.

These studies highlight the flexibility of the CRITIC method and its effectiveness in various decision-making contexts, especially when integrated with other MCDM methods. Its application under fuzzy and spherical fuzzy environments further enhances its ability to handle ambiguity, uncertainty, and imprecise information, which are common in real-world problems related to healthcare, environmental management, and consumer product evaluation. These characteristics make CRITIC well suited for sustainability evaluation problems where large sets of interrelated criteria must be weighted objectively and consistently. In the current study, CRITIC is used as the foundation for deriving robust and bias-minimized weights prior to applying a separate ranking method.

### GRA method

Grey Relational Analysis (GRA) is a widely used multi-criteria decision-making (MCDM) technique designed to rank alternatives by measuring the degree of similarity or closeness between each alternative and an ideal reference. It is particularly useful in complex decision environments where data may be incomplete, uncertain, or imprecise. GRA transforms multiple performance attributes into a single relational coefficient, allowing decision-makers to systematically compare alternatives. In recent years, GRA has been applied across a broad range of fields and adapted to operate within fuzzy and spherical fuzzy environments to better address uncertainty.

In one study, the GRA method was applied alongside the Analytic Hierarchy Process (AHP) under a spherical fuzzy environment to assess sustainable industrialization performance within the European Union^27^. The AHP method was used to derive the weights of the evaluation criteria, while GRA facilitated the ranking of countries based on their performance scores. The integration of these methods helped handle the complexity of socio-economic data and account for uncertainty in the sustainability indicators. Another application involved the development of a decision-making model to address emergency planning during the COVID-19 pandemic. This model incorporated the GRA method under spherical fuzzy sets to manage the ambiguity and hesitancy inherent in expert judgments during crisis conditions^28^. The approach enabled effective prioritization of response strategies based on multiple, uncertain criteria related to public health and resource allocation. A recent hybrid model has combined spherical fuzzy sets with GRA MARCOS to evaluate expert-driven performance indicators in academic or institutional settings^29^, showcasing the methodological adaptability of fuzzy-ranking mechanisms.

In the domain of pattern recognition, GRA has also been used within a hesitant fuzzy environment to improve classification performance^30^. The fuzzy GRA method was utilized to compute the closeness between observed patterns and reference classes, enhancing recognition accuracy under uncertain input conditions. This demonstrates GRA’s versatility beyond traditional decision-making and into technical domains such as artificial intelligence and image analysis. A further example applied the GRA method under an interval-valued fuzzy environment to support decision-making in supply chain management^31^. This study combined GRA with quality function deployment to identify and prioritize key supply chain drivers, accounting for vagueness in customer requirements and performance measures. In the software engineering field, GRA was adopted under fuzzy sets to estimate software development effort by analyzing the relational distance between historical project data^32^. This use of GRA helped reduce uncertainty and improve prediction accuracy in project planning, where estimation errors are typically high.

These studies demonstrate the robustness of GRA as an MCDM method capable of addressing uncertainty in diverse application areas. Its integration with fuzzy and spherical fuzzy sets enables more accurate and flexible modeling of decision-maker preferences, making it a suitable tool for ranking alternatives in sustainability, healthcare, technology, and engineering contexts.

### Methods for evaluation performance green mines

The evaluation of green mine performance is inherently a complex decision-making process that involves multiple environmental, economic, operational, and social criteria. As a result, numerous multi-criteria decision-making (MCDM) methods have been introduced in the literature to support robust and informed assessments. These methods vary in terms of structure, input requirements, and ability to handle uncertainty. In one study, a hybrid decision-making framework was proposed that combined Grey DEMATEL and the Analytic Network Process (ANP) to assess green mine performance^33^. The model was applied to six gold mines, with the aim of identifying key factors influencing sustainability and formulating strategic directions for improvement. The use of Grey DEMATEL enabled the mapping of causal relationships among criteria, while ANP supported the evaluation of interdependent criteria through a network-based structure.

Another study employed a data envelopment analysis (DEA) model to evaluate the efficiency of green mines in China’s coal sector^34^. This non-parametric technique assessed the relative performance of decision-making units (mines) by comparing inputs and outputs. The approach highlighted performance gaps and provided benchmarking insights for operational enhancement in resource-intensive sectors. An integrated MCDM model was also developed that combined the Analytic Hierarchy Process (AHP) and Grey clustering techniques to promote green mine practices^35^. The model organized 24 evaluation indicators into four main categories and was applied to a case study involving a green mine in China. AHP was used to determine the relative importance of each criterion, while Grey clustering supported the classification of mines based on their performance scores under uncertainty. A separate investigation proposed a methodology using hesitant fuzzy sets to assess green mine performance^36^. This model integrated the QUALIFLEX and ORESTE methods to capture qualitative and rank-based evaluation preferences. The methodology was applied to phosphate mines, allowing for the comparison of multiple alternatives despite the presence of hesitation in expert assessments.

Another contribution introduced a composite MCDM framework combining TODIM, the Best-Worst Method (BWM), and ELECTRE under a picture fuzzy environment^37^. The TODIM method was used to reflect decision-makers’ psychological behavior, while BWM and ELECTRE were employed to derive criteria weights and conduct outranking analysis, respectively. The framework was designed to offer a holistic and flexible approach to mine performance evaluation under uncertainty. These studies illustrate the diverse range of MCDM techniques applied in the evaluation of green mine performance. They emphasize the need for methodological frameworks that can handle multiple dimensions of sustainability, account for uncertainty, and adapt to various types of decision environments. Despite these advances, the integration of spherical fuzzy sets into green mine evaluation remains largely unexplored, reinforcing the novelty and relevance of the approach proposed in this study.

## Problem definition

This section presents the set of evaluation criteria used to assess the performance of green gold mines in Egypt. A total of 37 criteria were identified and classified into the four SWOT dimensions: Strengths, Weaknesses, Opportunities, and Threats. The criteria reflect internal and external factors influencing sustainable mining performance and were developed through expert consultation, interviews, and review of previous studies^36–39^.

### Strength (STR)

The strengths dimension represents internal capabilities and attributes of mining operations that contribute positively to environmental sustainability and operational efficiency. These criteria highlight best practices, resource utilization, and corporate strategies that support the transformation toward green mine. The following ten factors were identified as key internal strengths:

STR1: Efficient Resource Use—The mine optimizes the utilization of natural resources, leading to decreased waste generation and improved operational efficiency.

STR2: Biodiversity Conservation—The mine implements effective measures to conserve and enhance local biodiversity through targeted conservation initiatives.

STR3: Stakeholder Engagement—The mining company actively engages with local communities to build transparent, positive relationships.

STR4: Corporate Management—The company demonstrates effective internal governance, including data-driven decision-making processes and strategic oversight.

STR5: Environmental Monitoring and Land Restoration—The mine monitors land and water conditions and implements restorative measures to rehabilitate degraded sites.

STR6: Land Reclamation Program Implementation—This factor reflects the existence and quality of a land reclamation program, as evaluated by expert judgment considering the applied technology, financial investment, and performance outcomes.

STR7: Renewable Energy Adoption—The mine utilizes renewable energy sources such as solar or wind to reduce carbon emissions and dependency on fossil fuels.

STR8: Feasibility of Land Reclamation—Experts assess the practicality, availability, and effectiveness of land reclamation projects implemented at the site.

STR9: Contribution to Community Infrastructure—The mine contributes funding or resources to support local infrastructure, including hospitals, schools, and public services.

STR10: Land Reclamation Rate—This factor is measured by the ratio of successfully reclaimed land to the total area affected by mining activities.

### Opportunities (OPP)

The opportunities dimension captures external conditions or trends that can enhance the sustainability and competitiveness of mining operations. These include technological advances, favorable policies, market incentives, and stakeholder collaboration that support green transformation. The criteria below outline eleven major opportunities that gold mines can leverage to improve environmental and economic outcomes:

OPP1: Collaboration and Partnerships—The mine fosters sustainability through partnerships with academic institutions, NGOs, or government agencies to promote best practices and innovation.

OPP2: Government Incentives—The mine benefits from financial grants or policy incentives provided by the government to improve ecological performance.

OPP3: Corporate Culture—This factor captures the values, ethics, and environmental responsiveness of employees, as reflected in company-wide behavior and performance.

OPP4: Market Demand—The growing preference for eco-friendly products offers green mine companies a competitive advantage among environmentally conscious investors and consumers.

OPP5: Technical Culture—Evaluated by experts, this includes technical training, employee development, and workplace facilities that reflect a modern industrial culture.

OPP6: Emission Reduction—The mine actively reduces emissions of pollutants, waste, and energy during operations to minimize atmospheric contamination.

OPP7: Mining Recovery Rate—Calculated as the ratio of gold extracted during a given period to the total amount available for extraction.

OPP8: Utilization Rate of Gold Gangue—Represents the proportion of gold gangue reused or recycled in the production process.

OPP9: Technological Innovation in Resource Mining—Reflects the level of investment in green technology and innovation, which enhances operational efficiency and reduces environmental impacts.

OPP10: Raw Gold Washing Rate—The ratio of washed gold to total raw gold processed during the evaluation period.

OPP11: Utilization of Co-Associated Components—The ratio of recovered valuable by-products to their total potential yield during mining and processing activities.

### Threats (THR)

The threats dimension encompasses external risks or uncertainties that may hinder the adoption or long-term success of green mine practices. These factors range from environmental incidents to regulatory volatility and reputational challenges. The following ten threats were identified as critical risks that must be addressed in sustainability assessments:

THR1: Environmental Incidents—The occurrence of ecological accidents that negatively impact the environment during mining operations.

THR2: Market Risk—The risk of diminished investor confidence due to volatility or instability in mining markets.

THR3: Regulatory Changes—Policy or regulatory shifts that increase operational costs or impose additional environmental constraints.

THR4: Public Perception—Negative public sentiment regarding mining practices, which may damage the company’s reputation and social license to operate.

THR5: Community Harmony—The degree to which the mining operation supports local well-being through initiatives in health, education, or environmental protection.

THR6: SO_2_ Emissions—The amount of Sulfur dioxide released per unit of mining output over a specified period of time.

THR7: Reputational Risk—Threats to the company’s reputation that may lead to a loss of stakeholder support and investment.

THR8: COD Emissions—Chemical Oxygen Demand (COD) emissions generated during mining operations, normalized by production volume.

THR9: Financial Viability Challenges—Risks to the economic sustainability of green mine due to fluctuating market prices and demand.

THR10: Increased Operating Costs—Rising operational and capital costs that may threaten the long-term feasibility of mining activities.

### Weakness (WEA)

The weaknesses dimension refers to internal deficiencies or limitations that reduce the efficiency, compliance, or community alignment of mining operations. These factors may impede the mine’s ability to operate sustainably or transition toward green practices. The criteria listed below represent six common weaknesses found in mining performance:

WEA1: Solid Waste Generation—The volume of solid waste produced during mining operations relative to total output.

WEA2: Wastewater Discharge Rate—The amount of wastewater produced relative to total water usage and processing volume.

WEA3: Limited Stakeholder Engagement—Inadequate interaction with local stakeholders, which may lead to conflict or mistrust.

WEA4: Environmental Compliance Risk—The potential for reputational and regulatory consequences arising from non-compliance with environmental laws.

WEA5: Energy Consumption—The total energy used per unit of production, indicating operational efficiency and environmental impact.

WEA6: Water Consumption—The total water consumed per unit of output, measured to assess the sustainability of water use in mining operations.

## Methodology

This study presents an integrated decision-making framework to assess the performance of green gold mines in Egypt. The framework incorporates both internal and external evaluation criteria, which are identified through expert interviews, a comprehensive literature review, and sector-specific data. The primary objective is to support the selection and prioritization of the most suitable gold mine for green conversion under conditions of uncertainty, using a spherical fuzzy multi-criteria decision-making approach.

**Fig. 1:**
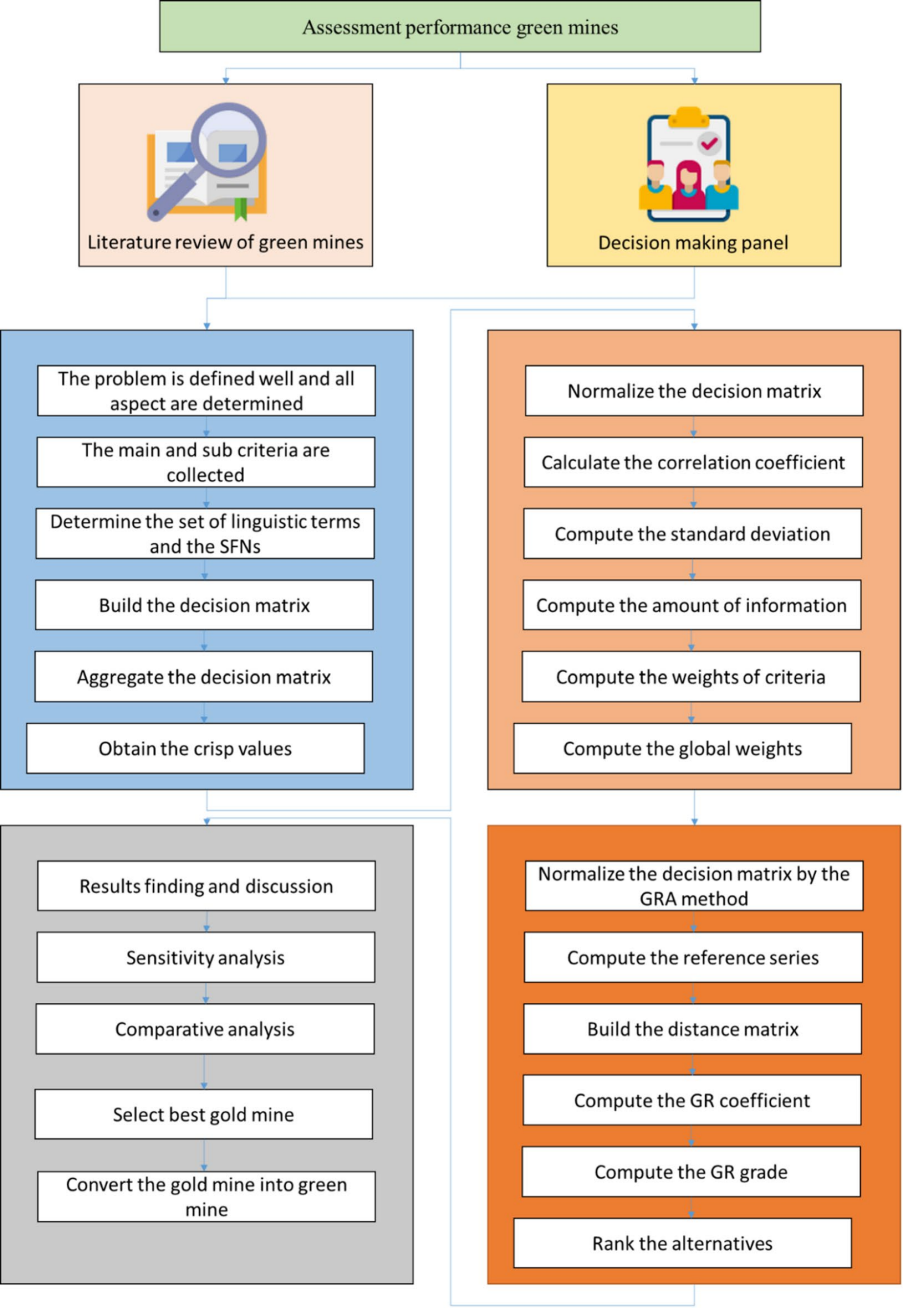
The process of the SFSs-based methodology.

Figure 1 presents a comprehensive overview of the proposed methodology. The process begins with two initial inputs: a literature review of green mine evaluation practices (Block 1, on the left) and the formation of a decision-making panel (Block 2, on the right). These inputs guide the identification of evaluation criteria and the development of linguistic terms, which are modeled using the Spherical Fuzzy Sets (SFSs) framework. Block 3 outlines the steps for constructing and aggregating the decision matrix, beginning with problem definition and progressing through the formulation of criteria, linguistic scale selection, and the generation of crisp values. Block 4 presents the use of the SF-CRITIC method, which includes matrix normalization, calculation of correlation coefficients and standard deviations, and computation of both local and global criteria weights. Block 5 illustrates the SF-GRA procedure, where the normalized decision matrix is processed to compute grey relational coefficients and grades, ultimately producing a ranked list of alternatives. Block 6 depicts the final phase, where the results are interpreted through sensitivity and comparative analyses, leading to the selection of the most suitable gold mine for green conversion.

The methodology integrates three core components: (1) criteria identification and structuring using SF-SWOT, (2) objective weight calculation using SF-CRITIC, and (3) alternative ranking using SF-GRA. These components are interlinked through a systematic data flow that ensures consistency, traceability, and robustness in decision-making. The following subsections provide the theoretical basis and procedural details of each step.

### Preliminaries

The Spherical Fuzzy Sets (SFSs) were introduced by Kutlu Gundogdu and Kahraman^40^. The SFSs are based on the concept of hesitation from decision-makers, and they incorporate three degrees: membership, non-membership, and hesitation. The sum of these three degrees is always equal to 1. The basic arithmetic operations of SFSs are outlined in^25^ and^40^.

#### Definition 1

The SFSs can be offered with the universe of discourse $\:U$ as:

1 $$\:{F}_{S}=\left\{\left(u,\:\left({X}_{{F}_{S}}\right),\left({Y}_{{F}_{S}}\right),\left({Z}_{{F}_{S}}\right)\right)|\:u\in\:U\:\right\}$$

where the three functions $\:\left({X}_{{F}_{S}}\right),\left({Y}_{{F}_{S}}\right),\left({Z}_{{F}_{S}}\right)$ which refer to the membership, non-membership, and hesitancy degrees are:

2 $$\begin{aligned}&{X}_{{F}_{S}}\left(u\right):U\to\:\left[\text{0,1}\right],\\ & {Y}_{{F}_{S}}\left(u\right):U\to\:\left[\text{0,1}\right],\\ &{Z}_{{F}_{S}}\left(u\right):U\to\:\left[\text{0,1}\right] \text{ and},\\ & 0\le\:{X}_{{F}_{S}}^{2}\left(u\right)+{Y}_{{F}_{S}}^{2}\left(u\right)+{Z}_{{F}_{S}}^{2}\left(u\right)\le\:1\:\forall\:u\in\:U\end{aligned}$$

These constraints ensure that the combination of membership, non-membership, and hesitation degrees lies within the surface of a unit sphere in 3D space. This formulation generalizes the concept of uncertainty by allowing all three degrees to coexist within a normalized structure.

The following arithmetic operations define how two spherical fuzzy numbers are combined. Addition and multiplication rules are adapted to preserve the spherical constraint.

#### Definition 2

Let $\:{{F}_{s}}_{1}=\left({X}_{{{F}_{S}}_{1}},{Y}_{{{F}_{S}}_{1}},{Z}_{{{F}_{S}}_{1}}\right)\:and\:{{F}_{s}}_{2}=\left({X}_{{{F}_{S}}_{2}}{,Y}_{{{F}_{S}}_{2}},{Z}_{{{F}_{S}}_{2}}\right)$ be two spherical fuzzy numbers. Then the arithmetic operations are defined as follows:

3 $$\:{{F}_{s}}_{1}\oplus\:\:{{F}_{s}}_{2}=\left\{\begin{array}{c}\:{\left({X}_{{{F}_{S}}_{1}}^{2}+{X}_{{{F}_{S}}_{2}}^{2}-{X}_{{{F}_{S}}_{1}}^{2}{X}_{{{F}_{S}}_{2}}^{2}\right)}^{0.5\:}\\\:\:{Y}_{{{F}_{S}}_{1}}{Y}_{{{F}_{S}}_{2}},\:\\\:\:{\left(\left(1-{X}_{{{F}_{S}}_{2}}^{2}\right){Z}_{{{F}_{S}}_{1}}^{2}+\left(1-{X}_{{{F}_{S}}_{1}}^{2}\right){Z}_{{{F}_{S}}_{2}}^{2}-{Z}_{{{F}_{S}}_{1}}^{2}{Z}_{{{F}_{S}}_{2}}^{2}\right)}^{0.5},\end{array}\right\}$$

The addition formula enhances the combined membership degree while maintaining a minimal non-membership and a calculated hesitancy based on how dissimilar the inputs are

4 $$\:{{F}_{s}}_{1}\otimes\:\:{{F}_{s}}_{2}=\left\{\begin{array}{c}{X}_{{{F}_{S}}_{1}}{X}_{{{F}_{S}}_{2}},\\\:\:{\left({Y}_{{{F}_{S}}_{1}}^{2}+{Y}_{{{F}_{S}}_{2}}^{2}-{Y}_{{{F}_{S}}_{1}}^{2}{Y}_{{{F}_{S}}_{2}}^{2}\right)}^{0.5},\\\:{\left(\left(1-{Y}_{{{F}_{S}}_{2}}^{2}\right){Z}_{{{F}_{S}}_{1}}^{2}+\left(1-{Y}_{{{F}_{S}}_{1}}^{2}\right){Z}_{{{F}_{S}}_{2}}^{2}-{Z}_{{{F}_{S}}_{1}}^{2}{Z}_{{{F}_{S}}_{2}}^{2}\right)}^{0.5}\end{array}\right\}$$

In multiplication, the membership degree is multiplied directly, while non-membership and hesitation are adjusted in a way that preserves their relationship with membership, thus reflecting compounded uncertainty.

The power operation defines the influence of a scalar $\:\zeta\:$ on a spherical fuzzy number, which is useful in aggregation or attenuation of fuzzy values.

5 $$\:\zeta\:{{F}_{s}}_{1}=\left\{\begin{array}{c}\:{\left(1-{\left(1-{X}_{{{F}_{S}}_{1}}^{2}\right)}^{\zeta\:}\right)}^{0.5},\\\:{Y}_{{{F}_{S}}_{1}}^{\zeta\:}\:,\\\:{\left({\left(1-{X}_{{{F}_{S}}_{1}}^{2}\right)}^{\zeta\:}-{\left(1-{X}_{{{F}_{S}}_{1}}^{2}-{Z}_{{{F}_{S}}_{1}}^{2}\right)}^{\zeta\:}\right)}^{0.5}\:\:\end{array}\right\}\:for\:\zeta\:\ge\:0$$

This allows modeling confidence or emphasis on a fuzzy number: higher ($\:\zeta\:$) reduces uncertainty when values are strong and amplifies distinction when they are weak.

The following definitions provide the weighted aggregation of multiple SFSs, which is essential when combining expert opinions or multi-criteria evaluations. The Weighted Arithmetic Mean (WAM) increases membership when multiple SFSs agree and distributes uncertainty through geometric transformations of hesitation and non-membership values.

#### Definition 3

The Weighted Arithmetic Mean (WAM) is defined as:

6 $$\begin{aligned}&\:WA{M}_{w}\left({{F}_{s}}_{1},{{F}_{s}}_{2},\dots\:.{{F}_{s}}_{n}\right)={w}_{1}{{F}_{s}}_{1}+{w}_{2}{{F}_{s}}_{2}+\dots\:+{{{w}_{n}F}_{s}}_{n}\\ & \quad =\:\left\{\begin{array}{c}{\left(1-\prod\:_{i=1}^{n}{\left(1-{X}_{{{F}_{S}}_{1}}^{2}\right)}^{{w}_{i}}\right)}^{0.5},\:\\\:\prod\:_{i=1}^{n}{Y}_{{{F}_{S}}_{1}}^{{w}_{i}},\:\\\:{\left(\prod\:_{i=1}^{n}{\left(1-{X}_{{{F}_{S}}_{1}}^{2}\right)}^{{w}_{i}}-\prod\:_{i=1}^{n}{\left(1-{X}_{{{F}_{S}}_{1}}^{2}-{Z}_{{{F}_{S}}_{1}}^{2}\right)}^{{w}_{i}}\right)}^{0.5}\end{array}\right\}\end{aligned}$$

where $\:{w}_{i}$ refers to the weights of experts, $\:w=\left({w}_{1},{w}_{2},\dots\:\dots\:,\:{w}_{2}\right);{w}_{i}\in\:\left[\text{0,1}\right];\:\sum\:_{i=1}^{n}{w}_{i}=1$.

The Weighted Geometric Mean (WGM) is more conservative in membership accumulation and emphasizes consistency across all experts or criteria by using multiplicative behavior.

#### Definition 4

The Weighted Geometric Mean (WGM) is defined as:

7 $$\begin{aligned}&\:{\text{W}\text{G}\text{M}}_{\text{w}}\left({{F}_{s}}_{1},{{F}_{s}}_{2},\dots\:.{{F}_{s}}_{n}\right)={{F}_{s}}_{1}^{{w}_{1}}+{{F}_{s}}_{2}^{{w}_{2}},\dots\:.{{F}_{s}}_{n}^{{w}_{n}}\\ & \quad =\:\left\{\begin{array}{c}\prod\:_{i=1}^{n}{X}_{{{F}_{S}}_{1}}^{{w}_{i}}\:,\\\:\:{\left(1-\prod\:_{i=1}^{n}{\left(1-{Y}_{{{F}_{S}}_{1}}^{2}\right)}^{{w}_{i}}\right)}^{0.5},\\\:\left(\prod\:_{i=1}^{n}{\left(1-{Y}_{{{F}_{S}}_{1}}^{2}\right)}^{{w}_{i}}-\prod\:_{i=1}^{n}{\left(1-{Y}_{{{F}_{S}}_{1}}^{2}-{Z}_{{{F}_{S}}_{1}}^{2}\right)}^{{w}_{i}}\right)\end{array}\right\}\end{aligned}$$

The score function evaluates the net favorability of an SFS, weighing membership against non-membership and penalizing hesitation. The accuracy function measures the completeness of information contained in an SFS. Together, they help compare alternatives: the higher the score and accuracy, the better the alternative.

#### Definition 5

Let $\:FS=(X,Y,Z)$ be a spherical fuzzy number. Then the score ($\:SC)$ and accuracy ($\:ACC)$ functions are defined as:

8 $$\:SC\left({F}_{{s}_{1}}\right)=\left({X}_{{{F}_{S}}_{1}}-\frac{{Z}_{{{F}_{S}}_{1}}}{2}\right)-{\left({Y}_{{{F}_{S}}_{1}}-\frac{{Z}_{{{F}_{S}}_{1}}}{2}\right)}^{2}$$

9 $$\:ACC\left({F}_{{s}_{1}}\right)={X}_{{{F}_{S}}_{1}}^{2}\:+{Y}_{{{F}_{S}}_{1}}^{2}+{Z}_{{{F}_{S}}_{1}}^{2}$$

Then, the comparison between $\:{F}_{{s}_{1}}$ and $\:{F}_{{s}_{2}}$​​ is defined as $\:{F}_{{s}_{1}}<{F}_{{s}_{2}}\:$if and only if:

$$\:SC\left({F}_{{s}_{1}}\right)<SC\left({F}_{{s}_{2}}\right)\:or$$

$$\:SC\left({F}_{{s}_{1}}\right)=SC\left({F}_{{s}_{2}}\right)\:\text{a}\text{n}\text{d}\:ACC\left({F}_{{s}_{1}}\right)<ACC\left({F}_{{s}_{2}}\right)$$

The distance function quantifies the dissimilarity between two SFSs in terms of all three dimensions (membership, non-membership, hesitation). This normalized measure captures both the magnitude and direction of divergence between two fuzzy numbers.

#### Definition 6

The distance between two spherical fuzzy numbers is defined as:

10 $$\:{D}^{s}\left({F}_{{s}_{1}},{F}_{{s}_{2}}\right)=1-\frac{{X}_{{{F}_{S}}_{1}}^{2}\times\:{X}_{{{F}_{S}}_{2}}^{2}+{Y}_{{{F}_{S}}_{1}}^{2}\times\:{Y}_{{{F}_{S}}_{2}}^{2}+{Z}_{{{F}_{S}}_{1}}^{2}\times\:{Z}_{{{F}_{S}}_{2}}^{2}}{{X}_{{{F}_{S}}_{1}}^{4}\vee\:{X}_{{{F}_{S}}_{2}}^{4}+{Y}_{{{F}_{S}}_{1}}^{4}\vee\:{Y}_{{{F}_{S}}_{2}}^{4}+{Z}_{{{F}_{S}}_{1}}^{4}\vee\:{Z}_{{{F}_{S}}_{2}}^{4}}$$

This function is particularly relevant in decision-making models that require comparisons between alternatives and a reference point. In this study, the distance function is employed within the SF-GRA method to construct a distance matrix between each alternative and the reference series. This matrix provides the foundation for calculating the Grey relational coefficients used to rank alternatives. By incorporating the three dimensions of membership, non-membership, and hesitation, this measure captures the full spectrum of expert uncertainty and enhances the accuracy of the decision analysis.

### SF-SWOT-CRITIC-GRA

This study proposes SF-SWOT-CRITIC-GRA which is a multi-phase methodology developed within the spherical fuzzy environment to evaluate the performance of green gold mines in Egypt. The methodology integrates SWOT analysis for criteria identification, the CRITIC method for objective weighting, and Grey Relational Analysis (GRA) for alternative ranking. Each component operates under the Spherical Fuzzy Set (SFS) framework to effectively handle the uncertainty and hesitation inherent in expert judgments. The proposed methodology consists of three sequential phases. In Phase 1, evaluation criteria are identified using the SF-SWOT approach, which synthesizes expert opinions and literature to capture both internal and external assessment dimensions. Next, in Phase 2 the SF-CRITIC method is applied to calculate the weights of these criteria by considering contrast intensity and inter-criteria correlations. Finally, Phase 3, the SF-GRA technique is applied to rank the gold mine alternatives and determine the most suitable candidate for green conversion. The detailed steps for each phase are presented in the following subsections.

#### Phase 1—SF-SWOT

This initial phase focuses on constructing a comprehensive decision-making structure by identifying and organizing the evaluation criteria using the SWOT framework within a spherical fuzzy environment. It involves collecting expert input and relevant literature to define the main and sub-criteria, which reflect internal strengths and weaknesses as well as external opportunities and threats. These criteria form the basis for assessing the performance of green gold mines. The steps in this phase include defining the problem, selecting evaluation elements, assigning linguistic terms and corresponding spherical fuzzy numbers (SFNs), and generating a structured decision matrix.

**Step 1.** Define the problem and determine all relevant aspects.

At this stage, the decision problem is clearly formulated, and all relevant aspects of green mine performance are systematically identified. The main and sub-criteria are selected based on the SWOT framework and the expert knowledge of the evaluation panel. Table 1 summarizes the background and qualifications of the participating experts who contributed to this stage of the analysis. The weights for each expert were assigned to reflect the domain-specific expertise of the contributors. Experts 1 and 2 were each assigned a weight of 0.25 due to their experience of more than 25 years. The third expert has 20 years of experience and was assigned weight of 0.20. The remaining two experts were assigned weights of 0.15 each as they have experience less than 15 years. This distribution was agreed upon in consultation with the full expert panel to balance methodological knowledge and years of experience, thereby enhancing the quality and consistency of the aggregated evaluations.

**Table 1:** Profile of expert contributors in this study.

Experts	Vocation	Sector	Academic degree	Experience	Weight
Ex_1_	Professor	Industry	Ph.D.	30	0.25
Ex_2_	Dr.	Academia	Ph.D.	25	0.25
Ex_3_	Dr.	Academia	Ph.D.	20	0.20
Ex_4_	Expert	Industry	M.Sc.	12	0.15
Ex_5_	Expert	Industry	M.Sc.	10	0.15

**Step 2.** Collect the main and sub-criteria.

The main and sub-criteria are compiled through an extensive review of prior studies and consultations with domain experts. These criteria encompass dimensions from all four SWOT categories: strengths (STR), opportunities (OPP), threats (THR), and weaknesses (WEA). Each criterion is denoted as $\:{C}_{j}\:$where $\:j=\text{1,2},3\dots\:.n$. The corresponding weight vector is defined as: $\:w=\left({w}_{1},\:{w}_{2},\dots\:.{w}_{n}\right)\:$with the normalization condition $\:\sum\:_{j=1}^{n}{w}_{j}=1.$ The set of decision alternatives are denoted as $\:{A}_{i}=GM{E}_{1},GM{E}_{2},\dots\:GM{E}_{m};i=\text{1,2},\dots\:m$. The evaluations for criteria and alternatives are conducted by a panel of experts $\:E{X}_{e}=E{X}_{1},E{X}_{2},\dots\:E{X}_{k};w$here$\:\:e=\text{1,2},\dots\:k$.

**Step 3.** Define linguistic terms and associated SFNs.

To facilitate expert evaluations under uncertainty, a predefined set of linguistic terms is used to express preferences regarding criteria and alternatives. Each linguistic term is mapped to a corresponding spherical fuzzy number (SFN), as presented in Table 2. These SFNs are later used to construct the decision matrix and perform subsequent aggregation and ranking procedures.

**Step 4.** Construct the decision matrix.

A judgment matrices are created to capture the evaluations of each alternative against each criterion by each expert as shown in Eq. (11). This matrix is initially formed using linguistic terms and subsequently transformed into a numerical matrix by substituting each term with its associated SFN, as shown in Table 2. The resulting matrix from expert $\:k$ is expressed in the form:

11 $$\:{A}_{ij}^{k}=\:\left[\begin{array}{ccc}\left({X}_{{S}_{11}},{Y}_{{S}_{11}},{Z}_{{S}_{11}}\right)&\:\cdots\:&\:\left({X}_{{S}_{1n}},{Y}_{{S}_{1n}},{Z}_{{S}_{1n}}\right)\\\:\vdots&\:\ddots\:&\:\vdots\\\:\left({X}_{{S}_{m1}},{Y}_{{S}_{m1}},{Z}_{{S}_{m1}}\right)&\:\cdots\:&\:\left({X}_{{S}_{mn}},{Y}_{{S}_{mn}},{Z}_{{S}_{mn}}\right)\end{array}\right]$$

**Table 2:** The linguistic terms and their relevant SFNs to evaluate the criteria and alternatives.

Linguistic terms	Abbreviations	SFNs		
		X	Y	Z
Absolutely strong	(AS)	0.9	0.1	0.1
Very strong	(VS)	0.8	0.2	0.2
Strong	(S)	0.7	0.3	0.3
Slightly strong	(SS)	0.6	0.4	0.4
Fair	(F)	0.5	0.5	0.5
Slightly weak	(SW)	0.4	0.6	0.4
Weak	(W)	0.3	0.7	0.3
Very weak	(VW)	0.2	0.8	0.2
Absolutely weak	(AW)	0.1	0.9	0.1

**Step 5.** Aggregate the decision matrix.

After collecting evaluations from all experts, the individual decision matrices are aggregated into a single unified decision matrix. This aggregation is performed using the Weighted Arithmetic Mean (WAM) operator defined in Eq. (6), where the weight assigned to each expert $\:\left({w}_{k}\right)$​ reflects their level of expertise or influence in the decision-making process. The aggregated matrix represents a consensus view of all experts, integrating their assessments into a unified structure.

**Step 6.** Obtain the crisp values.

To facilitate subsequent computational steps, the aggregated spherical fuzzy numbers (SFNs) are converted into crisp scores using the score function defined in Eq. (8). This transformation enables the ranking and comparison of alternatives based on numerical values. The resulting crisp decision matrix is denoted as:

12 $$\:{A}_{ij}=\:\left[\begin{array}{ccc}{A}_{11}&\:\cdots\:&\:{A}_{1n}\\\:\vdots&\:\ddots\:&\:\vdots\\\:{A}_{m1}&\:\cdots\:&\:{A}_{mn}\end{array}\right]$$

Once the aggregated decision matrix is constructed and defuzzified into crisp values, the next step involves determining the relative importance of each criterion. Accurate weighting is essential, as it influences how alternatives are evaluated and compared. To achieve this, the study employs the SF-CRITIC method, which objectively calculates criteria weights by considering both the contrast intensity and inter-criteria correlation within the decision matrix. This phase ensures that the most informative and impactful criteria receive appropriate emphasis in the subsequent ranking process.

#### Phase 2—SF-CRITIC

This phase involves the computation of criteria weights using the CRITIC method adapted to a spherical fuzzy environment. The SF-CRITIC method provides an objective way to assign importance to each criterion by considering both the contrast intensity and the correlation among criteria, thus reflecting their contribution to the overall decision problem. The method includes the following steps.

**Step 1.** Normalize the decision matrix.

As established in Step 6 of Phase 1, the aggregated spherical fuzzy numbers were converted into crisp values using the score function. The resulting crisp decision matrix serves as the basis for the normalization procedure. The decision matrix is normalized to ensure that all criteria are on a comparable scale. Two normalization formulas are used, depending on whether the criterion is a benefit (positive) or a cost (negative) criterion.

For benefit criteria:

13 $$\:{A}_{ij}^{*}=\frac{{A}_{ij}-\text{min}\left({A}_{i}\right)}{\text{max}\left({A}_{i}\right)-\text{min}\left({A}_{i}\right)}$$

For cost criteria:

14 $$\:{A}_{ij}^{*}=1-\frac{{A}_{ij}-\text{max}\left({A}_{i}\right)}{\text{min}\left({A}_{i}\right)-\text{max}\left({A}_{i}\right)}$$

$$\:\text{w}\text{h}\text{e}\text{r}\text{e}\:i=\text{1,2},\dots\:.m\:\text{a}\text{n}\text{d}\:j=\text{1,2},\dots\:,n.$$

Min-max normalization is selected for this study because it maintains the relative performance of alternatives on a uniform [0, 1] scale, which is essential for the SF-CRITIC method’s correlation and variability computations. It effectively accommodates both benefit and cost criteria while preserving interpretability. Alternative methods such as logarithmic normalization are less suitable in this context as they may introduce distortions when values approach zero, and additive methods often lack consistency across diverse criteria scales.

**Step 2.** Determine the correlation coefficient.

The Pearson correlation coefficient between each pair of criteria is calculated to assess intercriteria relationships:

15 $$\:{R}_{jb}=\frac{\sum\:_{i=1}^{m}\left({A}_{ij}^{*}-{A}_{j}^{-}\right)\left({A}_{ib}^{*}-{A}_{b}^{-}\right)}{\sqrt{\sum\:_{i=1}^{m}{\left({A}_{ij}^{*}-{A}_{j}^{-}\right)}^{2}\sum\:_{i=1}^{m}{\left({A}_{ib}^{*}-{A}_{b}^{-}\right)}^{2}}}$$

where $\:{A}_{j}^{-}\:and\:{A}_{b}^{-}$ refer to the $\:{j}^{th}\:and\:{b}^{th}$ alternatives and the mean value $\:{A}_{j}^{-}$ is calculated by:

16 $$\:{A}_{j}^{-}=\frac{1}{n}\sum\:_{j}^{n}{A}_{ij}^{*},\:i=\text{1,2},\dots\:.m$$

**Step 3.** Compute the standard deviation.

The standard deviation of each criterion reflects its variability and is calculated as:

17 $$\:ST{D}_{j}=\:\sqrt{\frac{1}{n-1}{\sum\:_{j=1}^{n}\left({A}_{ij}^{*}-{A}_{j}^{-}\right)}^{2}}i=\text{1,2},\dots\:m$$

**Step 4.** Compute the amount of information.

The information value for each criterion combines its variability and its degree of independence from other criteria:

18 $$\:IN{F}_{j}=ST{D}_{j}\sum\:_{b=1}^{n}\left(1-{R}_{jb}\right);i=\text{1,2},\dots\:.m$$

**Step 5.** Calculate the factors’ weights.

The normalized weights for the criteria are obtained by dividing each information value by the total sum:

19 $$\:{w}_{j}=\frac{IN{F}_{j}}{\sum\:_{j=1}^{n}IN{F}_{j}}$$

**Step 6.** Compute the global weights.

The final global weights for the sub-criteria are determined by multiplying the main criteria weights by their corresponding sub-criteria weights. This step ensures consistency and proportionality across the entire criteria hierarchy.

#### Phase 3—Spherical fuzzy grey relational analysis method (SF-GRA)

After determining the weights of the criteria using the SF-CRITIC method, the next stage involves ranking the gold mine alternatives to identify the most suitable candidate for green conversion. To achieve this, the Spherical Fuzzy Grey Relational Analysis (SF-GRA) method is applied. This technique evaluates the closeness between each alternative and a reference ideal by calculating grey relational coefficients. The method is particularly effective for analyzing systems with uncertain and imprecise information, making it suitable for decision environments involving fuzzy linguistic inputs.

The detailed steps of the SF-GRA method are outlined as follows:

**Step 1.** Normalize the decision matrix using the GRA approach.

The normalized decision matrix is constructed by the GRA method based on the type of criterion using the crisp decision matrix from Eq. (11).

For benefit (positive) criteria:

20 $$\:{O}_{ij}=\frac{{A}_{ij}}{\text{max}\left({A}_{ij}\right)}$$

For cost (negative) criteria:

21 $$\:{O}_{ij}=\frac{\text{min}\left({A}_{ij}\right)}{{A}_{ij}}$$

**Step 2.** Compute the reference series.

The reference series $\:S{R}_{0}$ is derived by identifying the best normalized performance across all alternatives for each criterion.

22 $$\:S{R}_{0}=\left\{S{R}_{01},S{R}_{02},\dots\:S{R}_{0n}\right\}$$

23 $$\:S{R}_{0j}=\underset{i}{\text{max}}{O}_{ij}$$

**Step 3.** Build the distance matrix.

The distance matrix is built between references series and every comparison value as:

24 $$\:{T}_{ij}=\:S{R}_{0j}-{O}_{ij}$$

25 $$\:{T}_{ij}=\left[\begin{array}{ccc}{t}_{11}&\:\cdots\:&\:{t}_{1n}\\\:\vdots &\:\ddots\:&\:\vdots \\\:{t}_{m1}&\:\cdots\:&\:{t}_{mn}\end{array}\right]$$

**Step 4.** Compute the grey relational (GR) coefficient.

Using the distinguishing coefficient $\:\eta\:\in\:\left(\text{0,1}\right)$, the grey relational coefficient $\:{G}_{ij}$​ is computed as:

26 $$\:{G}_{ij}=\frac{{t}_{min}+\eta\:{t}_{max}}{{t}_{ij}+{t}_{max}}$$

where

$$\:{t}_{min}=\text{min}{t}_{ij}$$

$$\:{t}_{max}=\text{max}{t}_{ij}$$.

**Step 5.** Compute the GR grade.

The overall performance of each alternative is evaluated by calculating its relational grade $\:{\beta\:}_{i}$, which aggregates the weighted grey relational coefficients:

27 $$\:{\beta\:}_{i}=\:\sum\:_{j=1}^{n}{w}_{j}{G}_{ij}$$

**Step 6.** Rank the alternatives.

Finally, the alternatives are ranked based on their grey relational grades $\:{\beta\:}_{i}$. The alternative with the highest grade is considered the most suitable for green mine conversion.

The completion of the third phase signifies the full development of the proposed decision-making framework, which integrates criteria identification through SF-SWOT, weight computation via SF-CRITIC, and alternative ranking using SF-GRA. This methodology is subsequently applied in a real-world context. The following section presents the practical implementation of the model for evaluating selected gold mines in Egypt, aiming to determine the most appropriate candidate for green conversion.

## Application

This section presents the application of the proposed methodology within the context of Egypt’s gold mining sector. The case study is first introduced, followed by the application of the SF-CRITIC method for calculating the criteria weights, and subsequently, the application of the SF-GRA method for ranking the alternatives.

### Case study

Egypt plays a significant role in the gold mining industry, with a history dating back thousands of years to the time of the Pharaohs. The country is rich in various natural resources, including gold, due to its strategic location and geological diversity across different regions. Throughout history, Egypt has experienced periods of prosperity in gold mining, with various mines contributing to wealth and trade. In recent times, the gold mining industry in Egypt has seen significant growth, driven by global demand for gold. Numerous companies and investors are now focused on extracting gold from Egypt’s mines, employing advanced technologies and methods to access gold deposits deep within the earth. These companies have also invested in improving mining methods to increase the efficiency of gold production.

The growing gold mining sector in Egypt has brought several benefits, including economic growth, social development, and environmental improvements. Many companies are now turning to sustainable practices, seeking to convert traditional mines into green mines by adopting technological advancements and environmentally friendly methods. Currently, Egypt has more than 100 gold mines. To achieve sustainable development, a large gold mining company in Egypt is looking to convert one of its existing gold mines into a green mine. After a thorough analysis, we identified 20 gold mines in Egypt to assess their performance and select the best one for conversion into a green mine. These 20 mines were selected based on the objectives of this study, which aims to identify the optimal mine for transformation into a green mine.

To ensure the reliability and contextual relevance of the evaluation process, a panel of five domain experts was formed. These experts were selected based on three key criteria: a minimum of ten years of professional experience in the mining industry, academic or technical qualifications related to environmental engineering or mineral economics, and prior involvement in sustainability-oriented mining assessments. All experts participated in structured interviews and provided linguistic evaluations of the criteria and alternatives using the predefined spherical fuzzy linguistic scale. In the aggregation step, each expert’s opinion was converted into spherical fuzzy numbers and then integrated to form a collective decision matrix.

### The results of the SF-CRITIC method

This section presents the outcomes derived from the application of the SF-CRITIC method to compute the weights of both main and sub-criteria for evaluating green gold mines.

**Step 1**: The problem is clearly defined, and the relevant details are established. The goal of this study is to assess the performance of gold green mines in Egypt and to select the best one for conversion into a green mine. Five experts were invited to contribute their opinions, as outlined in Table 1.

**Step 2**: Four main criteria were analyzed using the SWOT method: strengths, weaknesses, opportunities, and threats. Additionally, 37 sub-criteria were identified, and 20 gold mines (alternatives) were selected for evaluation.

**Step 3.** There are 9 linguistic terms and 9 SFNs in Table 2 that are used by the experts to evaluate the criteria and alternatives. For instance, slightly strong (SS) has a (0.6,0.4,0.4), where these SFNs refer to the membership and non-membership and hesitancy degrees respectively. The experts used SFNs to compute the weights of key and sub-factors and rank the alternatives. The alternatives in this study denoted as: $\:GM{E}_{1},GM{E}_{2}\dots\:.GM{E}_{m}$.

**Step 4.** A decision matrix was constructed using Eq. (11), in which experts evaluated each criterion-alternative pair using the linguistic terms from Table 2. These terms were then converted into their corresponding SFNs.

**Step 5.** The individual expert matrices were aggregated into a single judgment matrix using the Weighted Arithmetic Mean (WAM) method, as defined in Eq. (6). The aggregated matrix is provided in Table 3.

**Step 6.** Crisp values were derived from the aggregated SFNs by applying the score function described in Eq. (8). The resulting crisp decision matrix is displayed in Table 4.

#### Results of SWOT main criteria

The SF-CRITIC approach is continued to compute the weights of the main SWOT criteria through the following steps. Following the aggregation and defuzzification of expert evaluations, the decision matrix is normalized, and the contrast intensity and inter-criteria correlations are analyzed. The results are presented through a series of computed matrices and summarized weights.

**Table 3:** The aggregated decision matrix.

	STR	OPP	WEA	THR
GME_1_	(0.610, 0.460, 0.209)	(0.629, 0.414, 0.243)	(0.889, 0.111, 0.114)	(0.610, 0.460, 0.209)
GME_2_	(0.750, 0.264, 0.207)	(0.862, 0.141, 0.165)	(0.732, 0.291, 0.201)	(0.750, 0.264, 0.207)
GME_3_	(0.710, 0.309, 0.280)	(0.637, 0.370, 0.358)	(0.631, 0.379, 0.349)	(0.710, 0.309, 0.280)
GME_4_	(0.518, 0.500, 0.373)	(0.356, 0.650, 0.361)	(0.843, 0.168, 0.131)	(0.518, 0.500, 0.373)
GME_5_	(0.885, 0.115, 0.118)	(0.655, 0.348, 0.358)	(0.456, 0.578, 0.340)	(0.885, 0.115, 0.118)
GME_6_	(0.838, 0.162, 0.167)	(0.456, 0.580, 0.282)	(0.732, 0.291, 0.201)	(0.838, 0.162, 0.167)
GME_7_	(0.570, 0.435, 0.393)	(0.728, 0.285, 0.239)	(0.435, 0.591, 0.305)	(0.570, 0.435, 0.393)
GME_8_	(0.777, 0.263, 0.126)	(0.530, 0.523, 0.257)	(0.603, 0.414, 0.350)	(0.777, 0.263, 0.126)
GME_9_	(0.538, 0.472, 0.380)	(0.373, 0.659, 0.303)	(0.688, 0.343, 0.229)	(0.538, 0.472, 0.380)
GME_10_	(0.820, 0.180, 0.183)	(0.776, 0.238, 0.188)	(0.568, 0.453, 0.332)	(0.820, 0.180, 0.183)
GME_11_	(0.683, 0.325, 0.345)	(0.603, 0.426, 0.327)	(0.353, 0.664, 0.361)	(0.683, 0.325, 0.345)
GME_12_	(0.885, 0.115, 0.118)	(0.766, 0.241, 0.213)	(0.318, 0.684, 0.319)	(0.885, 0.115, 0.118)
GME_13_	(0.771, 0.232, 0.249)	(0.393, 0.702, 0.165)	(0.800, 0.200, 0.200)	(0.771, 0.232, 0.249)
GME_14_	(0.338, 0.680, 0.346)	(0.384, 0.677, 0.231)	(0.498, 0.533, 0.361)	(0.338, 0.680, 0.346)
GME_15_	(0.655, 0.348, 0.358)	(0.556, 0.460, 0.367)	(0.464, 0.542, 0.402)	(0.655, 0.348, 0.358)
GME_16_	(0.821, 0.189, 0.192)	(0.200, 0.827, 0.210)	(0.737, 0.287, 0.200)	(0.821, 0.189, 0.192)
GME_17_	(0.660, 0.384, 0.203)	(0.784, 0.217, 0.219)	(0.490, 0.522, 0.378)	(0.660, 0.384, 0.203)
GME_18_	(0.531, 0.493, 0.341)	(0.541, 0.500, 0.292)	(0.600, 0.400, 0.400)	(0.531, 0.493, 0.341)
GME_19_	(0.618, 0.383, 0.385)	(0.490, 0.522, 0.378)	(0.800, 0.200, 0.200)	(0.618, 0.383, 0.385)
GME_20_	(0.801, 0.227, 0.152)	(0.715, 0.310, 0.218)	(0.853, 0.148, 0.159)	(0.801, 0.227, 0.152)

**Table 4:** The crisp decision matrix.

	STR	OPP	WEA	THR
GME_1_	0.129686	0.632927	0.171174	0.690201
GME_2_	0.391879	0.028065	0.603405	0.363036
GME_3_	0.296773	0.512203	0.173421	0.167064
GME_4_	0.011777	0.200577	0.190001	0.594075
GME_5_	0.679870	0.232078	0.198482	0.084751
GME_6_	0.563475	0.250397	0.093349	0.363036
GME_7_	0.082854	0.415822	0.343155	0.112147
GME_8_	0.469436	0.162011	0.005467	0.125885
GME_9_	0.041804	0.236092	0.208579	0.276679
GME_10_	0.523178	0.085044	0.444201	0.078882
GME_11_	0.237423	0.436862	0.124125	0.204008
GME_12_	0.679870	0.083309	0.416530	0.250060
GME_13_	0.406902	0.252895	0.286603	0.480000
GME_14_	0.229475	0.374916	0.243228	0.023573
GME_15_	0.198482	0.004670	0.062272	0.047282
GME_16_	0.517335	0.321559	0.512803	0.370504
GME_17_	0.232204	0.038605	0.442714	0.020711
GME_18_	0.026300	0.293970	0.030980	0.120000
GME_19_	0.144421	0.152533	0.020711	0.480000
GME_20_	0.502547	0.050462	0.327292	0.594156

**Step 7.** The judgment matrix between the main SWOT criteria and the alternatives is normalized using Eq. (13). The normalized decision matrix is displayed in Table 5.

**Step 8.** The correlation coefficients between criteria are calculated using Eqs. (15) and (16). These coefficients reflect the degree of redundancy between criteria and are shown in Table 6.

**Step 9.** The standard deviation for each criterion is computed using Eq. (17). These values represent the contrast intensity across alternatives and are included in Table 6.

**Step 10.** The amount of information conveyed by each criterion is calculated using Eq. (18), incorporating both contrast intensity and correlation. The results are shown in Table 6.

**Fig. 2:**
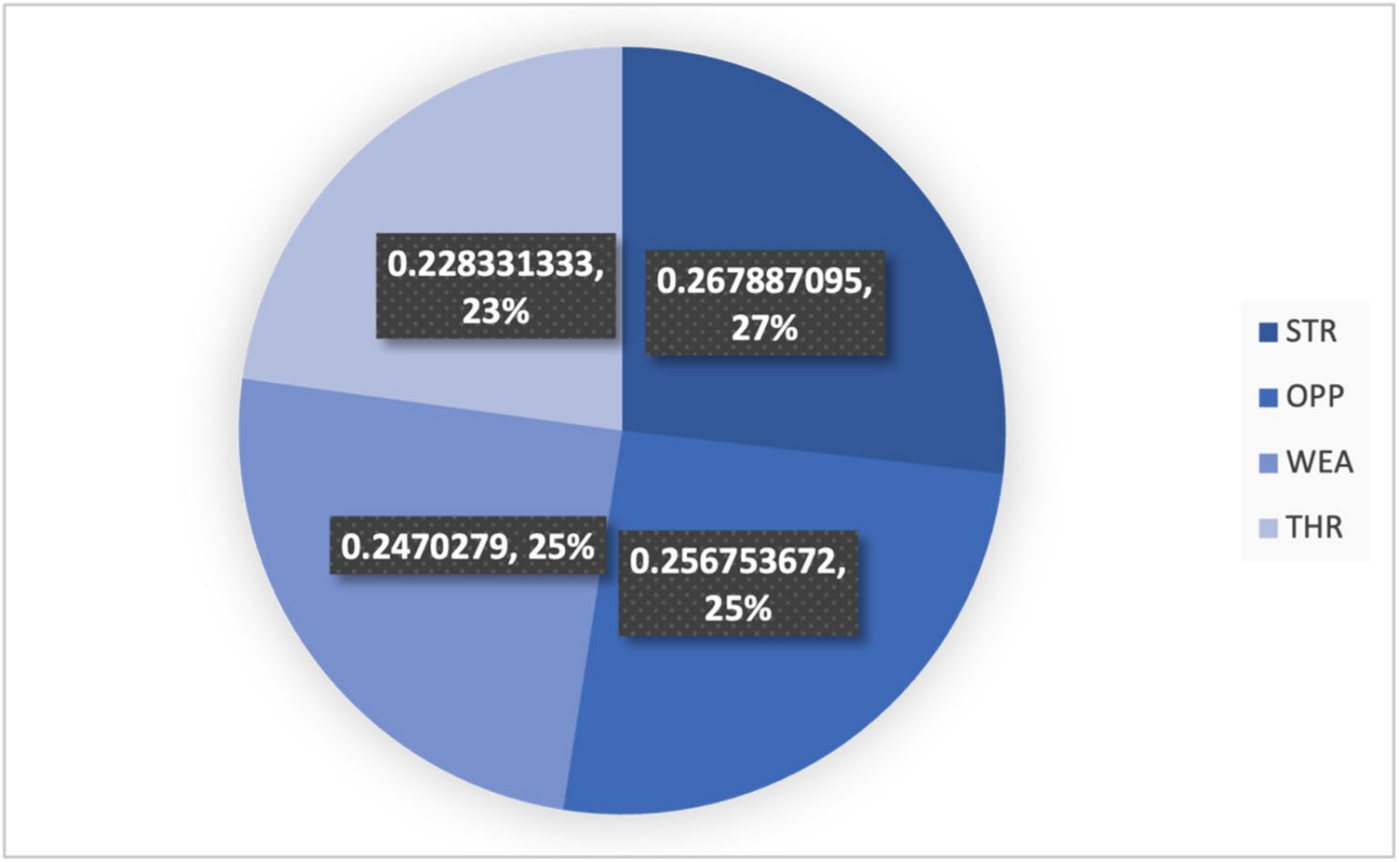
The weights of main criteria by the CRITIC method.

**Step 11.** The final weights of the main criteria are computed using Eq. (19) and visualized in Fig. 2. The STR criterion is assigned the highest weight (0.267), followed by OPP (0.257), THR (0.247), and WEA (0.228). This indicates that strengths are the most significant factor in evaluating the green transformation potential of gold mines in Egypt, emphasizing the importance of internal capabilities and performance enablers.

These steps allow for the objective determination of each criterion’s importance in assessing the performance of green gold mines.

**Table 5:** The normalized decision matrix using the CRITIC method.

	STR	OPP	WEA	THR
GME_1_	0.176487	1.000000	0.277131	1.000000
GME_2_	0.568936	0.037238	1.000000	0.511321
GME_3_	0.426582	0.807843	0.280889	0.218604
GME_4_	0.000000	0.311827	0.308617	0.856419
GME_5_	1.000000	0.361968	0.322801	0.095654
GME_6_	0.825780	0.391125	0.146974	0.511321
GME_7_	0.106388	0.654434	0.564753	0.136576
GME_8_	0.685023	0.250441	0.000000	0.157095
GME_9_	0.044945	0.368355	0.339687	0.382332
GME_10_	0.765463	0.127932	0.733745	0.086888
GME_11_	0.337747	0.687923	0.198446	0.273786
GME_12_	1.000000	0.125170	0.687467	0.342573
GME_13_	0.591422	0.395101	0.470175	0.686028
GME_14_	0.325850	0.589322	0.397634	0.004275
GME_15_	0.279460	0.000000	0.095002	0.039688
GME_16_	0.756717	0.504394	0.848476	0.522476
GME_17_	0.329935	0.054015	0.731258	0.000000
GME_18_	0.021738	0.460481	0.042669	0.148305
GME_19_	0.198542	0.235354	0.025495	0.686028
GME_20_	0.734583	0.072887	0.538225	0.856539

**Table 6:** The correlation matrix for the weights of the main criteria.

	STR	OPP	THR	WEA	$\:ST{D}_{j}$	$\:{W}_{j}$
STR	1.000000	– 0.317020	0.347624	– 0.076430	0.323369	0.267887
OPP	– 0.317020	1.000000	– 0.266380	0.149796	0.274927	0.256754
THR	0.347624	– 0.266380	1.000000	0.015770	0.289182	0.247028
WEA	– 0.076430	0.149796	0.015770	1.000000	0.312015	0.228331

#### Results of strength sub-criteria

The analysis proceeds by examining the sub-criteria associated with the Strength (STR) dimension of the SWOT framework. These steps aim to compute the individual weights of each strength-related factor to better understand their relative influence on the evaluation of green gold mines in Egypt. The SF-CRITIC method is employed to process expert input through aggregation, defuzzification, normalization, and weighting. The following steps describe the detailed computations and results.

**Step 12.** The decision matrix between strength sub-criteria and alternatives is constructed using Eq. (11). Five experts express their preferences using linguistic terms as listed in Table 1. These terms are then replaced with their corresponding Spherical Fuzzy Numbers (SFNs).

**Step 13.** The individual expert matrices are aggregated into a single decision matrix using the Weighted Arithmetic Mean (WAM), as defined in Eq. (6).

**Step 14.** Crisp values corresponding to each SFN are computed by applying the score function in Eq. (8). The resulting values are shown in Table 7.

**Step 15.** The normalized matrix for strength sub-criteria is calculated using Eq. (13), and the results are displayed in Table 8.

**Step 16.** Correlation coefficients between the strength sub-criteria are determined using Eqs. (15) and (16). These coefficients, which reflect the interdependence between criteria, are reported in Table 9.

**Step 17.** The standard deviation for each sub-criterion is computed using Eq. (17) to capture the level of contrast across the alternatives. These values are included in Table 9.

**Step 18.** The amount of information for each sub-criterion is calculated using Eq. (18), integrating both variability and correlation, as shown in Table 9.

**Fig. 3:**
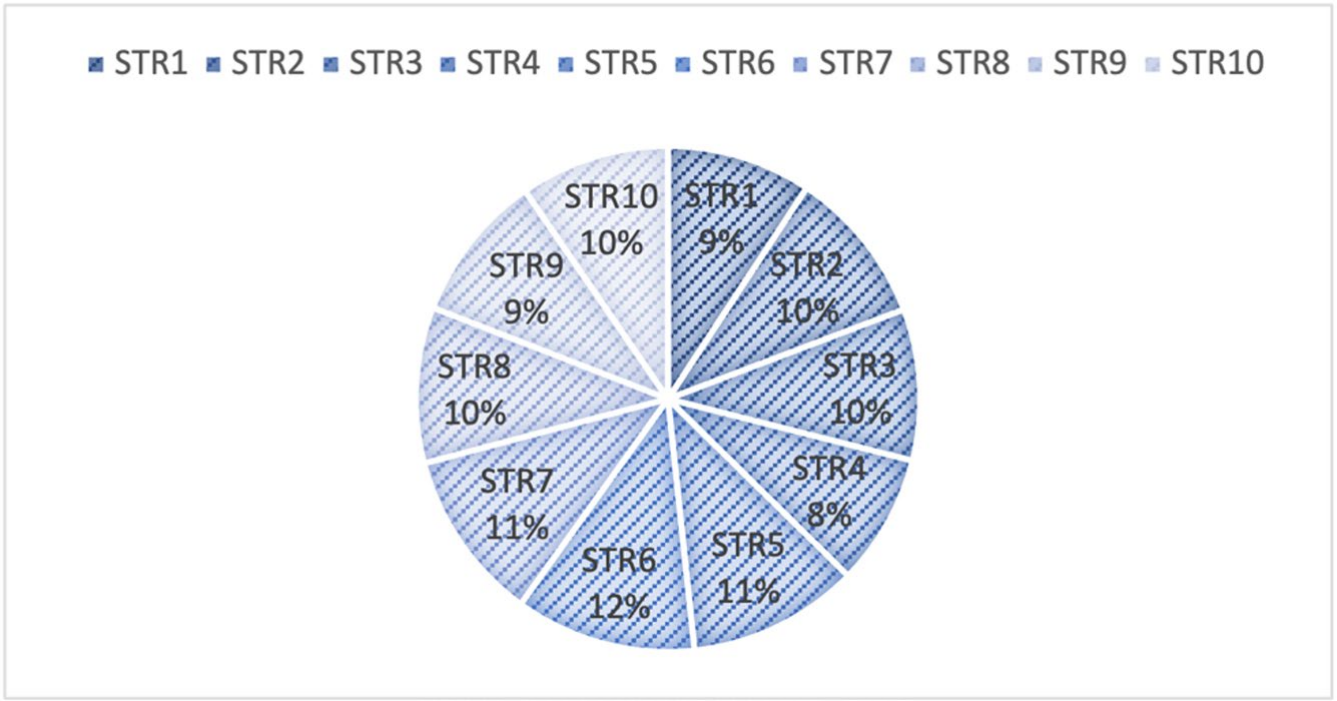
The weights of strength sub-criteria.

**Step 19.** The final weights of the strength sub-criteria are computed using Eq. (19) and are visualized in Fig. 3.

These computed weights provide insight into the relative importance of internal strengths in the context of green mine evaluation. By quantifying expert judgments, this phase facilitates more informed decision-making and contributes to a structured assessment of the strategic capabilities of each alternative.

**Table 7:** The crisp values between strength sub-criteria and alternatives.

	STR_1_	STR_2_	STR_3_	STR_4_	STR_5_	STR_6_	STR_7_	STR_8_	STR_9_	STR_10_
GME_1_	0.392483	0.720000	0.413980	0.720000	0.172526	0.653371	0.523178	0.413980	0.309929	0.363459
GME_2_	0.421441	0.120000	0.605522	0.343425	0.413980	0.460332	0.093349	0.444300	0.005467	0.421441
GME_3_	0.335027	0.720000	0.198755	0.275479	0.162509	0.286451	0.028097	0.093349	0.179157	0.271511
GME_4_	0.190559	0.343425	0.146802	0.627793	0.413718	0.327000	0.345467	0.117656	0.280000	0.124745
GME_5_	0.621232	0.177894	0.069785	0.140028	0.280340	0.070349	0.035819	0.421441	0.653371	0.341373
GME_6_	0.416530	0.064199	0.007463	0.341484	0.188004	0.202462	0.394496	0.489482	0.523178	0.030340
GME_7_	0.144421	0.332244	0.294597	0.011161	0.593466	0.183929	0.339513	0.161025	0.080133	0.720000
GME_8_	0.646541	0.038463	0.182693	0.198755	0.026300	0.158874	0.659652	0.198482	0.157182	0.415249
GME_9_	0.120000	0.043134	0.017031	0.357449	0.368135	0.617828	0.269094	0.207479	0.202346	0.075589
GME_10_	0.480000	0.237023	0.233362	0.174138	0.326437	0.139069	0.029259	0.164957	0.030837	0.657510
GME_11_	0.120000	0.337314	0.197887	0.062481	0.120000	0.619024	0.518280	0.501397	0.391236	0.061039
GME_12_	0.679870	0.062858	0.284248	0.280000	0.600261	0.720000	0.060311	0.277706	0.194470	0.421441
GME_13_	0.393016	0.238749	0.315941	0.420096	0.193484	0.523178	0.552766	0.147874	0.412461	0.061244
GME_14_	0.020711	0.406902	0.058112	0.179157	0.480000	0.069785	0.416530	0.069785	0.426092	0.047479
GME_15_	0.237423	0.269890	0.100721	0.139069	0.120000	0.657510	0.720000	0.284248	0.280673	0.275479
GME_16_	0.516001	0.209041	0.005505	0.523178	0.679870	0.068000	0.480000	0.237423	0.494023	0.393016
GME_17_	0.144711	0.180062	0.252685	0.000857	0.050695	0.460332	0.082854	0.346920	0.035229	0.079498
GME_18_	0.102320	0.539479	0.137241	0.082854	0.167064	0.286451	0.690201	0.355757	0.627793	0.120000
GME_19_	0.020360	0.069785	0.120000	0.480000	0.627793	0.541566	0.139069	0.311276	0.020711	0.280000
GME_20_	0.720000	0.075589	0.480000	0.720000	0.088975	0.070349	0.720000	0.152533	0.720000	0.720000

**Table 8:** The normalized decision matrix between strength sub-criteria and alternatives by the CRITIC method.

	STR_1_	STR_2_	STR_3_	STR_4_	STR_5_	STR_6_	STR_7_	STR_8_	STR_9_	STR_10_
GME_1_	0.531877	1.000000	0.680772	1.000000	0.223734	0.897809	0.715535	0.797464	0.426099	0.483020
GME_2_	0.573267	0.119636	1.000000	0.476355	0.593173	0.601736	0.094308	0.867712	0.000000	0.567092
GME_3_	0.449755	1.000000	0.322075	0.381874	0.208408	0.335047	0.000000	0.054594	0.243083	0.349696
GME_4_	0.243267	0.447461	0.235489	0.871783	0.592772	0.397240	0.458692	0.110912	0.384213	0.136887
GME_5_	0.858831	0.204583	0.107131	0.193523	0.388696	0.003603	0.011161	0.814750	0.906752	0.450995
GME_6_	0.566248	0.037762	0.003264	0.473657	0.247417	0.206230	0.529553	0.972395	0.724544	0.000000
GME_7_	0.177322	0.431056	0.481807	0.014328	0.867796	0.177805	0.450086	0.211393	0.104496	1.000000
GME_8_	0.895004	0.000000	0.295306	0.275186	0.000000	0.139378	0.912779	0.298177	0.212327	0.558114
GME_9_	0.142416	0.006853	0.019210	0.495856	0.523027	0.843294	0.348310	0.319023	0.275535	0.065610
GME_10_	0.656966	0.291341	0.379752	0.240954	0.459227	0.109003	0.001680	0.220503	0.035506	0.909390
GME_11_	0.142416	0.438495	0.320627	0.085691	0.143367	0.845128	0.708457	1.000000	0.539889	0.044514
GME_12_	0.942642	0.035794	0.464558	0.388160	0.878193	1.000000	0.046559	0.481731	0.264513	0.567092
GME_13_	0.532639	0.293874	0.517378	0.582970	0.255802	0.698125	0.758299	0.180924	0.569595	0.044811
GME_14_	0.000502	0.540600	0.087677	0.247934	0.694187	0.002739	0.561398	0.000000	0.588672	0.024852
GME_15_	0.310250	0.339566	0.158690	0.192190	0.143367	0.904157	1.000000	0.496887	0.385155	0.355450
GME_16_	0.708423	0.250283	0.000000	0.726310	1.000000	0.000000	0.653131	0.388400	0.683742	0.525876
GME_17_	0.177735	0.207763	0.411954	0.000000	0.037327	0.601736	0.079140	0.642092	0.041652	0.071279
GME_18_	0.117146	0.735126	0.219555	0.114020	0.215377	0.335047	0.956932	0.662567	0.870956	0.130006
GME_19_	0.000000	0.045958	0.190820	0.666269	0.920319	0.726328	0.160387	0.559509	0.021335	0.362005
GME_20_	1.000000	0.054473	0.790803	1.000000	0.095896	0.003603	1.000000	0.191717	1.000000	1.000000

**Table 9:** The correlation matrix between strength sub-criteria and weights of criteria.

	STR_1_	STR_2_	STR_3_	STR_4_	STR_5_	STR_6_	STR_7_	STR_8_	STR_9_	STR_10_	$\:ST{D}_{j}$	$\:{W}_{j}$
STR_1_	1.0000	– 0.3139	0.3130	0.3038	– 0.1362	– 0.2681	– 0.0056	0.0206	0.2595	0.5368	0.322612	0.092756
STR_2_	– 0.3139	1.0000	0.0716	0.0047	– 0.1767	0.0340	0.1022	– 0.1135	0.0923	– 0.1207	0.304993	0.099644
STR_3_	– 0.4045	1.0000	– 0.1075	– 0.2952	– 0.1173	– 0.1530	0.0231	– 0.2960	0.1026	– 0.1867	0.267660	0.096864
STR_4_	0.3038	0.0047	0.2450	1.0000	0.1417	0.0437	0.1923	– 0.1458	0.2193	0.1334	0.308166	0.084029
STR_5_	– 0.1362	– 0.1767	– 0.1593	0.1417	1.0000	– 0.0529	– 0.3852	– 0.2032	– 0.2389	0.2139	0.316875	0.109864
STR_6_	– 0.2681	0.0340	0.1954	0.0437	– 0.0529	1.0000	– 0.0450	0.3333	– 0.3793	– 0.3239	0.353863	0.116135
STR_7_	– 0.0056	0.1022	– 0.0322	0.1923	– 0.3852	– 0.0450	1.0000	– 0.0408	0.5283	– 0.0755	0.360972	0.109687
STR_8_	0.0206	– 0.1135	0.0692	– 0.1458	– 0.2032	0.3333	– 0.0408	1.0000	0.1172	– 0.2245	0.313202	0.099800
STR_9_	0.2595	0.0923	– 0.2336	0.2193	– 0.2389	– 0.3793	0.5283	0.1172	1.0000	– 0.1583	0.312150	0.095199
STR_10_	0.5368	– 0.1207	0.4866	0.1334	0.2139	– 0.3239	– 0.0755	– 0.2245	– 0.1583	1.0000	0.324495	0.096023

#### Results of opportunities sub-criteria

The analysis proceeds to evaluate the opportunities sub-criteria using the SF-CRITIC method. The following steps outline the calculation and processing of expert evaluations to determine the relative weights of these sub-criteria.

**Step 20.** The decision matrix between the opportunities sub-criteria and alternatives is constructed based on expert evaluations using linguistic terms, in accordance with Eq. (11). These linguistic terms are then replaced with their corresponding spherical fuzzy numbers (SFNs).

**Step 21.** The decision matrices provided by the five experts are aggregated into a single matrix using the Weighted Arithmetic Mean (WAM), as defined in Eq. (6).

**Step 22.** Crisp values are obtained by applying the score function in Eq. (8). The resulting matrix is shown in Table 10.

**Step 23.** The judgment matrix is normalized using Eq. (13) to standardize the data across all alternatives. The normalized values are presented in Table 11.

**Step 24.** Correlation coefficients among the opportunities sub-criteria are computed using Eqs. (15) and (16). These coefficients indicate the relationships and redundancies between the criteria, as displayed in Table 12.

**Step 25.** The standard deviations of each sub-criterion are calculated using Eq. (17) to reflect the contrast intensity across alternatives.

**Step 26.** The amount of information conveyed by each sub-criterion is determined using Eq. (18), which considers both contrast intensity and inter-criteria correlation.

**Fig. 4:**
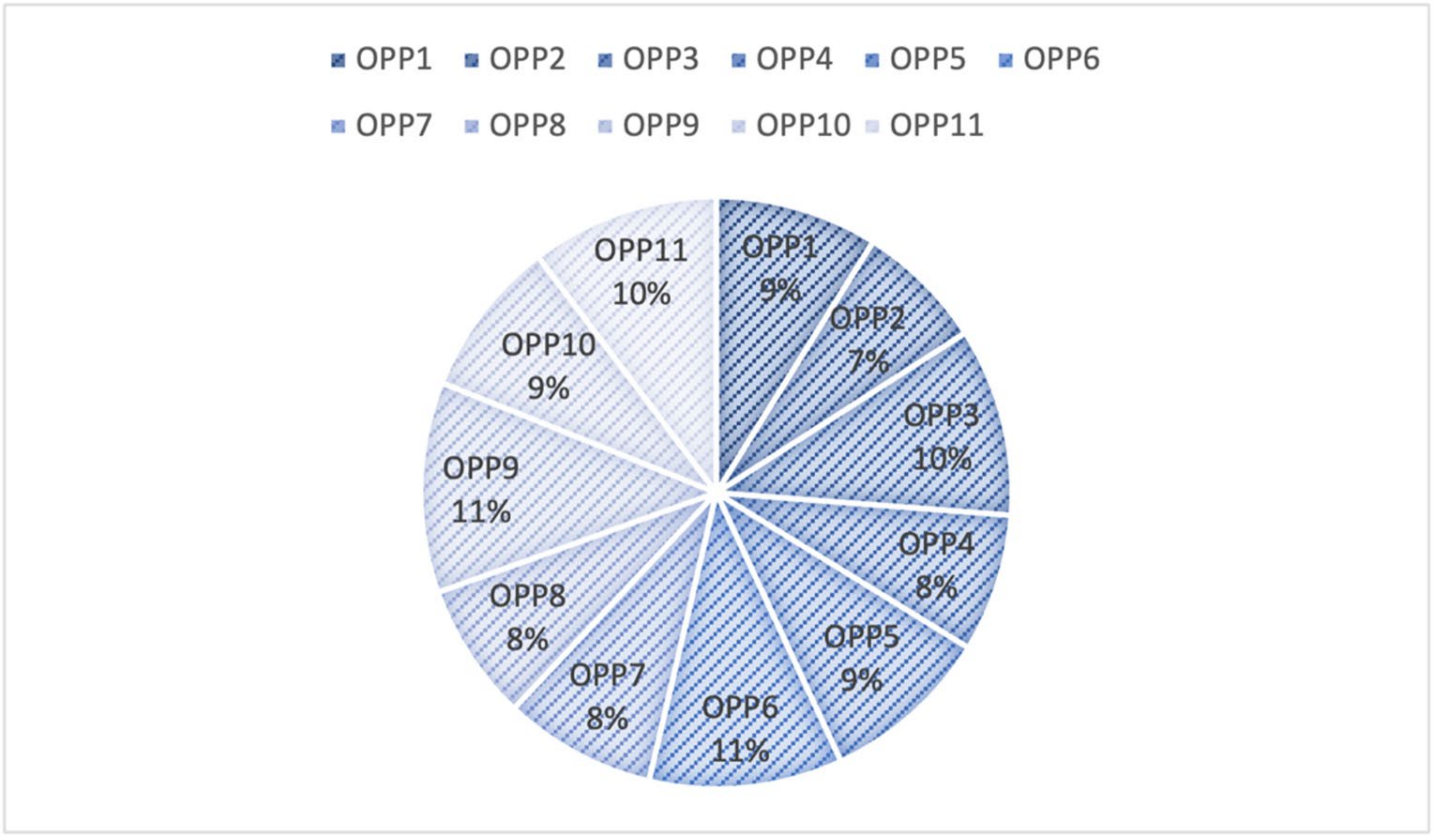
The weights of opportunities sub-criteria.

**Step 27.** The final weights of the opportunities sub-criteria are computed using Eq. (19). These weights are illustrated in Fig. 4.

This process enables a structured evaluation of external opportunity factors, highlighting their respective importance in the green mine context and supporting a comprehensive multi-criteria decision-making framework.

**Table 10:** The crisp values between opportunities sub-criteria and alternatives.

	OPP_1_	OPP_2_	OPP_3_	OPP_4_	OPP_5_	OPP_6_	OPP_7_	OPP_8_	OPP_9_	OPP_10_	OPP_11_
GME_1_	0.603405	0.720000	0.397100	0.591128	0.563693	0.603405	0.397100	0.523178	0.078897	0.567664	0.133346
GME_2_	0.010987	0.291941	0.405777	0.258187	0.355481	0.433200	0.266696	0.214947	0.062481	0.276754	0.178567
GME_3_	0.120000	0.407865	0.008045	0.056967	0.178567	0.071396	0.074151	0.013241	0.035708	0.154748	0.098337
GME_4_	0.067200	0.161075	0.310996	0.305494	0.115743	0.636330	0.034000	0.246227	0.269045	0.027741	0.144799
GME_5_	0.361897	0.018692	0.081614	0.027520	0.091197	0.047516	0.106140	0.027520	0.613734	0.020570	0.163926
GME_6_	0.442714	0.334851	0.125676	0.155431	0.081100	0.657510	0.279249	0.487907	0.301547	0.444300	0.362710
GME_7_	0.120000	0.319024	0.264984	0.120000	0.240659	0.430698	0.197423	0.280000	0.049125	0.550952	0.541334
GME_8_	0.020711	0.168912	0.119086	0.135478	0.406902	0.120000	0.357449	0.214277	0.291941	0.292517	0.573872
GME_9_	0.160698	0.073575	0.118541	0.397100	0.407805	0.720000	0.397100	0.307717	0.276754	0.084790	0.553737
GME_10_	0.392969	0.154748	0.237117	0.002503	0.245840	0.120000	0.242733	0.094982	0.018999	0.073060	0.430698
GME_11_	0.065417	0.525585	0.376885	0.047282	0.456539	0.720000	0.379681	0.216818	0.525585	0.156986	0.045922
GME_12_	0.501001	0.141704	0.258187	0.068195	0.532819	0.720000	0.108352	0.404170	0.010987	0.442714	0.372197
GME_13_	0.254615	0.120000	0.132957	0.283440	0.020711	0.397100	0.651549	0.231524	0.495807	0.084280	0.657510
GME_14_	0.044591	0.430698	0.025057	0.182526	0.430698	0.109357	0.452149	0.217595	0.430698	0.081614	0.320929
GME_15_	0.297649	0.009461	0.055266	0.075182	0.133346	0.051279	0.534852	0.078897	0.107343	0.159811	0.051279
GME_16_	0.139069	0.200686	0.315890	0.444300	0.078941	0.000101	0.183930	0.139069	0.020711	0.227341	0.063709
GME_17_	0.155431	0.092216	0.034704	0.100088	0.223683	0.310169	0.254615	0.049561	0.232590	0.250414	0.141789
GME_18_	0.132696	0.039563	0.113450	0.167536	0.249204	0.071396	0.075182	0.314113	0.280065	0.316346	0.249204
GME_19_	0.361897	0.149706	0.195901	0.057532	0.428713	0.613734	0.193928	0.452273	0.373656	0.076678	0.021965
GME_20_	0.651421	0.452149	0.361897	0.401849	0.075182	0.044591	0.480000	0.081614	0.651549	0.448314	0.448314

**Table 11:** The normalized decision matrix between opportunities sub-criteria and alternatives by the CRITIC method.

	OPP_1_	OPP_2_	OPP_3_	OPP_4_	OPP_5_	OPP_6_	OPP_7_	OPP_8_	OPP_9_	OPP_10_	OPP_11_
GME_1_	0.925026	1.000000	0.978182	1.000000	1.000000	0.838040	0.587969	1.000000	0.106017	1.000000	0.175252
GME_2_	0.000000	0.397558	1.000000	0.434376	0.616541	0.601611	0.376805	0.395551	0.080389	0.468264	0.246406
GME_3_	0.170218	0.560708	0.000000	0.092528	0.290721	0.099035	0.065017	0.000000	0.038593	0.245256	0.120167
GME_4_	0.087773	0.213379	0.761697	0.514744	0.175018	0.883775	0.000000	0.456893	0.402863	0.013108	0.193274
GME_5_	0.547925	0.012991	0.184971	0.042502	0.129814	0.065863	0.116816	0.028002	0.940967	0.000000	0.223369
GME_6_	0.674116	0.457948	0.295754	0.259806	0.111217	0.913196	0.397132	0.930832	0.453603	0.774510	0.536146
GME_7_	0.170218	0.435674	0.646010	0.199613	0.405075	0.598135	0.264632	0.523122	0.059539	0.969453	0.817203
GME_8_	0.015184	0.224408	0.279186	0.225908	0.711241	0.166550	0.523762	0.394238	0.438607	0.497077	0.868400
GME_9_	0.233764	0.090233	0.277816	0.670371	0.712905	1.000000	0.587969	0.577476	0.414897	0.117384	0.836718
GME_10_	0.596443	0.204475	0.575946	0.000000	0.414617	0.166550	0.338002	0.160297	0.012509	0.095944	0.643121
GME_11_	0.084989	0.726384	0.927359	0.076075	0.802657	1.000000	0.559763	0.399220	0.803355	0.249347	0.037695
GME_12_	0.765128	0.186117	0.628921	0.111604	0.943140	1.000000	0.120398	0.766622	0.000000	0.771612	0.551073
GME_13_	0.380410	0.155571	0.314060	0.477276	0.000000	0.551464	1.000000	0.428060	0.756867	0.116452	1.000000
GME_14_	0.052471	0.592841	0.042772	0.305837	0.755065	0.151766	0.677111	0.400745	0.655223	0.111579	0.470405
GME_15_	0.447606	0.000000	0.118726	0.123474	0.207437	0.071090	0.811033	0.128754	0.150425	0.254511	0.046123
GME_16_	0.199993	0.269127	0.774000	0.750558	0.107242	0.000000	0.242782	0.246753	0.015181	0.377944	0.065682
GME_17_	0.225540	0.116468	0.067027	0.165785	0.373810	0.430710	0.357242	0.071225	0.345951	0.420117	0.188538
GME_18_	0.190042	0.042366	0.265016	0.280372	0.420812	0.099035	0.066686	0.590019	0.420067	0.540632	0.357550
GME_19_	0.547925	0.197378	0.472319	0.093488	0.751410	0.852388	0.258972	0.860953	0.566173	0.102557	0.000000
GME_20_	1.000000	0.623031	0.889673	0.678439	0.100318	0.061800	0.722210	0.134082	1.000000	0.781848	0.670840

**Table 12:** The correlation matrix between opportunities sub-criteria and weights of criteria.

	OPP_1_	OPP_2_	OPP_3_	OPP_4_	OPP_5_	OPP_6_	OPP_7_	OPP_8_	OPP_9_	OPP_10_	OPP_11_	$\:ST{D}_{j}$	$\:{W}_{j}$
OPP_1_	1.0000	0.1995	0.2191	0.1963	– 0.0290	0.0941	0.1568	0.2723	0.1113	0.3989	0.0629	0.305990	0.088913
OPP_2_	0.1995	1.0000	0.4807	0.3870	0.3496	0.2077	0.2030	0.2704	0.0147	0.4700	– 0.1196	0.266952	0.070287
OPP_3_	– 0.0777	1.0000	0.3583	0.0685	0.1413	0.0974	0.1373	0.0010	0.1708	0.2852	– 0.0247	0.333199	0.102929
OPP_4_	0.1963	0.3870	0.4638	1.0000	0.0150	0.1251	0.2919	0.2945	– 0.0194	0.3088	0.1031	0.276326	0.075619
OPP_5_	– 0.0290	0.3496	0.2287	0.0150	1.0000	0.4648	– 0.0065	0.5095	– 0.2123	0.2077	– 0.0673	0.311898	0.093047
OPP_6_	0.0941	0.2077	0.3718	0.1251	0.4648	1.0000	– 0.0259	0.7164	– 0.0397	0.1598	0.0384	0.384122	0.105842
OPP_7_	0.1568	0.2030	– 0.0232	0.2919	– 0.0065	– 0.0259	1.0000	– 0.0103	0.3447	0.0083	0.3670	0.274497	0.083371
OPP_8_	0.2723	0.2704	0.2995	0.2945	0.5095	0.7164	– 0.0103	1.0000	– 0.1157	0.4375	0.1083	0.298688	0.075311
OPP_9_	0.1113	0.0147	– 0.1094	– 0.0194	– 0.2123	– 0.0397	0.3447	– 0.1157	1.0000	– 0.2925	0.1357	0.323895	0.115215
OPP_10_	0.3989	0.4700	0.3844	0.3088	0.2077	0.1598	0.0083	0.4375	– 0.2925	1.0000	0.1948	0.319921	0.086305
OPP_11_	0.0629	– 0.1196	– 0.1033	0.1031	– 0.0673	0.0384	0.3670	0.1083	0.1357	0.1948	1.0000	0.318216	0.103161

#### Results of threats sub-criteria

The SF-CRITIC methodology is employed to calculate the weights of the threat-related sub-criteria based on expert assessments. The following steps describe the procedure for aggregating expert opinions and deriving the importance of each sub-factor.

**Step 28.** The decision matrix for threats sub-criteria and alternatives is constructed using Eq. (11), incorporating expert evaluations based on linguistic terms listed in Table 1. These terms are converted into their corresponding spherical fuzzy numbers (SFNs).

**Step 29.** The individual expert matrices are aggregated using the Weighted Arithmetic Mean (WAM) as defined in Eq. (6), producing a unified decision matrix.

**Step 30.** Crisp values for each alternative with respect to the threats sub-criteria are computed using the score function in Eq. (8). The resulting crisp matrix is presented in Table 13.

**Step 31.** The judgment matrix is normalized using Eq. (13) to standardize evaluation scales across all sub-criteria. The normalized matrix is shown in Table 14.

**Step 32.** Correlation coefficients between the threat sub-criteria are calculated using Eqs. (15) and (16), as shown in Table 15. These values indicate the level of interdependence among the sub-criteria.

**Step 33.** The standard deviations for each sub-criterion are computed using Eq. (17), reflecting the contrast intensity across alternatives.

**Step 34.** The amount of information conveyed by each sub-criterion is evaluated using Eq. (18), incorporating both contrast and correlation values.

**Fig. 5:**
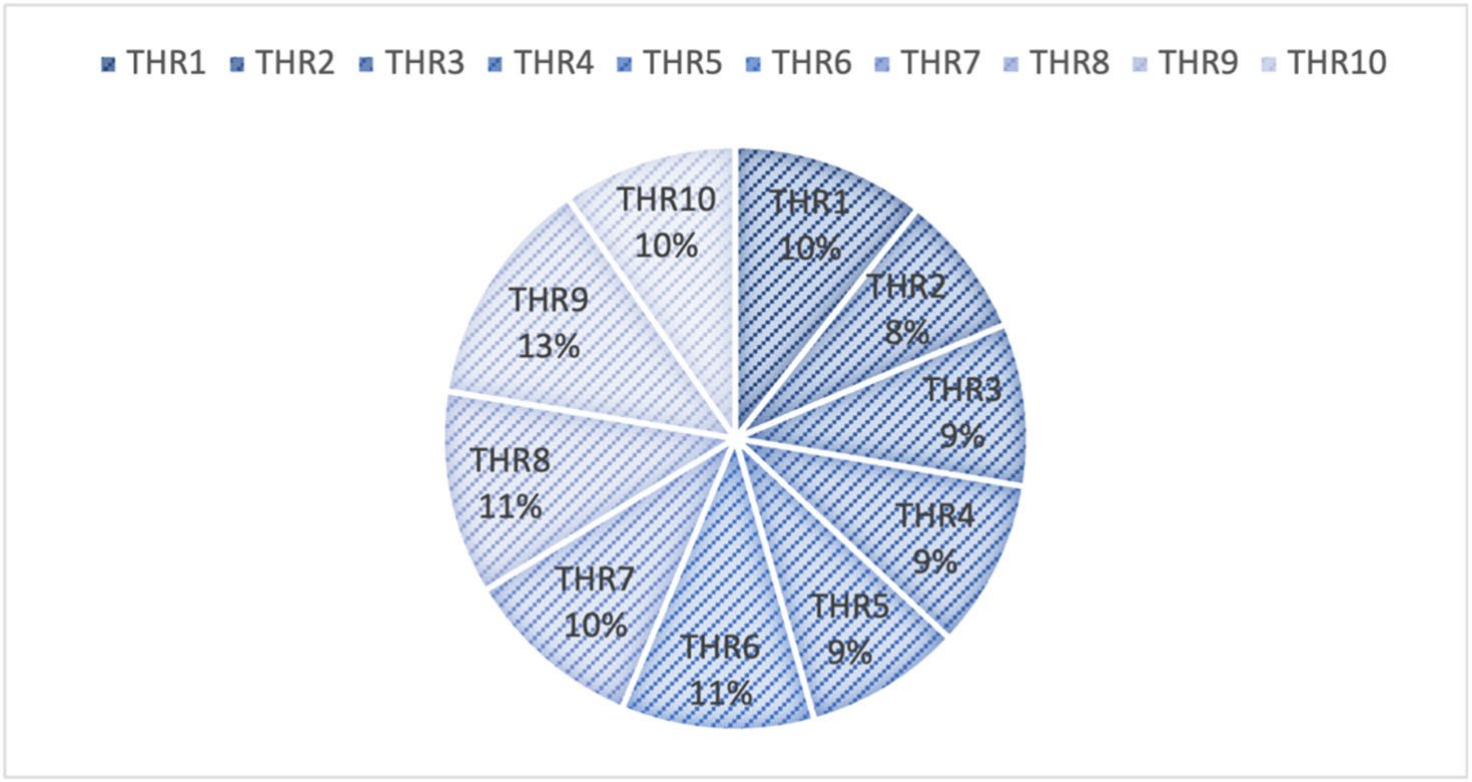
The weights of threats sub-criteria.

**Step 35.** The final weights of the threats sub-criteria are determined using Eq. (19) and visualized in Fig. 5.

This stage finalizes the evaluation of the threats sub-criteria and prepares the groundwork for the subsequent analysis of weaknesses, ensuring that all external and internal risk factors are systematically incorporated into the decision-making process.

#### Results of weakness sub-criteria

The SF-CRITIC method is used to determine the relative importance of the sub-criteria under the *weaknesses* dimension of the SWOT framework. The evaluation is conducted based on expert judgments, which are aggregated, defuzzified, and normalized to compute the weights of the weakness sub-criteria and assess their influence on each alternative.

**Table 13:** The crisp values between threats sub-criteria and alternatives.

	THR_1_	THR _2_	THR _3_	THR _4_	THR _5_	THR _6_	THR _7_	THR _8_	THR _9_	THR _10_
GME_1_	0.475428	0.627801	0.413980	0.569238	0.008933	0.546029	0.588852	0.413980	0.309929	0.363459
GME_2_	0.531787	0.442622	0.572596	0.458225	0.503648	0.556579	0.303387	0.460332	0.005467	0.563475
GME_3_	0.352385	0.356764	0.297313	0.356764	0.380723	0.424002	0.050817	0.337867	0.144421	0.399293
GME_4_	0.200530	0.062174	0.103956	0.193506	0.167842	0.210904	0.201744	0.569238	0.240891	0.512201
GME_5_	0.064399	0.081354	0.226544	0.338413	0.053095	0.087198	0.097985	0.503112	0.474225	0.225204
GME_6_	0.273004	0.317578	0.466206	0.115194	0.060079	0.309733	0.100395	0.077105	0.229045	0.389150
GME_7_	0.144421	0.504811	0.211765	0.055423	0.293224	0.183929	0.363459	0.236369	0.073505	0.609093
GME_8_	0.542820	0.278174	0.437323	0.317429	0.353438	0.158874	0.474608	0.079877	0.157182	0.383093
GME_9_	0.390536	0.185857	0.123705	0.444008	0.280169	0.651421	0.111940	0.140722	0.202346	0.283291
GME_10_	0.538518	0.312421	0.247709	0.036969	0.134693	0.044591	0.156719	0.161025	0.179126	0.573407
GME_11_	0.276757	0.285310	0.171483	0.158426	0.069629	0.389356	0.518280	0.281916	0.398308	0.100663
GME_12_	0.090555	0.221110	0.233362	0.250060	0.332073	0.653371	0.071219	0.153197	0.327367	0.481129
GME_13_	0.040776	0.027381	0.323427	0.003202	0.422171	0.475428	0.382153	0.424153	0.460426	0.202304
GME_14_	0.035229	0.426092	0.188744	0.273641	0.624850	0.296751	0.374916	0.486141	0.421355	0.290510
GME_15_	0.237423	0.327000	0.214808	0.179698	0.066246	0.202377	0.538518	0.211765	0.053682	0.375574
GME_16_	0.429054	0.212709	0.539479	0.563475	0.196752	0.141156	0.356344	0.283633	0.283633	0.379832
GME_17_	0.552364	0.197887	0.408912	0.105448	0.197296	0.503648	0.309602	0.564553	0.460837	0.276533
GME_18_	0.147820	0.287235	0.037570	0.053095	0.132661	0.271511	0.592900	0.341197	0.471861	0.200748
GME_19_	0.207511	0.048660	0.279683	0.393016	0.068695	0.359487	0.062481	0.117191	0.030399	0.231524
GME_20_	0.469659	0.166175	0.416530	0.635599	0.323909	0.019266	0.720000	0.133590	0.250629	0.619024

**Table 14:** The normalized decision matrix between threats sub factors and alternatives by the CRITIC method.

	THR_1_	THR _2_	THR _3_	THR _4_	THR _5_	THR _6_	THR _7_	THR _8_	THR _9_	THR _10_
GME_1_	0.851227	1.000000	0.703536	0.895065	0.000000	0.830718	0.804018	0.684520	0.649507	0.506976
GME_2_	0.960211	0.691585	1.000000	0.719522	0.803216	0.847357	0.377430	0.778705	0.000000	0.892838
GME_3_	0.613295	0.548588	0.485478	0.559082	0.603636	0.638278	0.000000	0.529861	0.296431	0.576105
GME_4_	0.319647	0.057947	0.124080	0.300924	0.258003	0.302218	0.225538	1.000000	0.502228	0.793923
GME_5_	0.056408	0.089891	0.353205	0.530064	0.071701	0.107130	0.070485	0.865634	1.000000	0.240260
GME_6_	0.459793	0.483324	0.801151	0.177091	0.083041	0.458074	0.074086	0.000000	0.476959	0.556536
GME_7_	0.211149	0.795160	0.325581	0.082576	0.461573	0.259677	0.467200	0.323619	0.145146	0.980842
GME_8_	0.981545	0.417697	0.747165	0.496882	0.559337	0.220165	0.633296	0.005634	0.323653	0.544852
GME_9_	0.687069	0.263942	0.160992	0.697040	0.440378	0.996925	0.091339	0.129268	0.420001	0.352318
GME_10_	0.973226	0.474735	0.392765	0.053396	0.204183	0.039938	0.158256	0.170523	0.370466	0.911998
GME_11_	0.467051	0.429581	0.250293	0.245454	0.098546	0.583642	0.698558	0.416169	0.838045	0.000000
GME_12_	0.106986	0.322656	0.365949	0.390353	0.524649	1.000000	0.030488	0.154617	0.686709	0.733979
GME_13_	0.010726	0.000000	0.534286	0.000000	0.670931	0.719379	0.495134	0.705191	0.970563	0.196082
GME_14_	0.000000	0.664055	0.282554	0.427642	1.000000	0.437601	0.484320	0.831148	0.887211	0.366245
GME_15_	0.390990	0.499017	0.331269	0.279090	0.093053	0.288770	0.728800	0.273624	0.102858	0.530346
GME_16_	0.761551	0.308664	0.938102	0.885952	0.304942	0.192223	0.456567	0.419658	0.593410	0.538561
GME_17_	1.000000	0.283977	0.694063	0.161680	0.305825	0.763882	0.386718	0.990480	0.971440	0.339280
GME_18_	0.217722	0.432787	0.000000	0.078895	0.200885	0.397797	0.810066	0.536626	0.994957	0.193079
GME_19_	0.333147	0.035440	0.452525	0.616406	0.097029	0.536537	0.017430	0.081453	0.053187	0.252452
GME_20_	0.840070	0.231162	0.708301	1.000000	0.511393	0.000000	1.000000	0.114776	0.523003	1.000000

**Table 15:** The correlation matrix between threats sub-criteria and weights of criteria.

	THR_1_	THR _2_	THR _3_	THR _4_	THR _5_	THR _6_	THR _7_	THR _8_	THR _9_	THR _10_	$\:ST{D}_{j}$	$\:{W}_{j}$
THR _1_	1.0000	0.2612	0.5890	0.3950	– 0.0714	– 0.0147	0.1789	– 0.1731	– 0.3416	0.3430	0.351586	0.106679
THR _2_	0.2612	1.0000	0.1883	0.0941	0.1069	0.1362	0.3052	– 0.0026	– 0.2474	0.2955	0.261413	0.079610
THR _3_	0.1693	1.0000	0.1045	– 0.1276	0.3440	– 0.0092	0.1739	– 0.1101	– 0.3446	0.3604	0.275255	0.089973
THR _4_	0.3950	0.0941	0.4521	1.0000	0.0848	0.0498	0.1278	– 0.0946	– 0.2354	0.1845	0.303648	0.093406
THR _5_	– 0.0714	0.1069	0.1486	0.0848	1.0000	0.2021	– 0.0019	0.1613	– 0.0408	0.2501	0.274567	0.086782
THR _6_	– 0.0147	0.1362	0.0222	0.0498	0.2021	1.0000	– 0.2572	0.1479	0.0637	– 0.2733	0.308783	0.106723
THR _7_	0.1789	0.3052	0.0830	0.1278	– 0.0019	– 0.2572	1.0000	0.0447	0.1818	– 0.0147	0.308602	0.099836
THR _8_	– 0.1731	– 0.0026	– 0.0811	– 0.0946	0.1613	0.1479	0.0447	1.0000	0.4689	– 0.1972	0.334052	0.112901
THR _9_	– 0.3416	– 0.2474	– 0.2458	– 0.2354	– 0.0408	0.0637	0.1818	0.4689	1.0000	– 0.5627	0.329786	0.127213
THR _10_	0.3430	0.2955	0.2956	0.1845	0.2501	– 0.2733	– 0.0147	– 0.1972	– 0.5627	1.0000	0.288177	0.096877

**Step 36.** The decision matrix between the weakness sub-criteria and the alternatives is constructed using Eq. (11), based on expert evaluations expressed through the linguistic terms defined in Table 1. These terms are converted into corresponding spherical fuzzy numbers (SFNs).

**Step 37.** The individual decision matrices are aggregated into a single matrix using the Weighted Arithmetic Mean (WAM) approach as described in Eq. (6).

**Step 38.** Crisp values for each evaluation are obtained by applying the score function in Eq. (8), and the resulting values are presented in Table 16.

**Step 39.** The normalized decision matrix is calculated using Eq. (13), as shown in Table 17.

**Step 40.** Correlation coefficients between the weakness sub-criteria and alternatives are computed using Eqs. (15) and (16), with the results displayed in Table 18.

**Table 16:** The crisp values between weakness sub-criteria and alternatives.

	WEA_1_	WEA_2_	WEA_3_	WEA_4_	WEA_5_	WEA_6_
GME_1_	0.480198	0.661828	0.399424	0.619403	0.102960	0.549295
GME_2_	0.313090	0.151453	0.608264	0.357695	0.543661	0.290569
GME_3_	0.030002	0.470305	0.387176	0.097702	0.101528	0.234445
GME_4_	0.115540	0.051564	0.164851	0.554357	0.393513	0.068636
GME_5_	0.617428	0.256767	0.272621	0.255586	0.337830	0.164348
GME_6_	0.470945	0.455013	0.385175	0.212256	0.446474	0.611116
GME_7_	0.481404	0.340310	0.149693	0.106417	0.353969	0.088158
GME_8_	0.437705	0.065686	0.223928	0.223928	0.001966	0.219017
GME_9_	0.042776	0.086519	0.281698	0.463022	0.572042	0.442944
GME_10_	0.285300	0.608540	0.282546	0.215210	0.215685	0.061027
GME_11_	0.373352	0.230885	0.288025	0.241014	0.334152	0.327313
GME_12_	0.372227	0.311890	0.300183	0.217784	0.460397	0.388639
GME_13_	0.475978	0.411897	0.400910	0.576690	0.459245	0.542570
GME_14_	0.071950	0.379058	0.230075	0.003125	0.379370	0.340576
GME_15_	0.390251	0.160877	0.244697	0.012002	0.109338	0.558328
GME_16_	0.211519	0.231767	0.297793	0.387000	0.372227	0.148499
GME_17_	0.464206	0.445634	0.076376	0.446943	0.521675	0.400486
GME_18_	0.111429	0.241844	0.072288	0.235326	0.330503	0.397254
GME_19_	0.390714	0.210382	0.285445	0.417579	0.608059	0.257653
GME_20_	0.455013	0.324827	0.468617	0.606143	0.296037	0.202320

**Table 17:** The normalized decision matrix between weakness sub-criteria and alternatives by the CRITIC method.

	WEA_1_	WEA_2_	WEA_3_	WEA_4_	WEA_5_	WEA_6_
GME_1_	0.766386	1.000000	0.610356	1.000000	0.166631	0.887617
GME_2_	0.481912	0.163681	1.000000	0.575340	0.893749	0.417282
GME_3_	0.000000	0.686163	0.587503	0.153465	0.164268	0.315254
GME_4_	0.145614	0.000000	0.172700	0.894453	0.646018	0.013832
GME_5_	1.000000	0.336252	0.373773	0.409655	0.554146	0.187827
GME_6_	0.750636	0.661105	0.583770	0.339345	0.733399	1.000000
GME_7_	0.768440	0.473150	0.144419	0.167607	0.580774	0.049322
GME_8_	0.694049	0.023139	0.282923	0.358285	0.000000	0.287208
GME_9_	0.021746	0.057278	0.390707	0.746250	0.940575	0.694282
GME_10_	0.434604	0.912680	0.392290	0.344139	0.352617	0.000000
GME_11_	0.584498	0.293842	0.402512	0.386010	0.548077	0.484078
GME_12_	0.582583	0.426579	0.425195	0.348315	0.756371	0.595562
GME_13_	0.759204	0.590454	0.613128	0.930692	0.754470	0.875390
GME_14_	0.071409	0.536643	0.294391	0.000000	0.622683	0.508190
GME_15_	0.613266	0.179123	0.321673	0.014404	0.177154	0.904038
GME_16_	0.309004	0.295287	0.420737	0.622892	0.610898	0.159014
GME_17_	0.739163	0.645737	0.007628	0.720158	0.857474	0.617099
GME_18_	0.138616	0.311800	0.000000	0.376779	0.542057	0.611223
GME_19_	0.614055	0.260244	0.397698	0.672512	1.000000	0.357444
GME_20_	0.723513	0.447779	0.739453	0.978483	0.485192	0.256855

**Table 18:** The correlation matrix between weakness sub-criteria and weights of criteria.

	WEA_1_	WEA_2_	WEA_3_	WEA_4_	WEA_5_	WEA_6_	$\:ST{D}_{j}$	$\:{W}_{j}$
WEA_1_	1.0000	0.2139	0.1430	0.1745	– 0.0507	0.1451	0.296057	0.166642
WEA_2_	0.2139	1.0000	0.1560	0.0202	– 0.2056	0.2024	0.278130	0.165099
WEA_3_	0.1328	1.0000	0.0679	– 0.2076	– 0.0457	0.0507	0.240218	0.154612
WEA_4_	0.1745	0.0202	0.2649	1.0000	0.3009	0.0510	0.309967	0.167062
WEA_5_	– 0.0507	– 0.2056	0.0373	0.3009	1.0000	0.0874	0.278907	0.173372
WEA_6_	0.1451	0.2024	0.1604	0.0510	0.0874	1.0000	0.309182	0.173212

**Step 41.** The standard deviation for each weakness sub-criterion is determined using Eq. (17), as included in Table 18.

**Step 42.** The amount of information provided by each sub-criterion is calculated using Eq. (18), also shown in Table 18.

**Fig. 6:**
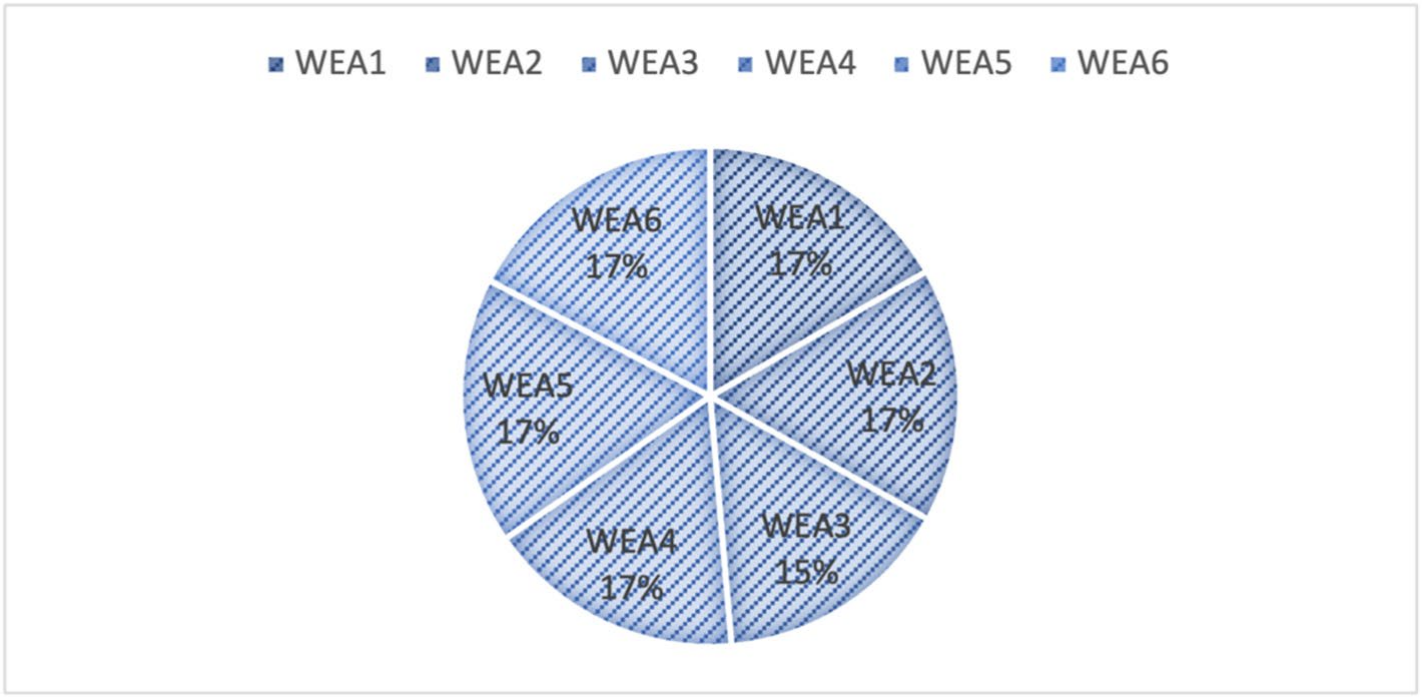
The weights of weakness sub-criteria.

**Step 43.** The final weights of the weakness sub-criteria are computed using Eq. (19), and the results are visualized in Fig. 6.

These findings highlight the impact of internal limitations on the decision-making process and contribute to a comprehensive prioritization of factors affecting the green conversion potential of gold mining operations.

#### Results of SWOT global criteria

**Fig. 7:**
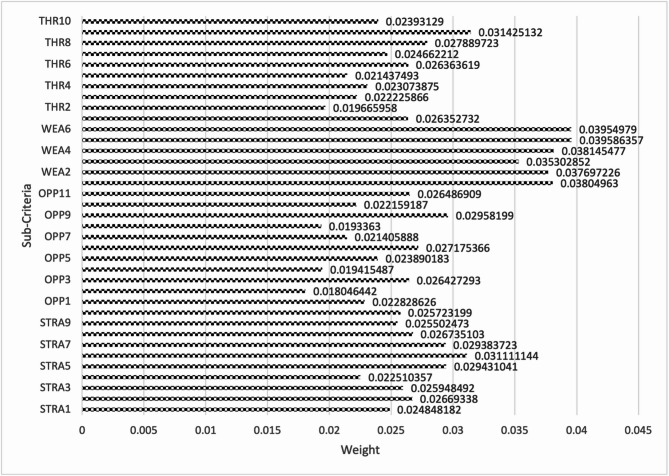
The weights of global criteria.

To assess the overall importance of each sub-criterion across the SWOT dimensions, the global weights are calculated by multiplying the local weights of sub-criteria by the corresponding weights of their main criteria. This approach ensures that both the internal priority of a sub-factor and the significance of its parent category are taken into consideration in the final evaluation. The results of the global weights computation are illustrated in Fig. 7. Among all sub-criteria, WEA5 (Weakness 5) attains the highest global weight, indicating its critical influence in the assessment of green mine conversion. Conversely, OPP2 (Opportunity 2) records the lowest weight, suggesting a relatively minimal impact compared to other factors. These global weights provide a comprehensive basis for prioritizing the evaluation criteria, enabling more informed and balanced decision-making in the context of sustainable mining transformation.

### Results of the SF-GRA method

Following the determination of global criteria weights, the Spherical Fuzzy Grey Relational Analysis (SF-GRA) method is applied to evaluate and rank the gold mine alternatives. This method assesses the proximity of each alternative to an ideal reference by incorporating normalized performance values and relational coefficients, thereby enabling robust decision-making under uncertainty.

**Step 44.** The decision matrix between the global evaluation criteria and the alternatives is constructed using linguistic terms provided by five experts, as defined in Table 1 and detailed across Tables A1 to A5. These linguistic terms are subsequently converted into their corresponding spherical fuzzy numbers (SFNs) based on Eq. (11).

**Step 45.** The individual expert matrices are aggregated into a single collective decision matrix using the Weighted Averaging Method (WAM), as outlined in Eq. (6).

**Step 46.** Crisp values for each criterion-alternative pair are computed by applying the score function in Eq. (8). The resulting matrix is presented in Table 19.

**Step 47.** The decision matrix is then normalized using the GRA normalization approach, as expressed in Eq. (20), and the normalized values are shown in Table 20.

**Step 48.** The reference series, representing the ideal performance across criteria, is determined using Eqs. (22) and (23).

**Step 49.** The distance matrix between the alternatives and the reference series is calculated using Eqs. (24) and (25), capturing deviation from the ideal.

**Step 50.** Grey relational coefficients are computed for each alternative with respect to the reference using Eq. (26), with the distinguishing coefficient η set to 0.5.

**Fig. 8:**
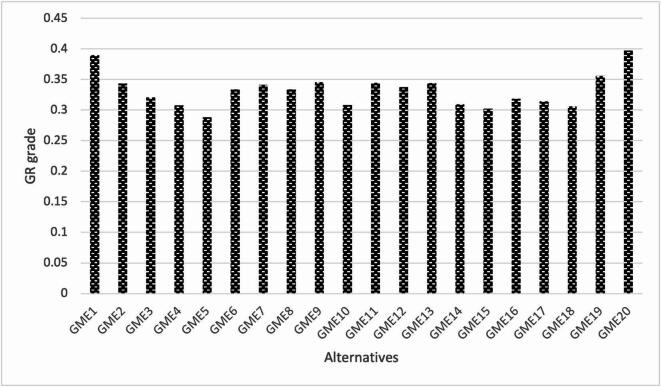
The grey relational grade.

**Step 51.** The grey relational grade for each alternative is calculated using Eq. (27), which aggregates the relational coefficients into a single performance index. The results are visualized in Fig. 8.

**Step 52.** Based on the computed grey relational grades, the alternatives are ranked. As shown in Fig. 8, GME20 achieves the highest score and is identified as the most suitable candidate for green conversion, whereas GME5 records the lowest grade, indicating the least suitability.

The highest-ranked mine, GME20, demonstrated superior performance across multiple sustainability indicators. Its high scores in selected strengths and opportunities criteria, combined with relatively low weaknesses and threats, reflect a balanced and proactive operational profile. These factors align with key national sustainability goals and international environmental standards. The proposed framework supports such evaluations by offering decision-makers a structured tool to prioritize green investment and develop targeted interventions for lower-performing mines.

**Table 19:** The crisp values of the decision matrix between overall criteria and alternatives.

	STRA1	STRA2	STRA3	STRA4	STRA5	STRA6	STRA7	STRA8	STRA9	STRA10
GME1	0.232	0.601	0.452	0.568	0.343	0.390	0.203	0.356	0.169	0.477
GME2	0.465	0.565	0.461	0.367	0.331	0.399	0.390	0.267	0.086	0.550
GME3	0.409	0.009	0.027	0.040	0.253	0.209	0.063	0.294	0.128	0.486
GME4	0.185	0.219	0.167	0.004	0.214	0.165	0.146	0.206	0.356	0.206
GME5	0.415	0.475	0.106	0.150	0.072	0.034	0.012	0.036	0.407	0.138
GME6	0.275	0.039	0.003	0.527	0.136	0.412	0.351	0.413	0.362	0.438
GME7	0.280	0.244	0.457	0.609	0.596	0.516	0.292	0.126	0.048	0.434
GME8	0.399	0.102	0.310	0.068	0.330	0.280	0.340	0.193	0.289	0.482
GME9	0.381	0.153	0.152	0.010	0.429	0.008	0.296	0.099	0.515	0.505
GME10	0.390	0.138	0.088	0.071	0.390	0.044	0.193	0.122	0.040	0.338
GME11	0.555	0.551	0.057	0.000	0.198	0.148	0.466	0.332	0.424	0.382
GME12	0.253	0.377	0.325	0.345	0.529	0.478	0.120	0.446	0.241	0.236
GME13	0.346	0.258	0.246	0.314	0.205	0.414	0.565	0.183	0.129	0.194
GME14	0.094	0.124	0.155	0.280	0.310	0.199	0.506	0.120	0.360	0.327
GME15	0.382	0.311	0.293	0.080	0.199	0.348	0.341	0.248	0.310	0.357
GME16	0.204	0.160	0.547	0.360	0.127	0.289	0.209	0.212	0.004	0.405
GME17	0.088	0.063	0.106	0.026	0.223	0.131	0.083	0.258	0.209	0.105
GME18	0.044	0.013	0.253	0.176	0.043	0.118	0.446	0.328	0.225	0.044
GME19	0.527	0.423	0.250	0.246	0.565	0.468	0.231	0.058	0.382	0.183
GME20	0.471	0.256	0.515	0.558	0.399	0.415	0.218	0.028	0.720	0.371

	OPP1	OPP2	OPP3	OPP4	OPP5	OPP6	OPP7	OPP8	OPP9	OPP10	OPP11
GME1	0.480	0.477	0.226	0.720	0.292	0.421	0.381	0.270	0.440	0.480	0.075
GME2	0.636	0.546	0.607	0.431	0.512	0.007	0.036	0.240	0.082	0.120	0.237
GME3	0.086	0.119	0.273	0.495	0.037	0.074	0.248	0.077	0.026	0.097	0.299
GME4	0.125	0.004	0.190	0.249	0.397	0.252	0.009	0.304	0.274	0.240	0.289
GME5	0.166	0.105	0.336	0.166	0.161	0.064	0.342	0.186	0.167	0.061	0.110
GME6	0.207	0.438	0.506	0.086	0.191	0.308	0.048	0.660	0.198	0.443	0.178
GME7	0.404	0.539	0.016	0.327	0.167	0.184	0.066	0.343	0.398	0.145	0.041
GME8	0.008	0.207	0.219	0.305	0.361	0.378	0.357	0.122	0.294	0.274	0.008
GME9	0.028	0.267	0.425	0.349	0.452	0.140	0.102	0.281	0.162	0.404	0.266
GME10	0.392	0.062	0.128	0.039	0.222	0.231	0.075	0.071	0.242	0.349	0.558
GME11	0.080	0.117	0.169	0.193	0.223	0.345	0.480	0.291	0.064	0.179	0.680
GME12	0.438	0.429	0.480	0.091	0.034	0.142	0.383	0.461	0.265	0.119	0.120
GME13	0.539	0.437	0.209	0.436	0.229	0.075	0.338	0.170	0.520	0.189	0.018
GME14	0.107	0.134	0.089	0.625	0.359	0.310	0.262	0.152	0.105	0.218	0.397
GME15	0.187	0.093	0.247	0.169	0.256	0.077	0.169	0.286	0.568	0.079	0.365
GME16	0.142	0.445	0.591	0.039	0.191	0.249	0.135	0.144	0.111	0.270	0.510
GME17	0.280	0.191	0.207	0.071	0.117	0.363	0.377	0.269	0.272	0.094	0.167
GME18	0.265	0.079	0.381	0.241	0.459	0.187	0.214	0.079	0.154	0.452	0.277
GME19	0.183	0.209	0.394	0.031	0.142	0.433	0.385	0.364	0.342	0.558	0.550
GME20	0.371	0.311	0.460	0.493	0.480	0.328	0.399	0.224	0.226	0.120	0.720

	WEA1	WEA2	WEA3	WEA4	WEA5	WEA6	THR1	THR2	THR3	THR4	THR5	THR6	THR7	THR8	THR9	THR10
GME1	0.120	0.292	0.480	0.720	0.391	0.390	0.547	0.720	0.720	0.480	0.431	0.258	0.625	0.120	0.625	0.545
GME2	0.365	0.328	0.181	0.480	0.286	0.154	0.211	0.134	0.452	0.075	0.079	0.009	0.296	0.374	0.328	0.172
GME3	0.079	0.555	0.276	0.625	0.442	0.195	0.385	0.344	0.267	0.452	0.365	0.120	0.079	0.485	0.522	0.390
GME4	0.345	0.182	0.188	0.004	0.072	0.411	0.482	0.436	0.383	0.165	0.596	0.002	0.129	0.197	0.086	0.502
GME5	0.142	0.089	0.194	0.394	0.119	0.167	0.071	0.086	0.142	0.289	0.027	0.333	0.104	0.087	0.125	0.024
GME6	0.155	0.201	0.028	0.380	0.500	0.401	0.266	0.188	0.358	0.151	0.383	0.346	0.365	0.411	0.184	0.117
GME7	0.071	0.558	0.507	0.139	0.479	0.303	0.064	0.010	0.237	0.209	0.010	0.537	0.541	0.313	0.407	0.233
GME8	0.139	0.647	0.363	0.219	0.574	0.280	0.219	0.352	0.196	0.305	0.047	0.070	0.533	0.507	0.429	0.129
GME9	0.480	0.720	0.575	0.647	0.120	0.232	0.549	0.399	0.652	0.527	0.201	0.416	0.074	0.435	0.127	0.064
GME10	0.436	0.360	0.195	0.168	0.280	0.209	0.117	0.280	0.039	0.292	0.202	0.144	0.235	0.455	0.388	0.416
GME11	0.005	0.001	0.038	0.330	0.720	0.555	0.346	0.625	0.134	0.498	0.222	0.571	0.168	0.401	0.179	0.144
GME12	0.476	0.387	0.272	0.296	0.298	0.131	0.533	0.099	0.498	0.207	0.431	0.034	0.213	0.333	0.234	0.272
GME13	0.136	0.648	0.148	0.457	0.026	0.191	0.431	0.323	0.394	0.498	0.153	0.565	0.577	0.252	0.438	0.546
GME14	0.106	0.382	0.188	0.232	0.363	0.107	0.070	0.290	0.226	0.394	0.201	0.415	0.280	0.175	0.021	0.061
GME15	0.008	0.374	0.249	0.032	0.010	0.042	0.229	0.021	0.039	0.182	0.121	0.155	0.126	0.188	0.125	0.125
GME16	0.064	0.546	0.477	0.064	0.045	0.127	0.431	0.266	0.084	0.128	0.047	0.280	0.555	0.128	0.080	0.176
GME17	0.173	0.129	0.720	0.009	0.105	0.105	0.077	0.720	0.516	0.625	0.085	0.652	0.182	0.625	0.023	0.138
GME18	0.537	0.039	0.039	0.382	0.103	0.248	0.021	0.039	0.059	0.382	0.512	0.120	0.128	0.382	0.198	0.039
GME19	0.299	0.280	0.280	0.232	0.219	0.545	0.313	0.280	0.720	0.280	0.680	0.280	0.183	0.280	0.280	0.480
GME20	0.720	0.720	0.480	0.512	0.601	0.382	0.537	0.120	0.039	0.480	0.120	0.720	0.371	0.720	0.480	0.720

**Table 20:** The normalization decision matrix between overall criteria and alternatives by the GRA method.

	STRA1	STRA2	STRA3	STRA4	STRA5	STRA6	STRA7	STRA8	STRA9	STRA10
GME1	0.42	1.00	0.83	0.93	0.58	0.76	0.36	0.80	0.24	0.87
GME2	0.84	0.94	0.84	0.60	0.56	0.77	0.69	0.60	0.12	1.00
GME3	0.74	0.01	0.05	0.07	0.42	0.40	0.11	0.66	0.18	0.88
GME4	0.33	0.36	0.31	0.01	0.36	0.32	0.26	0.46	0.49	0.37
GME5	0.75	0.79	0.19	0.25	0.12	0.07	0.02	0.08	0.56	0.25
GME6	0.50	0.06	0.01	0.87	0.23	0.80	0.62	0.93	0.50	0.80
GME7	0.50	0.41	0.84	1.00	1.00	1.00	0.52	0.28	0.07	0.79
GME8	0.72	0.17	0.57	0.11	0.55	0.54	0.60	0.43	0.40	0.88
GME9	0.69	0.25	0.28	0.02	0.72	0.02	0.53	0.22	0.71	0.92
GME10	0.70	0.23	0.16	0.12	0.65	0.08	0.34	0.27	0.06	0.61
GME11	1.00	0.92	0.10	0.00	0.33	0.29	0.83	0.74	0.59	0.69
GME12	0.46	0.63	0.59	0.57	0.89	0.93	0.21	1.00	0.33	0.43
GME13	0.62	0.43	0.45	0.52	0.34	0.80	1.00	0.41	0.18	0.35
GME14	0.17	0.21	0.28	0.46	0.52	0.39	0.90	0.27	0.50	0.59
GME15	0.69	0.52	0.54	0.13	0.33	0.67	0.60	0.56	0.43	0.65
GME16	0.37	0.27	1.00	0.59	0.21	0.56	0.37	0.48	0.00	0.74
GME17	0.16	0.11	0.19	0.04	0.37	0.25	0.15	0.58	0.29	0.19
GME18	0.08	0.02	0.46	0.29	0.07	0.23	0.79	0.73	0.31	0.08
GME19	0.95	0.70	0.46	0.40	0.95	0.91	0.41	0.13	0.53	0.33
GME20	0.85	0.43	0.94	0.92	0.67	0.80	0.39	0.06	1.00	0.67

	OPP1	OPP2	OPP3	OPP4	OPP5	OPP6	OPP7	OPP8	OPP9	OPP10	OPP11
GME1	0.76	0.87	0.37	1.00	0.57	0.97	0.79	0.41	0.77	0.86	0.10
GME2	1.00	1.00	1.00	0.60	1.00	0.02	0.07	0.36	0.14	0.21	0.33
GME3	0.14	0.22	0.45	0.69	0.07	0.17	0.52	0.12	0.05	0.17	0.42
GME4	0.20	0.01	0.31	0.35	0.78	0.58	0.02	0.46	0.48	0.43	0.40
GME5	0.26	0.19	0.55	0.23	0.31	0.15	0.71	0.28	0.29	0.11	0.15
GME6	0.33	0.80	0.83	0.12	0.37	0.71	0.10	1.00	0.35	0.79	0.25
GME7	0.64	0.99	0.03	0.45	0.33	0.42	0.14	0.52	0.70	0.26	0.06
GME8	0.01	0.38	0.36	0.42	0.71	0.87	0.74	0.19	0.52	0.49	0.01
GME9	0.04	0.49	0.70	0.49	0.88	0.32	0.21	0.43	0.29	0.72	0.37
GME10	0.62	0.11	0.21	0.05	0.43	0.53	0.16	0.11	0.43	0.63	0.78
GME11	0.13	0.21	0.28	0.27	0.43	0.80	1.00	0.44	0.11	0.32	0.94
GME12	0.69	0.79	0.79	0.13	0.07	0.33	0.80	0.70	0.47	0.21	0.17
GME13	0.85	0.80	0.34	0.61	0.45	0.17	0.70	0.26	0.92	0.34	0.02
GME14	0.17	0.25	0.15	0.87	0.70	0.71	0.55	0.23	0.18	0.39	0.55
GME15	0.29	0.17	0.41	0.24	0.50	0.18	0.35	0.43	1.00	0.14	0.51
GME16	0.22	0.82	0.97	0.05	0.37	0.57	0.28	0.22	0.20	0.48	0.71
GME17	0.44	0.35	0.34	0.10	0.23	0.84	0.78	0.41	0.48	0.17	0.23
GME18	0.42	0.14	0.63	0.33	0.90	0.43	0.44	0.12	0.27	0.81	0.38
GME19	0.29	0.38	0.65	0.04	0.28	1.00	0.80	0.55	0.60	1.00	0.76
GME20	0.58	0.57	0.76	0.69	0.94	0.76	0.83	0.34	0.40	0.21	1.00

	WEA1	WEA2	WEA3	WEA4	WEA5	WEA6	THR1	THR2	THR3	THR4	THR5	THR6	THR7	THR8	THR9	THR10
GME1	0.17	0.41	0.67	1.00	0.54	0.70	1.00	1.00	1.00	0.77	0.63	0.36	1.00	0.17	1.00	0.76
GME2	0.51	0.46	0.25	0.67	0.40	0.28	0.38	0.19	0.63	0.12	0.12	0.01	0.47	0.52	0.52	0.24
GME3	0.11	0.77	0.38	0.87	0.61	0.35	0.70	0.48	0.37	0.72	0.54	0.17	0.13	0.67	0.83	0.54
GME4	0.48	0.25	0.26	0.01	0.10	0.74	0.88	0.61	0.53	0.26	0.88	0.00	0.21	0.27	0.14	0.70
GME5	0.20	0.12	0.27	0.55	0.16	0.30	0.13	0.12	0.20	0.46	0.04	0.46	0.17	0.12	0.20	0.03
GME6	0.22	0.28	0.04	0.53	0.69	0.72	0.48	0.26	0.50	0.24	0.56	0.48	0.58	0.57	0.29	0.16
GME7	0.10	0.78	0.70	0.19	0.66	0.55	0.12	0.01	0.33	0.33	0.01	0.75	0.87	0.43	0.65	0.32
GME8	0.19	0.90	0.50	0.30	0.80	0.50	0.40	0.49	0.27	0.49	0.07	0.10	0.85	0.70	0.69	0.18
GME9	0.67	1.00	0.80	0.90	0.17	0.42	1.00	0.55	0.90	0.84	0.30	0.58	0.12	0.60	0.20	0.09
GME10	0.61	0.50	0.27	0.23	0.39	0.38	0.21	0.39	0.05	0.47	0.30	0.20	0.38	0.63	0.62	0.58
GME11	0.01	0.00	0.05	0.46	1.00	1.00	0.63	0.87	0.19	0.80	0.33	0.79	0.27	0.56	0.29	0.20
GME12	0.66	0.54	0.38	0.41	0.41	0.24	0.97	0.14	0.69	0.33	0.63	0.05	0.34	0.46	0.37	0.38
GME13	0.19	0.90	0.21	0.64	0.04	0.34	0.78	0.45	0.55	0.80	0.23	0.79	0.92	0.35	0.70	0.76
GME14	0.15	0.53	0.26	0.32	0.50	0.19	0.13	0.40	0.31	0.63	0.30	0.58	0.45	0.24	0.03	0.08
GME15	0.01	0.52	0.35	0.04	0.01	0.08	0.42	0.03	0.05	0.29	0.18	0.21	0.20	0.26	0.20	0.17
GME16	0.09	0.76	0.66	0.09	0.06	0.23	0.78	0.37	0.12	0.21	0.07	0.39	0.89	0.18	0.13	0.24
GME17	0.24	0.18	1.00	0.01	0.15	0.19	0.14	1.00	0.72	1.00	0.13	0.90	0.29	0.87	0.04	0.19
GME18	0.75	0.05	0.05	0.53	0.14	0.45	0.04	0.05	0.08	0.61	0.75	0.17	0.21	0.53	0.32	0.05
GME19	0.41	0.39	0.39	0.32	0.30	0.98	0.57	0.39	1.00	0.45	1.00	0.39	0.29	0.39	0.45	0.67
GME20	1.00	1.00	0.67	0.71	0.83	0.69	0.98	0.17	0.05	0.77	0.18	1.00	0.59	1.00	0.77	1.00

## Sensitivity analysis

To validate the robustness of the proposed methodology, a comprehensive sensitivity analysis was conducted. This analysis includes two scenarios: the first examines the effect of varying the grey relational coefficient ($\:\eta\:$) used in the SF-GRA method, and the second investigates the impact of changing the weights of the criteria to simulate different decision-making preferences.

### Variation of the grey relational coefficient

The Grey Relational coefficient ($\:\eta\:)$ in GRA determines the trade-off between the maximum and minimum deviation when calculating the Grey Relational Coefficient (GRC). The default value ( $\:\eta\:$ = 0.5) is typically used to reflect a balanced perspective. However, to test the sensitivity of the model to this parameter, ( $\:\eta\:$) is systematically varied from 0.1 to 1.0 in increments of 0.1.

**Fig. 9:**
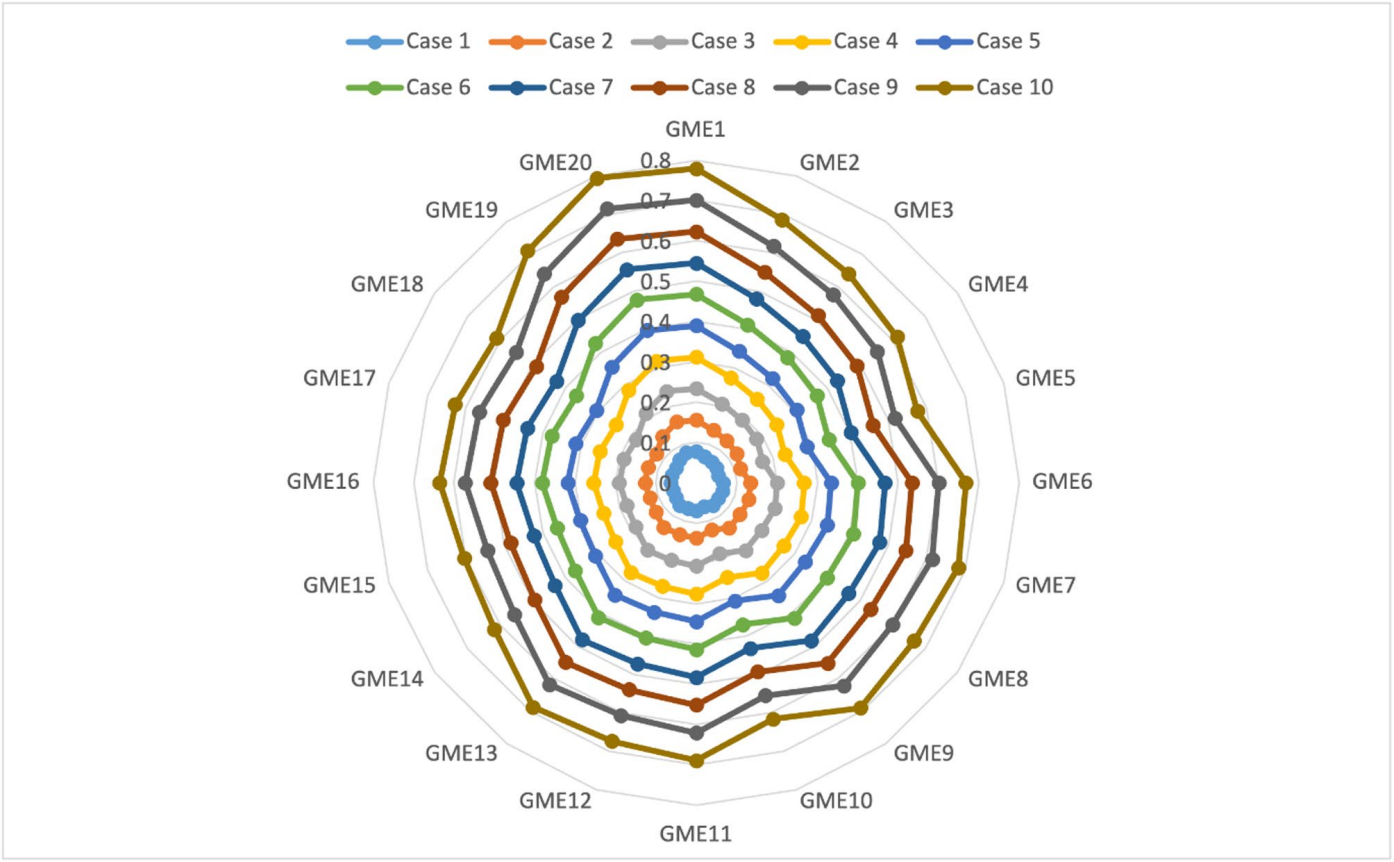
The rank of alternatives under different values of η.

For each value of ($\:\eta\:$), the SF-GRA method is executed completely, and the resulting rankings of the 20 gold mines are recorded. The outcomes are plotted in Fig. 9. Across all values, GME20 consistently ranks highest, while GME5 remains the lowest-ranked alternative. This consistency confirms that the proposed framework is stable and robust to variations in the ($\:\eta\:$) parameter, ensuring that the conclusions are not overly sensitive to the choice of this coefficient.

### Variation in criteria weights

In this scenario, the influence of changing the weights of evaluation criteria is investigated. A total of 37 cases are constructed, each representing a different weighting scenario where one criterion is emphasized while the others are proportionally reduced.

#### Step 1

Initialization.

The original weights of the 37 criteria are denoted by $\:\{{w}_{1},\:{w}_{2},\:.\:.\:.\:.\:.\:,\:{w}_{37}\}$, such that:

28 $$\:\sum\:_{i=1}^{37}{w}_{i}=1$$

#### Step 2

Increase one criterion by 20%.

In each case, the weight of one criterion $\:{w}_{i}$ is increased by 20% to obtain a new weight:

29 $$\:{w}_{i}^{{\prime\:}}=1.2\:\times\:\:{w}_{i}$$

#### Step 3

Adjust other weights to maintain normalization.

To ensure that the sum of the weights remains equal to one, the remaining weights are scaled proportionally using the formula:

30 $$\:{w}_{j}^{{\prime\:}}={w}_{i}\times\:\frac{1-{w}_{i}^{{\prime\:}}}{1-{w}_{i}},\:\:\:\:\:\text{f}\text{o}\text{r}\:\text{a}\text{l}\text{l}\:j\ne\:i$$

This transformation maintains the constraint:

31 $$\:\sum\:_{j=1,\:j\ne\:i}^{37}{w}_{j}^{{\prime\:}}+{w}_{i}^{{\prime\:}}=1$$

This approach follows the weight perturbation methodology commonly used in sensitivity analysis, where one criterion’s weight is increased while the remaining weights are proportionally adjusted to maintain normalization.

#### Step 4

Repeat for each criterion.

**Fig. 10:**
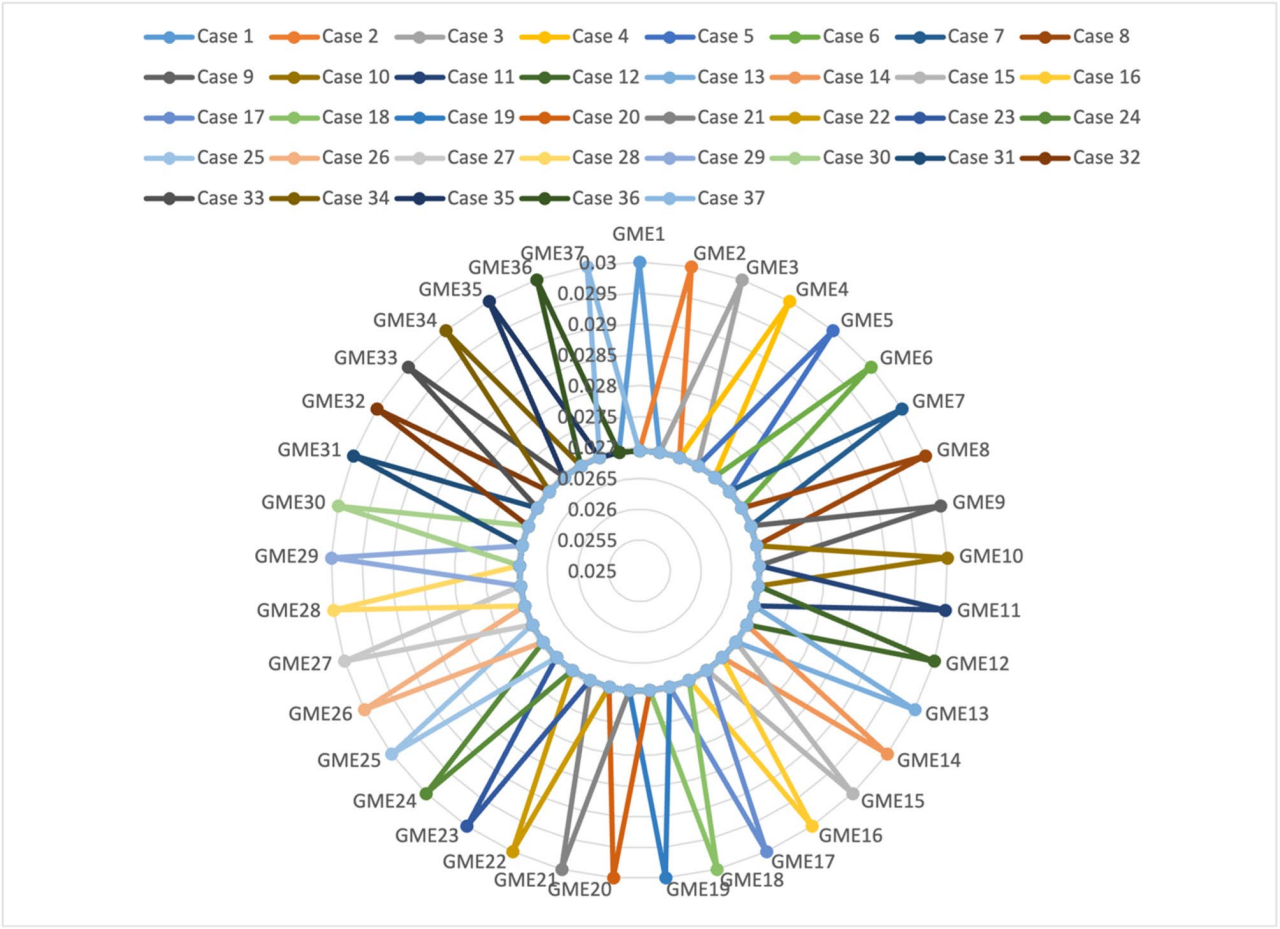
The 37 cases in changing the weights of criteria.

This procedure is repeated 37 times, once for each criterion, resulting in 37 unique weight distributions. An example of the adjusted weights for Case 1 is shown in Fig. 10.

#### Step 5

Recalculate mine rankings.

For each of the 37 weight scenarios, the decision-making process is re-executed using the updated weights, and the final rankings of the 20 gold mines are recorded.

**Fig. 11:**
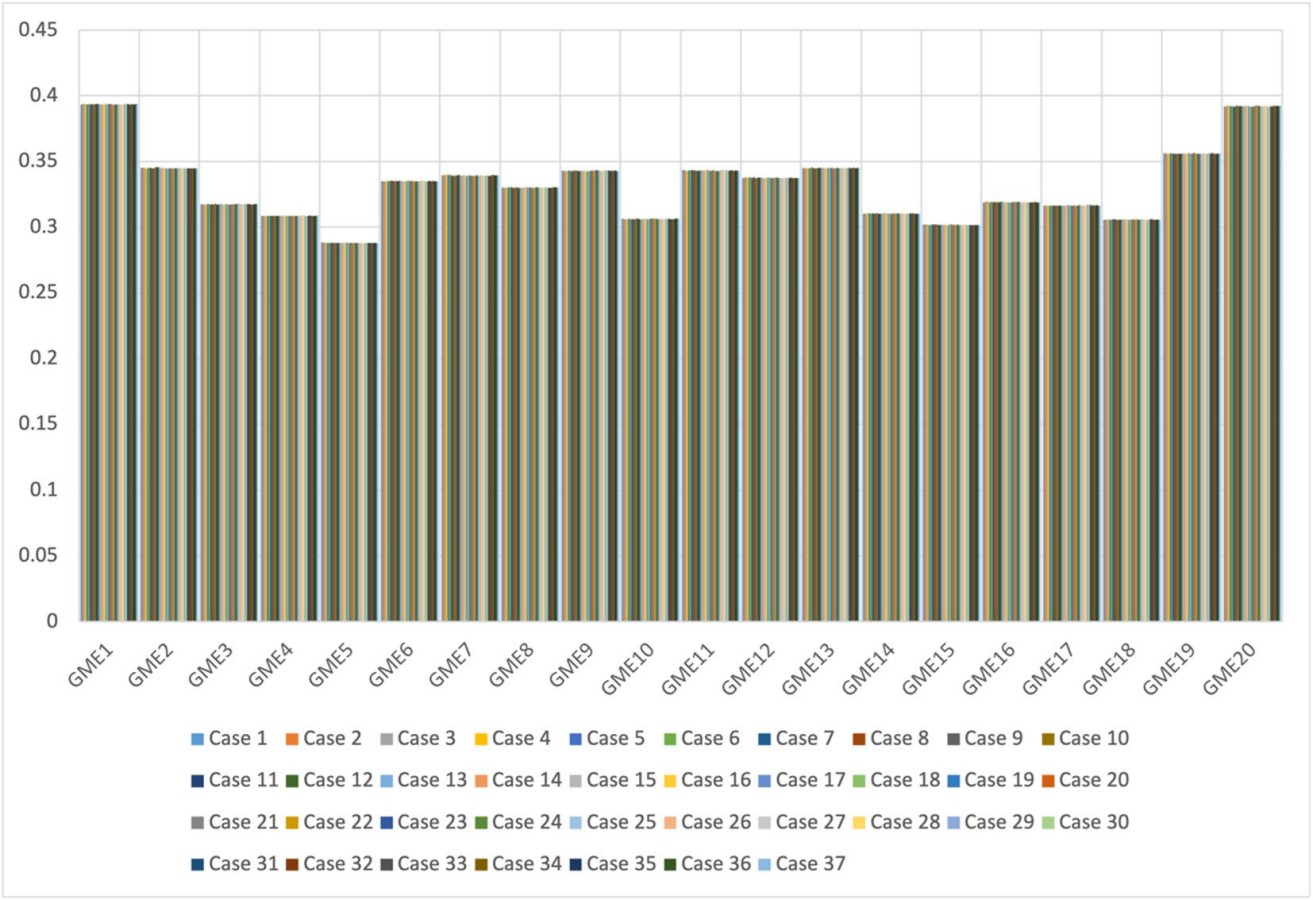
The rank of alternatives under different 37 cases.

The results across all scenarios are visualized in Fig. 11. The findings show that GME1 consistently ranks as the top-performing alternative, while GME5 remains at the bottom across all cases. This indicates a strong level of resilience in the proposed framework, as changes in decision-maker preferences do not significantly alter the conclusions of the evaluation.

The sensitivity analysis confirms that the proposed SF-SWOT-CRITIC-GRA model produces stable and reliable outcomes. Neither variation in the Grey relational coefficient ($\:\eta\:$) nor alterations in criteria weights lead to rank reversals or significant instability in the evaluation results. This robustness enhances the credibility of the model for real-world decision-making applications in sustainable mining.

Although the present sensitivity analysis focuses on variations in the Grey relational coefficient and the weights of evaluation criteria, the proposed framework is also theoretically robust to changes in the number of experts and alternatives. The use of spherical fuzzy weighted aggregation allows seamless integration of additional expert inputs without distorting the underlying evaluations. Similarly, the SF-GRA method accommodates varying numbers of alternatives without requiring model reconfiguration. While a formal simulation of expert count variation or alternative set expansion is beyond the scope of this paper, the model’s modular design and non-compensatory ranking logic mitigate the risk of rank reversal and support its extensibility in future applications.

## Comparative analysis

This section presents a comparative analysis of the proposed methodology against ten established MCDM methods. The selected methods include TOPSIS^41^, Multi-Attributive Border Approximation Area Comparison (MABAC)^42^, VIekriterijumsko KOmpromisno Rangiranje (VIKOR)^43^, Additive Ratio Assessment (ARAS)^44^, Evaluation Based on Distance from Average Solution (EDAS)^45^, Weighted Aggregated Sum Product Assessment (WASPAS)^46^, Multi-Objective Optimization based on Ratio Analysis plus full multiplicative form (MULTIMOOEA)^47^, Complex Proportional Assessment (COPRAS)^48^, Combinative Distance-based Assessment (CODAS)^49^, and Combined Compromise Solution (COCOSO)^50^. These methods were selected based on their suitability for different types of decision-making problems.

**Fig. 12:**
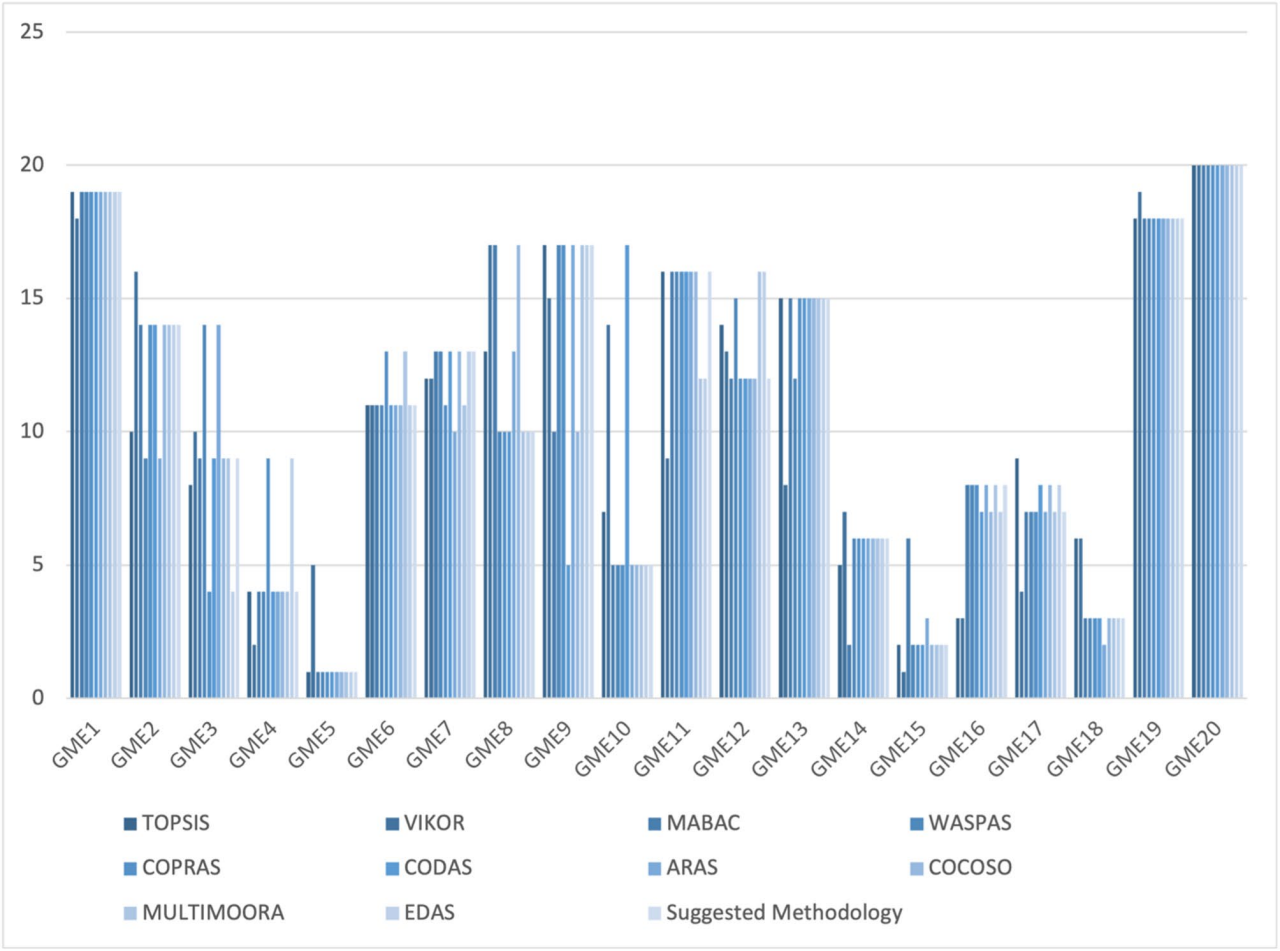
The rank of alternatives under comparative analysis.

To evaluate the effectiveness and reliability of the proposed methodology, its performance was compared with the ten selected MCDM methods. The global weights derived using the SF-CRITIC method were uniformly applied as input weights across all methods. Each method was subsequently used to rank the set of alternatives. The comparative results, presented in Fig. 12, show that all methods, except for VIKOR, identified alternative 5 (GME5) as the lowest-ranked option. VIKOR ranked alternative 15 (GME15) as the least preferred. All methods consistently identified alternative 20 (GME20) as the best-performing option. While most methods consistently ranked GME20 as the top-performing alternative and GME5 as the lowest, VIKOR diverged by ranking GME15 as the least preferred option. This discrepancy arises from VIKOR’s emphasis on achieving a compromise solution that minimizes individual regret. Unlike other methods that balance criteria performance more holistically, VIKOR assigns additional weight to the worst-performing criterion for each alternative. GME15 displayed a high disparity between strong and weak criteria scores, which triggered a stronger penalization under VIKOR’s logic. This highlights how different aggregation mechanisms can lead to variation in alternative rankings and underscores the need to align method selection with stakeholder objectives.

**Fig. 13:**
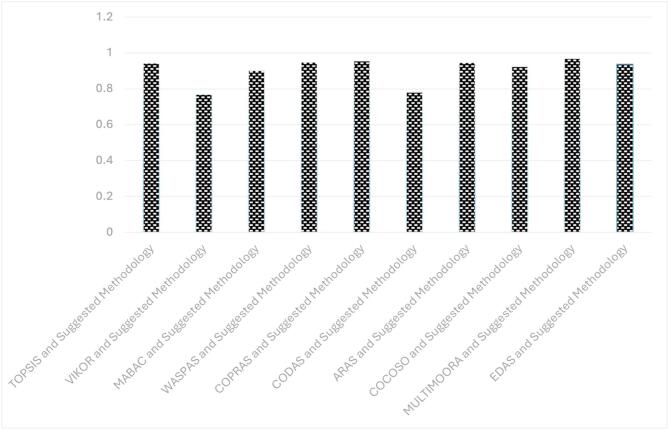
The correlation coefficient between the suggested methodology and the other ten comparative methods.

To assess the strength of association between the proposed methodology and the comparative methods, Spearman’s rank correlation coefficient was applied. This statistical measure evaluates the degree of correlation between the ranking results of the proposed methodology and those of the ten comparative MCDM methods. The coefficient may yield positive, zero, or negative values. A negative value suggests an inverse relationship between two methods, a value of zero indicates no relationship, and a positive value reflects a direct correlation. The correlation coefficients between the proposed methodology and the ten comparative methods were calculated, as illustrated in Fig. 13. All coefficients exceeded 0.77, reflecting a strong positive association between the proposed methodology and the other MCDM methods. This high level of correlation reinforces the effectiveness and reliability of the results produced by the suggested approach.

In addition to ranking consistency, the proposed SF-SWOT-CRITIC-GRA framework offers practical advantages in terms of computational efficiency and methodological simplicity. Unlike methods such as SF-TOPSIS, SF-VIKOR, or SF-MARCOS, which require additional normalization steps, distance calculations, or compromise-based solutions, SF-GRA employs a direct comparison to a reference series, resulting in lower algorithmic complexity. Furthermore, the use of the SF-CRITIC method for criteria weighting eliminates the need for subjective pairwise comparisons, as required in AHP or BWM, thereby reducing expert burden and potential bias. These features make the proposed approach well-suited for large-scale sustainability assessments where transparency, objectivity, and ease of implementation are critical. While exact runtime values are not provided due to environmental variability, the qualitative advantages demonstrate the efficiency and applicability of the model across diverse decision-making contexts.

## Analytical insights and strategic implications

This section presents the outcomes obtained from the proposed methodology SF-SWOT-CRITIC-GRA applied in “Application”. The discussion is organized into three components: the identification of the main and sub-criteria through SWOT analysis, the calculation of the weights of key and sub-factors using the SF-CRITIC method, and the ranking of the alternatives using the SF-GRA method to identify the best-performing gold mine for conversion into a green mine.

### Identification of criteria using SWOT analysis

In the first component, the SWOT analysis method was employed to identify the evaluation criteria derived from the literature. The framework comprises four main criteria: strengths, opportunities, weaknesses, and threats. These main criteria were further decomposed into 37 sub-criteria, including 10 strengths, 11 opportunities, 6 weaknesses, and 10 threats.

### Weight calculation using SF-CRITIC

In the second component, the SF-CRITIC approach was utilized to calculate the weights of both the main and sub-criteria. A panel of five experts was invited to evaluate the main and sub-criteria across 20 alternatives. To capture the uncertainty and hesitation in expert opinions, spherical fuzzy numbers (SFNs) were employed. The results of the weight calculation for the main criteria revealed that Strengths had the highest weight (0.267), Opportunities followed closely with a weight of 0.256, Threats had a weight of 0.247, and Weaknesses had the lowest weight (0.228).

These findings suggest that the threats criterion holds greater importance than weaknesses. Consequently, mining companies should place more emphasis on threat-related factors when evaluating gold mines in Egypt.

Following this, the weights for the sub-factors under each main criterion were calculated.

#### Strengths

The judgment matrix between the sub-criteria of strengths and the 20 alternatives showed that STR6 (Implementation of the Land Reclamation Program) had the highest weight, followed by STR5 (Resource Environmental Monitoring and Restoration), and STR7 (Renewable Energy Adoption). The sub-criterion with the lowest weight in the strengths category was STR4 (Corporate Management).

#### Opportunities

The judgment matrix between the sub-criteria of opportunities and the 20 alternatives indicated that OPP9 (Resource Mining and Technology Innovation) had the highest weight, followed by OPP6 (Emission Reduction), and OPP11 (Utilization Rate of Co-associated Components of the Mine). The sub-criterion with the least weight in the opportunities category was OPP2 (Government Incentives).

#### Weaknesses

The judgment matrix between the sub-criteria of weaknesses and the 20 alternatives showed that WEA5 (Energy Consumption) had the highest weight, followed by WEA6 (Water Consumption), and WEA4 (Compliance Risks Environmental). The sub-criterion with the least weight in the weaknesses category was WEA2 (Rate of Wastewater).

#### Threats

The judgment matrix between the sub-criteria of threats and the 20 alternatives revealed that THR9 (Challenges of Financial Viability of Green mine Operations) had the highest weight, followed by THR8 (COD Emissions), and THR6 (SO2 Emissions). The sub-criterion with the lowest weight in the threats category was THR2 (Market Risk).

### Ranking alternatives using SF-GRA

In the third phase, the SF-GRA method was then applied to rank the 20 alternatives based on the 37 sub-criteria. A comprehensive decision matrix was constructed, incorporating expert evaluations expressed in spherical fuzzy numbers. The final ranking revealed that GME20 emerged as the best-performing alternative. It was followed by GME1, GME19, and GME9 in descending order. Conversely, GME5 was identified as the lowest-ranked alternative.

### Managerial implications

The ranking of gold mines and the selection of the best candidate for conversion into a green mine have several important managerial implications, including:

- Mining companies should analyze key factors such as production output, operational costs, and the tools used in the mining process. This analysis can help identify areas for improvement, enabling companies to adopt more efficient mining methods that enhance overall operational effectiveness.
- Companies must assess various resources, including energy consumption, water usage, and waste management practices. By addressing these factors, mining operations can achieve significant improvements in energy efficiency, reduce operational costs, and optimize waste management, which in turn can contribute to more sustainable mining practices.
- Mining companies must prioritize the identification and reduction of emissions, waste, and energy consumption. Effective strategies for controlling emissions and reducing waste not only help in saving energy but also minimize the environmental impacts associated with mining activities. This can result in cleaner, more sustainable operations while also meeting regulatory and environmental standards.

These managerial insights highlight the importance for mining companies to adopt sustainable practices that improve both operational performance and environmental care.

### Theoretical contribution

This study proposes a novel hybrid Multi-Criteria Decision-Making (MCDM) methodology that integrates spherical fuzzy sets (SFSs) with SWOT analysis to assess the performance of gold mines in Egypt and select the best candidate for conversion into a green mine. The use of SFSs addresses the uncertainty and imprecision inherent in decision-making, while the combination of SFSs with the CRITIC and GRA methods enhances the accuracy and reliability of the decision-making process. The theoretical contributions of this study are as follows.

The proposed methodology utilizes SFNs to handle uncertainty, hesitation, and imprecise information, which are common in complex decision-making processes. The strength of SFNs lies in their ability to model and resolve ambiguous data, making the methodology suitable for a wide range of industrial problems, not limited to the green mine sector. Experts, decision-makers, and practitioners can apply this approach across various domains where uncertainty and vague information play a role in decision-making.

By combining the SFSs framework with the CRITIC method, this study offers a robust approach for calculating criteria weights in MCDM problems. The mathematical formulation used in the SFSs-CRITIC method helps reduce the influence of subjective biases and evaluations from experts or decision-makers. This enables a more objective and accurate assessment of the criteria, enhancing the credibility of the decision-making process.

This research introduces a hybrid methodology that combines SWOT analysis with SFSs, CRITIC, and GRA methods to rank alternatives (in this case, gold mines) and select the best option for conversion into a green mine. The integration of these methods provides a comprehensive approach to decision-making, addressing both internal and external factors through the SWOT framework, and enhancing the reliability of rankings with the help of SFNs, CRITIC, and GRA.

The proposed approach is validated through sensitivity and comparative analysis, which demonstrates the stability of the results and the effectiveness of the approach. By comparing the proposed methodology with other MCDM methods, the study proves the superiority and robustness of the suggested framework in handling complex, real-world decision-making problems.

### Policy insights

This study addresses gaps in the existing literature by proposing a novel methodology for assessing and ranking green mines, specifically focusing on gold mines in Egypt. The primary aim of this research is to provide resource policy authorities with a robust framework to assess the performance of green mines and identify the most suitable candidates for conversion into green mines.

The key issue tackled in this study lies in the lack of comprehensive criteria for evaluating the performance of green mines in the mining industry. This gap is addressed by compiling a set of relevant criteria through a thorough literature review, supplemented by the insights and opinions of experts and decision-makers in the field. By incorporating expert knowledge, the study ensures that the identified criteria are not only relevant but also reflective of the real-world conditions faced by the mining sector.

In addition to identifying the criteria, the study introduces a methodology to calculate the weights of these criteria, allowing policymakers to objectively assess the performance of gold mines in Egypt. The methodology supports the decision-making process by enabling policymakers to rank the mines based on their potential for sustainable practices and select the best candidate for conversion into a green mine.

Another significant contribution of this study is its approach to minimizing the subjectivity and bias inherent in expert and decision-maker opinions. While expert judgments play a crucial role in MCDM methods, they can sometimes introduce personal biases that may affect the objectivity of the decision-making process. To mitigate this, the study provides a systematic approach to calculating the weights of the experts’ opinions, using mathematical models to rank them and reduce the impact of individual biases. This empirical approach ensures that the criteria used to assess the mines are based on objective, data-driven insights rather than subjective judgments alone. By providing a more transparent and methodologically sound approach to evaluating green mines, this study offers valuable insights for policymakers in the mining sector. The proposed approach not only helps in selecting the best gold mine for conversion to a green mine but also supports broader efforts to improve sustainability in the mining industry.

## Conclusions and future works

This study has developed a comprehensive and structured decision-making framework for evaluating the green performance of gold mines in Egypt. It aims to identify the most suitable candidate for green conversion based on a broad set of sustainability-related factors. The analysis includes 20 gold mines evaluated against 37 criteria organized under the SWOT framework (strengths, weaknesses, opportunities, and threats). The proposed methodology was developed in four structured phases. First, the evaluation criteria were identified based on expert input, literature review, and the SWOT framework. Second, expert judgments were collected and expressed using spherical fuzzy linguistic terms to address uncertainty and subjectivity. Third, the SF-CRITIC method was employed to derive objective weights for the criteria, accounting for both their variability and interdependence. Finally, the SF-GRA method was applied to rank the gold mine alternatives, culminating in the selection of the most suitable mine for green transformation. While the proposed methodology is comprehensive, its computational structure remains efficient. The SF-CRITIC and SF-GRA methods used in the framework rely on direct mathematical formulations without iterative optimization, which limits computational complexity. As the number of criteria, alternatives, or experts increases, the size of the decision matrices and the number of operations grow linearly. However, the modular nature of the model allows parallel computation of fuzzy aggregations and rankings, making it scalable to larger datasets. These features make the framework suitable for practical applications in real-time decision-making or policy evaluation involving extensive data.

Additionally, sensitivity analysis is performed to test the stability of the results. The analysis is conducted in two parts: first, by varying the Grey relational parameter in the GRA method (values ranging from 0.1 to 1), and second, by adjusting the weights of the 37 criteria in 37 different scenarios. In both cases, the results remain stable, confirming the robustness of the proposed methodology. In the comparative analysis, the proposed methodology is compared with other MCDM methods such as SF-TOPSIS, SF-VIKOR, SF-EDAS, SF-MABAC, and SF-COPRAS. The results demonstrate a strong correlation (greater than 0.77) between the suggested methodology and other MCDM methods, indicating that the proposed approach is effective and reliable.

Different MCDM methods can be applied in the future studies as in^50–53^. The findings of this study open several avenues for future research on sustainable mine evaluation and decision-making, particularly in areas such as the application of alternative fuzzy logic frameworks and the extension to diverse mining contexts. Future research could explore advanced uncertainty modeling by adopting Neutrosophic sets, picture fuzzy sets, or Pythagorean fuzzy sets, which may capture more nuanced forms of expert hesitation and vagueness. Additionally, while the CRITIC method was chosen for its objectivity and computational simplicity, alternative weighting techniques such as the Analytic Hierarchy Process (AHP) or the Best-Worst Method (BWM) could be investigated for their comparative insights, especially in contexts where expert consensus is difficult to establish. The current methodology may also be extended beyond gold mines to assess the sustainability potential of other mining sectors, including coal, copper, or iron. However, this would require careful adaptation of the criteria to reflect sector-specific operational characteristics, environmental impacts, and legal requirements. Adjustments in expert linguistic evaluation and data availability could also pose practical challenges that warrant further exploration. Furthermore, the policy implications of the framework warrant deeper investigation, especially in emerging economies where regulatory systems are evolving and the strategic prioritization of sustainable mining practices is critical. Collectively, these directions reflect the adaptability of the proposed SF-SWOT-CRITIC-GRA framework and underscore its potential to guide more informed and context-sensitive decisions in the mining industry.

## Supplementary Information

Below is the link to the electronic supplementary material.

Supplementary Material 1

## Data Availability

The results, data, and figures in this manuscript have not been previously published, nor are they under consideration by another publisher. The data supporting the findings of this research are available from the corresponding author upon reasonable request.

## Material availability

All materials used in this research are the property of the authors, and no permissions are required.
