# Supplementary material for: A robust framework for evaluating green mines towards sustainable development
Source: Sci Rep. 2025 Oct 29;15:37739. doi: 10.1038/s41598-025-21237-6 (PMC12572287; doi:10.1038/s41598-025-21237-6)
Supplement: Supplementary file 1 — Supplementary Material 1 [file 41598_2025_21237_MOESM1_ESM.pdf]

Appendix

Table A1. The decision matrix between overall criteria and alternatives by the GRA method for first experts.

	GME1	GME2	GME3	GME4	GME5	GME6	GME7	GME8	GME9	GME10	GME11	GME12	GME13	GME14	GME15	GME16	GME17	GME18	GME19	GME20
STRA1	(0.9, 0.1, 0.1)	(0.8, 0.2, 0.2)	(0.9, 0.1, 0.1)	(0.4, 0.6, 0.4)	(0.3, 0.7, 0.3)	(0.8, 0.2, 0.2)	(0.4, 0.6, 0.4)	(0.4, 0.6, 0.4)	(0.3, 0.7, 0.3)	(0.8, 0.2, 0.2)	(0.9, 0.1, 0.1)	(0.4, 0.6, 0.4)	(0.3, 0.7, 0.3)	(0.4, 0.6, 0.4)	(0.8, 0.2, 0.2)	(0.9, 0.1, 0.1)	(0.4, 0.6, 0.4)	(0.3, 0.7, 0.3)	(0.8, 0.2, 0.2)	(0.9, 0.1, 0.1)
STRA2	(0.8, 0.2, 0.2)	(0.9, 0.1, 0.1)	(0.4, 0.6, 0.4)	(0.3, 0.7, 0.3)	(0.2, 0.8, 0.2)	(0.4, 0.6, 0.4)	(0.8, 0.2, 0.2)	(0.4, 0.6, 0.4)	(0.1, 0.9, 0.1)	(0.4, 0.6, 0.4)	(0.9, 0.1, 0.1)	(0.2, 0.8, 0.2)	(0.4, 0.6, 0.4)	(0.8, 0.2, 0.2)	(0.3, 0.7, 0.3)	(0.6, 0.4, 0.4)	(0.4, 0.6, 0.4)	(0.4, 0.6, 0.4)	(0.3, 0.7, 0.3)	(0.8, 0.2, 0.2)
STRA3	(0.8, 0.2, 0.2)	(0.9, 0.1, 0.1)	(0.4, 0.6, 0.4)	(0.3, 0.7, 0.3)	(0.6, 0.4, 0.4)	(0.3, 0.7, 0.3)	(0.8, 0.2, 0.2)	(0.3, 0.7, 0.3)	(0.2, 0.8, 0.2)	(0.8, 0.2, 0.2)	(0.6, 0.4, 0.4)	(0.8, 0.2, 0.2)	(0.1, 0.9, 0.1)	(0.2, 0.8, 0.2)	(0.8, 0.2, 0.2)	(0.9, 0.1, 0.1)	(0.4, 0.6, 0.4)	(0.3, 0.7, 0.3)	(0.4, 0.6, 0.4)	(0.8, 0.2, 0.2)
STRA4	(0.9, 0.1, 0.1)	(0.8, 0.2, 0.2)	(0.4, 0.6, 0.4)	(0.4, 0.6, 0.4)	(0.3, 0.7, 0.3)	(0.8, 0.2, 0.2)	(0.9, 0.1, 0.1)	(0.4, 0.6, 0.4)	(0.3, 0.7, 0.3)	(0.4, 0.6, 0.4)	(0.4, 0.6, 0.4)	(0.3, 0.7, 0.3)	(0.8, 0.2, 0.2)	(0.6, 0.4, 0.4)	(0.4, 0.6, 0.4)	(0.8, 0.2, 0.2)	(0.4, 0.6, 0.4)	(0.4, 0.6, 0.4)	(0.3, 0.7, 0.3)	(0.9, 0.1, 0.1)
STRA5	(0.1, 0.9, 0.1)	(0.8, 0.2, 0.2)	(0.4, 0.6, 0.4)	(0.4, 0.6, 0.4)	(0.4, 0.6, 0.4)	(0.8, 0.2, 0.2)	(0.9, 0.1, 0.1)	(0.4, 0.6, 0.4)	(0.3, 0.7, 0.3)	(0.8, 0.2, 0.2)	(0.6, 0.4, 0.4)	(0.9, 0.1, 0.1)	(0.4, 0.6, 0.4)	(0.8, 0.2, 0.2)	(0.4, 0.6, 0.4)	(0.4, 0.6, 0.4)	(0.3, 0.7, 0.3)	(0.8, 0.2, 0.2)	(0.9, 0.1, 0.1)	(0.1, 0.9, 0.1)
STRA6	(0.8, 0.2, 0.2)	(0.9, 0.1, 0.1)	(0.4, 0.6, 0.4)	(0.3, 0.7, 0.3)	(0.4, 0.6, 0.4)	(0.8, 0.2, 0.2)	(0.9, 0.1, 0.1)	(0.4, 0.6, 0.4)	(0.3, 0.7, 0.3)	(0.4, 0.6, 0.4)	(0.1, 0.9, 0.1)	(0.9, 0.1, 0.1)	(0.8, 0.2, 0.2)	(0.4, 0.6, 0.4)	(0.4, 0.6, 0.4)	(0.3, 0.7, 0.3)	(0.8, 0.2, 0.2)	(0.6, 0.4, 0.4)	(0.9, 0.1, 0.1)	(0.8, 0.2, 0.2)
STRA7	(0.8, 0.2, 0.2)	(0.4, 0.6, 0.4)	(0.4, 0.6, 0.4)	(0.3, 0.7, 0.3)	(0.4, 0.6, 0.4)	(0.4, 0.6, 0.4)	(0.3, 0.7, 0.3)	(0.8, 0.2, 0.2)	(0.8, 0.2, 0.2)	(0.6, 0.4, 0.4)	(0.9, 0.1, 0.1)	(0.4, 0.6, 0.4)	(0.9, 0.1, 0.1)	(0.8, 0.2, 0.2)	(0.9, 0.1, 0.1)	(0.8, 0.2, 0.2)	(0.4, 0.6, 0.4)	(0.4, 0.6, 0.4)	(0.3, 0.7, 0.3)	(0.8, 0.2, 0.2)
STRA8	(0.8, 0.2, 0.2)	(0.8, 0.2, 0.2)	(0.4, 0.6, 0.4)	(0.4, 0.6, 0.4)	(0.3, 0.7, 0.3)	(0.8, 0.2, 0.2)	(0.3, 0.7, 0.3)	(0.6, 0.4, 0.4)	(0.8, 0.2, 0.2)	(0.3, 0.7, 0.3)	(0.7, 0.3, 0.3)	(0.8, 0.2, 0.2)	(0.3, 0.7, 0.3)	(0.6, 0.4, 0.4)	(0.4, 0.6, 0.4)	(0.4, 0.6, 0.4)	(0.3, 0.7, 0.3)	(0.8, 0.2, 0.2)	(0.8, 0.2, 0.2)	(0.8, 0.2, 0.2)
STRA9	(0.4, 0.6, 0.4)	(0.4, 0.6, 0.4)	(0.3, 0.7, 0.3)	(0.8, 0.2, 0.2)	(0.9, 0.1, 0.1)	(0.8, 0.2, 0.2)	(0.3, 0.7, 0.3)	(0.6, 0.4, 0.4)	(0.8, 0.2, 0.2)	(0.6, 0.4, 0.4)	(0.9, 0.1, 0.1)	(0.4, 0.6, 0.4)	(0.9, 0.1, 0.1)	(0.8, 0.2, 0.2)	(0.6, 0.4, 0.4)	(0.4, 0.6, 0.4)	(0.4, 0.6, 0.4)	(0.3, 0.7, 0.3)	(0.8, 0.2, 0.2)	(0.4, 0.6, 0.4)
STRA10	(0.8, 0.2, 0.2)	(0.8, 0.2, 0.2)	(0.9, 0.1, 0.1)	(0.4, 0.6, 0.4)	(0.3, 0.7, 0.3)	(0.8, 0.2, 0.2)	(0.9, 0.1, 0.1)	(0.8, 0.2, 0.2)	(0.8, 0.2, 0.2)	(0.9, 0.1, 0.1)	(0.4, 0.6, 0.4)	(0.3, 0.7, 0.3)	(0.3, 0.7, 0.3)	(0.6, 0.4, 0.4)	(0.8, 0.2, 0.2)	(0.9, 0.1, 0.1)	(0.4, 0.6, 0.4)	(0.3, 0.7, 0.3)	(0.3, 0.7, 0.3)	(0.8, 0.2, 0.2)
OPP1	(0.8, 0.2, 0.2)	(0.9, 0.1, 0.1)	(0.4, 0.6, 0.4)	(0.3, 0.7, 0.3)	(0.3, 0.7, 0.3)	(0.8, 0.2, 0.2)	(0.9, 0.1, 0.1)	(0.4, 0.6, 0.4)	(0.3, 0.7, 0.3)	(0.8, 0.2, 0.2)	(0.1, 0.9, 0.1)	(0.8, 0.2, 0.2)	(0.9, 0.1, 0.1)	(0.1, 0.9, 0.1)	(0.4, 0.6, 0.4)	(0.4, 0.6, 0.4)	(0.3, 0.7, 0.3)	(0.8, 0.2, 0.2)	(0.3, 0.7, 0.3)	(0.8, 0.2, 0.2)
OPP2	(0.8, 0.2, 0.2)	(0.9, 0.1, 0.1)	(0.4, 0.6, 0.4)	(0.3, 0.7, 0.3)	(0.6, 0.4, 0.4)	(0.8, 0.2, 0.2)	(0.9, 0.1, 0.1)	(0.4, 0.6, 0.4)	(0.3, 0.7, 0.3)	(0.4, 0.6, 0.4)	(0.4, 0.6, 0.4)	(0.8, 0.2, 0.2)	(0.1, 0.9, 0.1)	(0.2, 0.8, 0.2)	(0.6, 0.4, 0.4)	(0.8, 0.2, 0.2)	(0.8, 0.2, 0.2)	(0.1, 0.9, 0.1)	(0.2, 0.8, 0.2)	(0.8, 0.2, 0.2)
OPP3	(0.8, 0.2, 0.2)	(0.9, 0.1, 0.1)	(0.4, 0.6, 0.4)	(0.3, 0.7, 0.3)	(0.8, 0.2, 0.2)	(0.9, 0.1, 0.1)	(0.4, 0.6, 0.4)	(0.3, 0.7, 0.3)	(0.6, 0.4, 0.4)	(0.7, 0.3, 0.3)	(0.4, 0.6, 0.4)	(0.8, 0.2, 0.2)	(0.7, 0.3, 0.3)	(0.4, 0.6, 0.4)	(0.8, 0.2, 0.2)	(0.9, 0.1, 0.1)	(0.4, 0.6, 0.4)	(0.3, 0.7, 0.3)	(0.8, 0.2, 0.2)	(0.8, 0.2, 0.2)
OPP4	(0.9, 0.1, 0.1)	(0.8, 0.2, 0.2)	(0.9, 0.1, 0.1)	(0.4, 0.6, 0.4)	(0.3, 0.7, 0.3)	(0.4, 0.6, 0.4)	(0.3, 0.7, 0.3)	(0.4, 0.6, 0.4)	(0.3, 0.7, 0.3)	(0.4, 0.6, 0.4)	(0.6, 0.4, 0.4)	(0.7, 0.3, 0.3)	(0.8, 0.2, 0.2)	(0.9, 0.1, 0.1)	(0.4, 0.6, 0.4)	(0.3, 0.7, 0.3)	(0.6, 0.4, 0.4)	(0.7, 0.3, 0.3)	(0.4, 0.6, 0.4)	(0.9, 0.1, 0.1)
OPP5	(0.6, 0.4, 0.4)	(0.7, 0.3, 0.3)	(0.4, 0.6, 0.4)	(0.8, 0.2, 0.2)	(0.6, 0.4, 0.4)	(0.7, 0.3, 0.3)	(0.4, 0.6, 0.4)	(0.6, 0.4, 0.4)	(0.7, 0.3, 0.3)	(0.4, 0.6, 0.4)	(0.6, 0.4, 0.4)	(0.7, 0.3, 0.3)	(0.4, 0.6, 0.4)	(0.8, 0.2, 0.2)	(0.6, 0.4, 0.4)	(0.7, 0.3, 0.3)	(0.4, 0.6, 0.4)	(0.8, 0.2, 0.2)	(0.4, 0.6, 0.4)	(0.6, 0.4, 0.4)
OPP6	(0.4, 0.6, 0.4)	(0.7, 0.3, 0.3)	(0.4, 0.6, 0.4)	(0.8, 0.2, 0.2)	(0.4, 0.6, 0.4)	(0.8, 0.2, 0.2)	(0.4, 0.6, 0.4)	(0.6, 0.4, 0.4)	(0.6, 0.4, 0.4)	(0.7, 0.3, 0.3)	(0.6, 0.4, 0.4)	(0.7, 0.3, 0.3)	(0.4, 0.6, 0.4)	(0.8, 0.2, 0.2)	(0.4, 0.6, 0.4)	(0.6, 0.4, 0.4)	(0.7, 0.3, 0.3)	(0.4, 0.6, 0.4)	(0.8, 0.2, 0.2)	(0.4, 0.6, 0.4)
OPP7	(0.3, 0.7, 0.3)	(0.6, 0.4, 0.4)	(0.7, 0.3, 0.3)	(0.4, 0.6, 0.4)	(0.8, 0.2, 0.2)	(0.4, 0.6, 0.4)	(0.3, 0.7, 0.3)	(0.6, 0.4, 0.4)	(0.7, 0.3, 0.3)	(0.4, 0.6, 0.4)	(0.8, 0.2, 0.2)	(0.9, 0.1, 0.1)	(0.4, 0.6, 0.4)	(0.8, 0.2, 0.2)	(0.6, 0.4, 0.4)	(0.7, 0.3, 0.3)	(0.4, 0.6, 0.4)	(0.8, 0.2, 0.2)	(0.8, 0.2, 0.2)	(0.3, 0.7, 0.3)
OPP8	(0.6, 0.4, 0.4)	(0.7, 0.3, 0.3)	(0.4, 0.6, 0.4)	(0.8, 0.2, 0.2)	(0.8, 0.2, 0.2)	(0.9, 0.1, 0.1)	(0.8, 0.2, 0.2)	(0.6, 0.4, 0.4)	(0.9, 0.1, 0.1)	(0.4, 0.6, 0.4)	(0.1, 0.9, 0.1)	(0.9, 0.1, 0.1)	(0.6, 0.4, 0.4)	(0.7, 0.3, 0.3)	(0.4, 0.6, 0.4)	(0.8, 0.2, 0.2)	(0.7, 0.3, 0.3)	(0.4, 0.6, 0.4)	(0.8, 0.2, 0.2)	(0.6, 0.4, 0.4)
OPP9	(0.6, 0.4, 0.4)	(0.7, 0.3, 0.3)	(0.4, 0.6, 0.4)	(0.8, 0.2, 0.2)	(0.6, 0.4, 0.4)	(0.7, 0.3, 0.3)	(0.4, 0.6, 0.4)	(0.6, 0.4, 0.4)	(0.6, 0.4, 0.4)	(0.7, 0.3, 0.3)	(0.4, 0.6, 0.4)	(0.8, 0.2, 0.2)	(0.9, 0.1, 0.1)	(0.8, 0.2, 0.2)	(0.9, 0.1, 0.1)	(0.6, 0.4, 0.4)	(0.7, 0.3, 0.3)	(0.4, 0.6, 0.4)	(0.8, 0.2, 0.2)	(0.6, 0.4, 0.4)
OPP10	(0.8, 0.2, 0.2)	(0.6, 0.4, 0.4)	(0.6, 0.4, 0.4)	(0.7, 0.3, 0.3)	(0.4, 0.6, 0.4)	(0.8, 0.2, 0.2)	(0.3, 0.7, 0.3)	(0.6, 0.4, 0.4)	(0.7, 0.3, 0.3)	(0.4, 0.6, 0.4)	(0.6, 0.4, 0.4)	(0.7, 0.3, 0.3)	(0.4, 0.6, 0.4)	(0.8, 0.2, 0.2)	(0.4, 0.6, 0.4)	(0.4, 0.6, 0.4)	(0.3, 0.7, 0.3)	(0.8, 0.2, 0.2)	(0.8, 0.2, 0.2)	(0.8, 0.2, 0.2)
OPP11	(0.4, 0.6, 0.4)	(0.6, 0.4, 0.4)	(0.7, 0.3, 0.3)	(0.4, 0.6, 0.4)	(0.8, 0.2, 0.2)	(0.6, 0.4, 0.4)	(0.7, 0.3, 0.3)	(0.6, 0.4, 0.4)	(0.7, 0.3, 0.3)	(0.8, 0.2, 0.2)	(0.9, 0.1, 0.1)	(0.4, 0.6, 0.4)	(0.3, 0.7, 0.3)	(0.8, 0.2, 0.2)	(0.8, 0.2, 0.2)	(0.9, 0.1, 0.1)	(0.4, 0.6, 0.4)	(0.3, 0.7, 0.3)	(0.8, 0.2, 0.2)	(0.4, 0.6, 0.4)
WEA1	(0.6, 0.4, 0.4)	(0.7, 0.3, 0.3)	(0.4, 0.6, 0.4)	(0.6, 0.4, 0.4)	(0.7, 0.3, 0.3)	(0.6, 0.4, 0.4)	(0.7, 0.3, 0.3)	(0.4, 0.6, 0.4)	(0.8, 0.2, 0.2)	(0.4, 0.6, 0.4)	(0.3, 0.7, 0.3)	(0.8, 0.2, 0.2)	(0.3, 0.7, 0.3)	(0.6, 0.4, 0.4)	(0.4, 0.6, 0.4)	(0.4, 0.6, 0.4)	(0.3, 0.7, 0.3)	(0.8, 0.2, 0.2)	(0.3, 0.7, 0.3)	(0.6, 0.4, 0.4)
WEA2	(0.6, 0.4, 0.4)	(0.8, 0.2, 0.2)	(0.9, 0.1, 0.1)	(0.4, 0.6, 0.4)	(0.3, 0.7, 0.3)	(0.4, 0.6, 0.4)	(0.8, 0.2, 0.2)	(0.9, 0.1, 0.1)	(0.1, 0.9, 0.1)	(0.8, 0.2, 0.2)	(0.1, 0.9, 0.1)	(0.8, 0.2, 0.2)	(0.9, 0.1, 0.1)	(0.1, 0.9, 0.1)	(0.8, 0.2, 0.2)	(0.9, 0.1, 0.1)	(0.4, 0.6, 0.4)	(0.3, 0.7, 0.3)	(0.3, 0.7, 0.3)	(0.6, 0.4, 0.4)
WEA3	(0.8, 0.2, 0.2)	(0.1, 0.9, 0.1)	(0.2, 0.8, 0.2)	(0.6, 0.4, 0.4)	(0.6, 0.4, 0.4)	(0.3, 0.7, 0.3)	(0.8, 0.2, 0.2)	(0.8, 0.2, 0.2)	(0.9, 0.1, 0.1)	(0.4, 0.6, 0.4)	(0.3, 0.7, 0.3)	(0.8, 0.2, 0.2)	(0.1, 0.9, 0.1)	(0.2, 0.8, 0.2)	(0.6, 0.4, 0.4)	(0.8, 0.2, 0.2)	(0.9, 0.1, 0.1)	(0.4, 0.6, 0.4)	(0.3, 0.7, 0.3)	(0.8, 0.2, 0.2)
WEA4	(0.9, 0.1, 0.1)	(0.8, 0.2, 0.2)	(0.9, 0.1, 0.1)	(0.4, 0.6, 0.4)	(0.8, 0.2, 0.2)	(0.9, 0.1, 0.1)	(0.4, 0.6, 0.4)	(0.3, 0.7, 0.3)	(0.9, 0.1, 0.1)	(0.4, 0.6, 0.4)	(0.7, 0.3, 0.3)	(0.8, 0.2, 0.2)	(0.9, 0.1, 0.1)	(0.4, 0.6, 0.4)	(0.3, 0.7, 0.3)	(0.4, 0.6, 0.4)	(0.7, 0.3, 0.3)	(0.9, 0.1, 0.1)	(0.4, 0.6, 0.4)	(0.9, 0.1, 0.1)
WEA5	(0.9, 0.1, 0.1)	(0.6, 0.4, 0.4)	(0.9, 0.1, 0.1)	(0.4, 0.6, 0.4)	(0.7, 0.3, 0.3)	(0.9, 0.1, 0.1)	(0.8, 0.2, 0.2)	(0.9, 0.1, 0.1)	(0.4, 0.6, 0.4)	(0.3, 0.7, 0.3)	(0.9, 0.1, 0.1)	(0.2, 0.8, 0.2)	(0.4, 0.6, 0.4)	(0.8, 0.2, 0.2)	(0.3, 0.7, 0.3)	(0.6, 0.4, 0.4)	(0.4, 0.6, 0.4)	(0.4, 0.6, 0.4)	(0.3, 0.7, 0.3)	(0.9, 0.1, 0.1)
WEA6	(0.8, 0.2, 0.2)	(0.4, 0.6, 0.4)	(0.7, 0.3, 0.3)	(0.9, 0.1, 0.1)	(0.4, 0.6, 0.4)	(0.7, 0.3, 0.3)	(0.4, 0.6, 0.4)	(0.7, 0.3, 0.3)	(0.4, 0.6, 0.4)	(0.7, 0.3, 0.3)	(0.9, 0.1, 0.1)	(0.4, 0.6, 0.4)	(0.7, 0.3, 0.3)	(0.2, 0.8, 0.2)	(0.4, 0.6, 0.4)	(0.7, 0.3, 0.3)	(0.4, 0.6, 0.4)	(0.7, 0.3, 0.3)	(0.9, 0.1, 0.1)	(0.8, 0.2, 0.2)
THR1	(0.9, 0.1, 0.1)	(0.4, 0.6, 0.4)	(0.7, 0.3, 0.3)	(0.9, 0.1, 0.1)	(0.4, 0.6, 0.4)	(0.7, 0.3, 0.3)	(0.4, 0.6, 0.4)	(0.3, 0.7, 0.3)	(0.8, 0.2, 0.2)	(0.4, 0.6, 0.4)	(0.4, 0.6, 0.4)	(0.3, 0.7, 0.3)	(0.8, 0.2, 0.2)	(0.6, 0.4, 0.4)	(0.4, 0.6, 0.4)	(0.8, 0.2, 0.2)	(0.4, 0.6, 0.4)	(0.4, 0.6, 0.4)	(0.3, 0.7, 0.3)	(0.9, 0.1, 0.1)
THR2	(0.1, 0.9, 0.1)	(0.4, 0.6, 0.4)	(0.7, 0.3, 0.3)	(0.9, 0.1, 0.1)	(0.4, 0.6, 0.4)	(0.7, 0.3, 0.3)	(0.4, 0.6, 0.4)	(0.7, 0.3, 0.3)	(0.4, 0.6, 0.4)	(0.7, 0.3, 0.3)	(0.9, 0.1, 0.1)	(0.4, 0.6, 0.4)	(0.7, 0.3, 0.3)	(0.8, 0.2, 0.2)	(0.4, 0.6, 0.4)	(0.7, 0.3, 0.3)	(0.9, 0.1, 0.1)	(0.4, 0.6, 0.4)	(0.7, 0.3, 0.3)	(0.1, 0.9, 0.1)
THR3	(0.9, 0.1, 0.1)	(0.8, 0.2, 0.2)	(0.6, 0.4, 0.4)	(0.9, 0.1, 0.1)	(0.4, 0.6, 0.4)	(0.9, 0.1, 0.1)	(0.8, 0.2, 0.2)	(0.6, 0.4, 0.4)	(0.9, 0.1, 0.1)	(0.4, 0.6, 0.4)	(0.1, 0.9, 0.1)	(0.9, 0.1, 0.1)	(0.8, 0.2, 0.2)	(0.4, 0.6, 0.4)	(0.4, 0.6, 0.4)	(0.3, 0.7, 0.3)	(0.8, 0.2, 0.2)	(0.6, 0.4, 0.4)	(0.9, 0.1, 0.1)	(0.9, 0.1, 0.1)
THR4	(0.8, 0.2, 0.2)	(0.4, 0.6, 0.4)	(0.7, 0.3, 0.3)	(0.4, 0.6, 0.4)	(0.7, 0.3, 0.3)	(0.9, 0.1, 0.1)	(0.4, 0.6, 0.4)	(0.7, 0.3, 0.3)	(0.8, 0.2, 0.2)	(0.6, 0.4, 0.4)	(0.9, 0.1, 0.1)	(0.4, 0.6, 0.4)	(0.9, 0.1, 0.1)	(0.8, 0.2, 0.2)	(0.4, 0.6, 0.4)	(0.7, 0.3, 0.3)	(0.9, 0.1, 0.1)	(0.4, 0.6, 0.4)	(0.7, 0.3, 0.3)	(0.8, 0.2, 0.2)
THR5	(0.8, 0.2, 0.2)	(0.4, 0.6, 0.4)	(0.7, 0.3, 0.3)	(0.9, 0.1, 0.1)	(0.4, 0.6, 0.4)	(0.7, 0.3, 0.3)	(0.3, 0.7, 0.3)	(0.6, 0.4, 0.4)	(0.8, 0.2, 0.2)	(0.3, 0.7, 0.3)	(0.7, 0.3, 0.3)	(0.8, 0.2, 0.2)	(0.3, 0.7, 0.3)	(0.6, 0.4, 0.4)	(0.4, 0.6, 0.4)	(0.4, 0.6, 0.4)	(0.4, 0.6, 0.4)	(0.7, 0.3, 0.3)	(0.9, 0.1, 0.1)	(0.8, 0.2, 0.2)
THR6	(0.4, 0.6, 0.4)	(0.4, 0.6, 0.4)	(0.4, 0.6, 0.4)	(0.7, 0.3, 0.3)	(0.9, 0.1, 0.1)	(0.4, 0.6, 0.4)	(0.7, 0.3, 0.3)	(0.6, 0.4, 0.4)	(0.8, 0.2, 0.2)	(0.6, 0.4, 0.4)	(0.9, 0.1, 0.1)	(0.4, 0.6, 0.4)	(0.9, 0.1, 0.1)	(0.8, 0.2, 0.2)	(0.4, 0.6, 0.4)	(0.7, 0.3, 0.3)	(0.9, 0.1, 0.1)	(0.4, 0.6, 0.4)	(0.7, 0.3, 0.3)	(0.4, 0.6, 0.4)
THR7	(0.9, 0.1, 0.1)	(0.8, 0.2, 0.2)	(0.4, 0.6, 0.4)	(0.4, 0.6, 0.4)	(0.3, 0.7, 0.3)	(0.8, 0.2, 0.2)	(0.8, 0.2, 0.2)	(0.9, 0.1, 0.1)	(0.4, 0.6, 0.4)	(0.3, 0.7, 0.3)	(0.4, 0.6, 0.4)	(0.7, 0.3, 0.3)	(0.9, 0.1, 0.1)	(0.4, 0.6, 0.4)	(0.7, 0.3, 0.3)	(0.9, 0.1, 0.1)	(0.4, 0.6, 0.4)	(0.7, 0.3, 0.3)	(0.3, 0.7, 0.3)	(0.9, 0.1, 0.1)
THR8	(0.4, 0.6, 0.4)	(0.7, 0.3, 0.3)	(0.9, 0.1, 0.1)	(0.4, 0.6, 0.4)	(0.7, 0.3, 0.3)	(0.9, 0.1, 0.1)	(0.8, 0.2, 0.2)	(0.8, 0.2, 0.2)	(0.9, 0.1, 0.1)	(0.4, 0.6, 0.4)	(0.3, 0.7, 0.3)	(0.8, 0.2, 0.2)	(0.9, 0.1, 0.1)	(0.4, 0.6, 0.4)	(0.3, 0.7, 0.3)	(0.7, 0.3, 0.3)	(0.9, 0.1, 0.1)	(0.4, 0.6, 0.4)	(0.7, 0.3, 0.3)	(0.4, 0.6, 0.4)
THR9	(0.9, 0.1, 0.1)	(0.8, 0.2, 0.2)	(0.9, 0.1, 0.1)	(0.4, 0.6, 0.4)	(0.3, 0.7, 0.3)	(0.4, 0.6, 0.4)	(0.8, 0.2, 0.2)	(0.8, 0.2, 0.2)	(0.1, 0.9, 0.1)	(0.2, 0.8, 0.2)	(0.6, 0.4, 0.4)	(0.8, 0.2, 0.2)	(0.9, 0.1, 0.1)	(0.4, 0.6, 0.4)	(0.3, 0.7, 0.3)	(0.1, 0.9, 0.1)	(0.2, 0.8, 0.2)	(0.6, 0.4, 0.4)	(0.3, 0.7, 0.3)	(0.9, 0.1, 0.1)
THR10	(0.9, 0.1, 0.1)	(0.4, 0.6, 0.4)	(0.8, 0.2, 0.2)	(0.9, 0.1, 0.1)	(0.4, 0.6, 0.4)	(0.3, 0.7, 0.3)	(0.4, 0.6, 0.4)	(0.4, 0.6, 0.4)	(0.3, 0.7, 0.3)	(0.8, 0.2, 0.2)	(0.6, 0.4, 0.4)	(0.8, 0.2, 0.2)	(0.9, 0.1, 0.1)	(0.4, 0.6, 0.4)	(0.3, 0.7, 0.3)	(0.4, 0.6, 0.4)	(0.4, 0.6, 0.4)	(0.3, 0.7, 0.3)	(0.8, 0.2, 0.2)	(0.9, 0.1, 0.1)

Table A2. The decision matrix between overall criteria and alternatives by the GRA method for second experts.

	GME1	GME2	GME3	GME4	GME5	GME6	GME7	GME8	GME9	GME10	GME11	GME12	GME13	GME14	GME15	GME16	GME17	GME18	GME19	GME20
STRA1	(0.1, 0.9, 0.1)	(0.8, 0.2, 0.2)	(0.6, 0.4, 0.4)	(0.7, 0.3, 0.3)	(0.3, 0.7, 0.3)	(0.4, 0.6, 0.4)	(0.8, 0.2, 0.2)	(0.9, 0.1, 0.1)	(0.9, 0.1, 0.1)	(0.8, 0.2, 0.2)	(0.6, 0.4, 0.4)	(0.9, 0.1, 0.1)	(0.8, 0.2, 0.2)	(0.6, 0.4, 0.4)	(0.7, 0.3, 0.3)	(0.3, 0.7, 0.3)	(0.4, 0.6, 0.4)	(0.8, 0.2, 0.2)	(0.9, 0.1, 0.1)	(0.6, 0.4, 0.4)
STRA2	(0.9, 0.1, 0.1)	(0.8, 0.2, 0.2)	(0.6, 0.4, 0.4)	(0.8, 0.2, 0.2)	(0.1, 0.9, 0.1)	(0.2, 0.8, 0.2)	(0.6, 0.4, 0.4)	(0.6, 0.4, 0.4)	(0.9, 0.1, 0.1)	(0.4, 0.6, 0.4)	(0.1, 0.9, 0.1)	(0.9, 0.1, 0.1)	(0.8, 0.2, 0.2)	(0.6, 0.4, 0.4)	(0.7, 0.3, 0.3)	(0.3, 0.7, 0.3)	(0.4, 0.6, 0.4)	(0.8, 0.2, 0.2)	(0.9, 0.1, 0.1)	(0.4, 0.6, 0.4)
STRA3	(0.8, 0.2, 0.2)	(0.8, 0.2, 0.2)	(0.1, 0.9, 0.1)	(0.2, 0.8, 0.2)	(0.6, 0.4, 0.4)	(0.4, 0.6, 0.4)	(0.3, 0.7, 0.3)	(0.8, 0.2, 0.2)	(0.8, 0.2, 0.2)	(0.1, 0.9, 0.1)	(0.2, 0.8, 0.2)	(0.6, 0.4, 0.4)	(0.9, 0.1, 0.1)	(0.8, 0.2, 0.2)	(0.6, 0.4, 0.4)	(0.7, 0.3, 0.3)	(0.3, 0.7, 0.3)	(0.4, 0.6, 0.4)	(0.8, 0.2, 0.2)	(0.9, 0.1, 0.1)
STRA4	(0.8, 0.2, 0.2)	(0.6, 0.4, 0.4)	(0.7, 0.3, 0.3)	(0.3, 0.7, 0.3)	(0.4, 0.6, 0.4)	(0.8, 0.2, 0.2)	(0.9, 0.1, 0.1)	(0.4, 0.6, 0.4)	(0.6, 0.4, 0.4)	(0.7, 0.3, 0.3)	(0.7, 0.3, 0.3)	(0.8, 0.2, 0.2)	(0.3, 0.7, 0.3)	(0.8, 0.2, 0.2)	(0.6, 0.4, 0.4)	(0.7, 0.3, 0.3)	(0.3, 0.7, 0.3)	(0.4, 0.6, 0.4)	(0.8, 0.2, 0.2)	(0.9, 0.1, 0.1)
STRA5	(0.4, 0.6, 0.4)	(0.8, 0.2, 0.2)	(0.6, 0.4, 0.4)	(0.7, 0.3, 0.3)	(0.3, 0.7, 0.3)	(0.4, 0.6, 0.4)	(0.8, 0.2, 0.2)	(0.9, 0.1, 0.1)	(0.1, 0.9, 0.1)	(0.8, 0.2, 0.2)	(0.6, 0.4, 0.4)	(0.7, 0.3, 0.3)	(0.3, 0.7, 0.3)	(0.8, 0.2, 0.2)	(0.6, 0.4, 0.4)	(0.7, 0.3, 0.3)	(0.3, 0.7, 0.3)	(0.4, 0.6, 0.4)	(0.8, 0.2, 0.2)	(0.9, 0.1, 0.1)
STRA6	(0.8, 0.2, 0.2)	(0.6, 0.4, 0.4)	(0.7, 0.3, 0.3)	(0.3, 0.7, 0.3)	(0.4, 0.6, 0.4)	(0.8, 0.2, 0.2)	(0.9, 0.1, 0.1)	(0.8, 0.2, 0.2)	(0.6, 0.4, 0.4)	(0.7, 0.3, 0.3)	(0.3, 0.7, 0.3)	(0.4, 0.6, 0.4)	(0.8, 0.2, 0.2)	(0.9, 0.1, 0.1)	(0.6, 0.4, 0.4)	(0.7, 0.3, 0.3)	(0.3, 0.7, 0.3)	(0.4, 0.6, 0.4)	(0.8, 0.2, 0.2)	(0.9, 0.1, 0.1)
STRA7	(0.4, 0.6, 0.4)	(0.8, 0.2, 0.2)	(0.1, 0.9, 0.1)	(0.2, 0.8, 0.2)	(0.6, 0.4, 0.4)	(0.1, 0.9, 0.1)	(0.2, 0.8, 0.2)	(0.8, 0.2, 0.2)	(0.6, 0.4, 0.4)	(0.7, 0.3, 0.3)	(0.3, 0.7, 0.3)	(0.4, 0.6, 0.4)	(0.8, 0.2, 0.2)	(0.9, 0.1, 0.1)	(0.1, 0.9, 0.1)	(0.2, 0.8, 0.2)	(0.6, 0.4, 0.4)	(0.1, 0.9, 0.1)	(0.2, 0.8, 0.2)	(0.6, 0.4, 0.4)
STRA8	(0.8, 0.2, 0.2)	(0.6, 0.4, 0.4)	(0.8, 0.2, 0.2)	(0.6, 0.4, 0.4)	(0.7, 0.3, 0.3)	(0.8, 0.2, 0.2)	(0.6, 0.4, 0.4)	(0.7, 0.3, 0.3)	(0.3, 0.7, 0.3)	(0.4, 0.6, 0.4)	(0.8, 0.2, 0.2)	(0.9, 0.1, 0.1)	(0.8, 0.2, 0.2)	(0.6, 0.4, 0.4)	(0.7, 0.3, 0.3)	(0.8, 0.2, 0.2)	(0.6, 0.4, 0.4)	(0.7, 0.3, 0.3)	(0.3, 0.7, 0.3)	(0.4, 0.6, 0.4)
STRA9	(0.9, 0.1, 0.1)	(0.3, 0.7, 0.3)	(0.8, 0.2, 0.2)	(0.6, 0.4, 0.4)	(0.7, 0.3, 0.3)	(0.3, 0.7, 0.3)	(0.4, 0.6, 0.4)	(0.8, 0.2, 0.2)	(0.9, 0.1, 0.1)	(0.6, 0.4, 0.4)	(0.7, 0.3, 0.3)	(0.3, 0.7, 0.3)	(0.4, 0.6, 0.4)	(0.8, 0.2, 0.2)	(0.9, 0.1, 0.1)	(0.3, 0.7, 0.3)	(0.4, 0.6, 0.4)	(0.6, 0.4, 0.4)	(0.7, 0.3, 0.3)	(0.9, 0.1, 0.1)
STRA10	(0.9, 0.1, 0.1)	(0.9, 0.1, 0.1)	(0.8, 0.2, 0.2)	(0.6, 0.4, 0.4)	(0.7, 0.3, 0.3)	(0.3, 0.7, 0.3)	(0.4, 0.6, 0.4)	(0.8, 0.2, 0.2)	(0.9, 0.1, 0.1)	(0.4, 0.6, 0.4)	(0.9, 0.1, 0.1)	(0.3, 0.7, 0.3)	(0.4, 0.6, 0.4)	(0.8, 0.2, 0.2)	(0.6, 0.4, 0.4)	(0.7, 0.3, 0.3)	(0.3, 0.7, 0.3)	(0.4, 0.6, 0.4)	(0.8, 0.2, 0.2)	(0.9, 0.1, 0.1)
OPP1	(0.8, 0.2, 0.2)	(0.9, 0.1, 0.1)	(0.3, 0.7, 0.3)	(0.4, 0.6, 0.4)	(0.8, 0.2, 0.2)	(0.6, 0.4, 0.4)	(0.7, 0.3, 0.3)	(0.3, 0.7, 0.3)	(0.4, 0.6, 0.4)	(0.8, 0.2, 0.2)	(0.9, 0.1, 0.1)	(0.3, 0.7, 0.3)	(0.4, 0.6, 0.4)	(0.8, 0.2, 0.2)	(0.6, 0.4, 0.4)	(0.7, 0.3, 0.3)	(0.3, 0.7, 0.3)	(0.4, 0.6, 0.4)	(0.8, 0.2, 0.2)	(0.9, 0.1, 0.1)
OPP2	(0.9, 0.1, 0.1)	(0.3, 0.7, 0.3)	(0.8, 0.2, 0.2)	(0.6, 0.4, 0.4)	(0.7, 0.3, 0.3)	(0.3, 0.7, 0.3)	(0.4, 0.6, 0.4)	(0.8, 0.2, 0.2)	(0.9, 0.1, 0.1)	(0.3, 0.7, 0.3)	(0.4, 0.6, 0.4)	(0.8, 0.2, 0.2)	(0.9, 0.1, 0.1)	(0.6, 0.4, 0.4)	(0.4, 0.6, 0.4)	(0.9, 0.1, 0.1)	(0.3, 0.7, 0.3)	(0.4, 0.6, 0.4)	(0.6, 0.4, 0.4)	(0.7, 0.3, 0.3)
OPP3	(0.1, 0.9, 0.1)	(0.9, 0.1, 0.1)	(0.8, 0.2, 0.2)	(0.6, 0.4, 0.4)	(0.7, 0.3, 0.3)	(0.3, 0.7, 0.3)	(0.4, 0.6, 0.4)	(0.8, 0.2, 0.2)	(0.9, 0.1, 0.1)	(0.4, 0.6, 0.4)	(0.9, 0.1, 0.1)	(0.8, 0.2, 0.2)	(0.6, 0.4, 0.4)	(0.7, 0.3, 0.3)	(0.3, 0.7, 0.3)	(0.4, 0.6, 0.4)	(0.8, 0.2, 0.2)	(0.9, 0.1, 0.1)	(0.7, 0.3, 0.3)	(0.4, 0.6, 0.4)
OPP4	(0.9, 0.1, 0.1)	(0.8, 0.2, 0.2)	(0.8, 0.2, 0.2)	(0.6, 0.4, 0.4)	(0.7, 0.3, 0.3)	(0.3, 0.7, 0.3)	(0.4, 0.6, 0.4)	(0.8, 0.2, 0.2)	(0.9, 0.1, 0.1)	(0.6, 0.4, 0.4)	(0.7, 0.3, 0.3)	(0.3, 0.7, 0.3)	(0.4, 0.6, 0.4)	(0.8, 0.2, 0.2)	(0.9, 0.1, 0.1)	(0.3, 0.7, 0.3)	(0.4, 0.6, 0.4)	(0.6, 0.4, 0.4)	(0.7, 0.3, 0.3)	(0.7, 0.3, 0.3)
OPP5	(0.8, 0.2, 0.2)	(0.9, 0.1, 0.1)	(0.8, 0.2, 0.2)	(0.6, 0.4, 0.4)	(0.7, 0.3, 0.3)	(0.3, 0.7, 0.3)	(0.4, 0.6, 0.4)	(0.8, 0.2, 0.2)	(0.8, 0.2, 0.2)	(0.6, 0.4, 0.4)	(0.7, 0.3, 0.3)	(0.3, 0.7, 0.3)	(0.4, 0.6, 0.4)	(0.8, 0.2, 0.2)	(0.9, 0.1, 0.1)	(0.3, 0.7, 0.3)	(0.4, 0.6, 0.4)	(0.6, 0.4, 0.4)	(0.7, 0.3, 0.3)	(0.8, 0.2, 0.2)
OPP6	(0.9, 0.1, 0.1)	(0.3, 0.7, 0.3)	(0.4, 0.6, 0.4)	(0.6, 0.4, 0.4)	(0.7, 0.3, 0.3)	(0.8, 0.2, 0.2)	(0.3, 0.7, 0.3)	(0.9, 0.1, 0.1)	(0.3, 0.7, 0.3)	(0.4, 0.6, 0.4)	(0.8, 0.2, 0.2)	(0.6, 0.4, 0.4)	(0.7, 0.3, 0.3)	(0.3, 0.7, 0.3)	(0.4, 0.6, 0.4)	(0.8, 0.2, 0.2)	(0.9, 0.1, 0.1)	(0.6, 0.4, 0.4)	(0.7, 0.3, 0.3)	(0.6, 0.4, 0.4)
OPP7	(0.9, 0.1, 0.1)	(0.3, 0.7, 0.3)	(0.4, 0.6, 0.4)	(0.6, 0.4, 0.4)	(0.7, 0.3, 0.3)	(0.8, 0.2, 0.2)	(0.3, 0.7, 0.3)	(0.9, 0.1, 0.1)	(0.3, 0.7, 0.3)	(0.4, 0.6, 0.4)	(0.8, 0.2, 0.2)	(0.6, 0.4, 0.4)	(0.7, 0.3, 0.3)	(0.3, 0.7, 0.3)	(0.4, 0.6, 0.4)	(0.8, 0.2, 0.2)	(0.9, 0.1, 0.1)	(0.3, 0.7, 0.3)	(0.8, 0.2, 0.2)	(0.9, 0.1, 0.1)
OPP8	(0.9, 0.1, 0.1)	(0.3, 0.7, 0.3)	(0.4, 0.6, 0.4)	(0.6, 0.4, 0.4)	(0.7, 0.3, 0.3)	(0.9, 0.1, 0.1)	(0.3, 0.7, 0.3)	(0.4, 0.6, 0.4)	(0.6, 0.4, 0.4)	(0.7, 0.3, 0.3)	(0.3, 0.7, 0.3)	(0.8, 0.2, 0.2)	(0.3, 0.7, 0.3)	(0.6, 0.4, 0.4)	(0.9, 0.1, 0.1)	(0.3, 0.7, 0.3)	(0.4, 0.6, 0.4)	(0.6, 0.4, 0.4)	(0.7, 0.3, 0.3)	(0.1, 0.9, 0.1)
OPP9	(0.9, 0.1, 0.1)	(0.3, 0.7, 0.3)	(0.4, 0.6, 0.4)	(0.6, 0.4, 0.4)	(0.7, 0.3, 0.3)	(0.8, 0.2, 0.2)	(0.9, 0.1, 0.1)	(0.3, 0.7, 0.3)	(0.4, 0.6, 0.4)	(0.6, 0.4, 0.4)	(0.7, 0.3, 0.3)	(0.8, 0.2, 0.2)	(0.9, 0.1, 0.1)	(0.1, 0.9, 0.1)	(0.9, 0.1, 0.1)	(0.3, 0.7, 0.3)	(0.4, 0.6, 0.4)	(0.6, 0.4, 0.4)	(0.7, 0.3, 0.3)	(0.1, 0.9, 0.1)
OPP10	(0.8, 0.2, 0.2)	(0.6, 0.4, 0.4)	(0.7, 0.3, 0.3)	(0.3, 0.7, 0.3)	(0.4, 0.6, 0.4)	(0.8, 0.2, 0.2)	(0.9, 0.1, 0.1)	(0.6, 0.4, 0.4)	(0.7, 0.3, 0.3)	(0.8, 0.2, 0.2)	(0.6, 0.4, 0.4)	(0.7, 0.3, 0.3)	(0.8, 0.2, 0.2)	(0.6, 0.4, 0.4)	(0.7, 0.3, 0.3)	(0.3, 0.7, 0.3)	(0.4, 0.6, 0.4)	(0.8, 0.2, 0.2)	(0.9, 0.1, 0.1)	(0.6, 0.4, 0.4)
OPP11	(0.4, 0.6, 0.4)	(0.6, 0.4, 0.4)	(0.7, 0.3, 0.3)	(0.8, 0.2, 0.2)	(0.6, 0.4, 0.4)	(0.7, 0.3, 0.3)	(0.3, 0.7, 0.3)	(0.4, 0.6, 0.4)	(0.8, 0.2, 0.2)	(0.9, 0.1, 0.1)	(0.9, 0.1, 0.1)	(0.4, 0.6, 0.4)	(0.8, 0.2, 0.2)	(0.6, 0.4, 0.4)	(0.7, 0.3, 0.3)	(0.3, 0.7, 0.3)	(0.4, 0.6, 0.4)	(0.8, 0.2, 0.2)	(0.9, 0.1, 0.1)	(0.9, 0.1, 0.1)
WEA1	(0.6, 0.4, 0.4)	(0.7, 0.3, 0.3)	(0.4, 0.6, 0.4)	(0.8, 0.2, 0.2)	(0.6, 0.4, 0.4)	(0.7, 0.3, 0.3)	(0.3, 0.7, 0.3)	(0.4, 0.6, 0.4)	(0.8, 0.2, 0.2)	(0.9, 0.1, 0.1)	(0.3, 0.7, 0.3)	(0.8, 0.2, 0.2)	(0.8, 0.2, 0.2)	(0.6, 0.4, 0.4)	(0.7, 0.3, 0.3)	(0.3, 0.7, 0.3)	(0.4, 0.6, 0.4)	(0.8, 0.2, 0.2)	(0.9, 0.1, 0.1)	(0.1, 0.9, 0.1)
WEA2	(0.8, 0.2, 0.2)	(0.6, 0.4, 0.4)	(0.7, 0.3, 0.3)	(0.3, 0.7, 0.3)	(0.4, 0.6, 0.4)	(0.8, 0.2, 0.2)	(0.9, 0.1, 0.1)	(0.9, 0.1, 0.1)	(0.1, 0.9, 0.1)	(0.8, 0.2, 0.2)	(0.1, 0.9, 0.1)	(0.8, 0.2, 0.2)	(0.9, 0.1, 0.1)	(0.1, 0.9, 0.1)	(0.8, 0.2, 0.2)	(0.9, 0.1, 0.1)	(0.4, 0.6, 0.4)	(0.3, 0.7, 0.3)	(0.3, 0.7, 0.3)	(0.1, 0.9, 0.1)
WEA3	(0.8, 0.2, 0.2)	(0.6, 0.4, 0.4)	(0.7, 0.3, 0.3)	(0.3, 0.7, 0.3)	(0.4, 0.6, 0.4)	(0.8, 0.2, 0.2)	(0.9, 0.1, 0.1)	(0.8, 0.2, 0.2)	(0.9, 0.1, 0.1)	(0.8, 0.2, 0.2)	(0.6, 0.4, 0.4)	(0.7, 0.3, 0.3)	(0.3, 0.7, 0.3)	(0.4, 0.6, 0.4)	(0.8, 0.2, 0.2)	(0.9, 0.1, 0.1)	(0.9, 0.1, 0.1)	(0.4, 0.6, 0.4)	(0.3, 0.7, 0.3)	(0.8, 0.2, 0.2)
WEA4	(0.9, 0.1, 0.1)	(0.8, 0.2, 0.2)	(0.8, 0.2, 0.2)	(0.6, 0.4, 0.4)	(0.7, 0.3, 0.3)	(0.3, 0.7, 0.3)	(0.4, 0.6, 0.4)	(0.8, 0.2, 0.2)	(0.9, 0.1, 0.1)	(0.4, 0.6, 0.4)	(0.8, 0.2, 0.2)	(0.6, 0.4, 0.4)	(0.7, 0.3, 0.3)	(0.8, 0.2, 0.2)	(0.6, 0.4, 0.4)	(0.7, 0.3, 0.3)	(0.3, 0.7, 0.3)	(0.4, 0.6, 0.4)	(0.8, 0.2, 0.2)	(0.9, 0.1, 0.1)
WEA5	(0.8, 0.2, 0.2)	(0.6, 0.4, 0.4)	(0.7, 0.3, 0.3)	(0.3, 0.7, 0.3)	(0.4, 0.6, 0.4)	(0.8, 0.2, 0.2)	(0.9, 0.1, 0.1)	(0.9, 0.1, 0.1)	(0.4, 0.6, 0.4)	(0.3, 0.7, 0.3)	(0.9, 0.1, 0.1)	(0.2, 0.8, 0.2)	(0.4, 0.6, 0.4)	(0.8, 0.2, 0.2)	(0.6, 0.4, 0.4)	(0.7, 0.3, 0.3)	(0.3, 0.7, 0.3)	(0.4, 0.6, 0.4)	(0.8, 0.2, 0.2)	(0.9, 0.1, 0.1)
WEA6	(0.8, 0.2, 0.2)	(0.6, 0.4, 0.4)	(0.7, 0.3, 0.3)	(0.3, 0.7, 0.3)	(0.4, 0.6, 0.4)	(0.8, 0.2, 0.2)	(0.9, 0.1, 0.1)	(0.7, 0.3, 0.3)	(0.8, 0.2, 0.2)	(0.6, 0.4, 0.4)	(0.7, 0.3, 0.3)	(0.3, 0.7, 0.3)	(0.4, 0.6, 0.4)	(0.8, 0.2, 0.2)	(0.6, 0.4, 0.4)	(0.7, 0.3, 0.3)	(0.3, 0.7, 0.3)	(0.4, 0.6, 0.4)	(0.8, 0.2, 0.2)	(0.9, 0.1, 0.1)
THR1	(0.9, 0.1, 0.1)	(0.4, 0.6, 0.4)	(0.8, 0.2, 0.2)	(0.6, 0.4, 0.4)	(0.7, 0.3, 0.3)	(0.3, 0.7, 0.3)	(0.4, 0.6, 0.4)	(0.8, 0.2, 0.2)	(0.9, 0.1, 0.1)	(0.4, 0.6, 0.4)	(0.8, 0.2, 0.2)	(0.9, 0.1, 0.1)	(0.8, 0.2, 0.2)	(0.6, 0.4, 0.4)	(0.4, 0.6, 0.4)	(0.8, 0.2, 0.2)	(0.4, 0.6, 0.4)	(0.4, 0.6, 0.4)	(0.3, 0.7, 0.3)	(0.8, 0.2, 0.2)
THR2	(0.1, 0.9, 0.1)	(0.4, 0.6, 0.4)	(0.8, 0.2, 0.2)	(0.6, 0.4, 0.4)	(0.7, 0.3, 0.3)	(0.3, 0.7, 0.3)	(0.4, 0.6, 0.4)	(0.8, 0.2, 0.2)	(0.9, 0.1, 0.1)	(0.7, 0.3, 0.3)	(0.8, 0.2, 0.2)	(0.6, 0.4, 0.4)	(0.7, 0.3, 0.3)	(0.3, 0.7, 0.3)	(0.4, 0.6, 0.4)	(0.8, 0.2, 0.2)	(0.9, 0.1, 0.1)	(0.4, 0.6, 0.4)	(0.7, 0.3, 0.3)	(0.4, 0.6, 0.4)
THR3	(0.9, 0.1, 0.1)	(0.8, 0.2, 0.2)	(0.8, 0.2, 0.2)	(0.6, 0.4, 0.4)	(0.7, 0.3, 0.3)	(0.3, 0.7, 0.3)	(0.4, 0.6, 0.4)	(0.8, 0.2, 0.2)	(0.9, 0.1, 0.1)	(0.4, 0.6, 0.4)	(0.8, 0.2, 0.2)	(0.6, 0.4, 0.4)	(0.7, 0.3, 0.3)	(0.3, 0.7, 0.3)	(0.4, 0.6, 0.4)	(0.8, 0.2, 0.2)	(0.9, 0.1, 0.1)	(0.6, 0.4, 0.4)	(0.9, 0.1, 0.1)	(0.4, 0.6, 0.4)
THR4	(0.8, 0.2, 0.2)	(0.4, 0.6, 0.4)	(0.8, 0.2, 0.2)	(0.6, 0.4, 0.4)	(0.7, 0.3, 0.3)	(0.3, 0.7, 0.3)	(0.4, 0.6, 0.4)	(0.8, 0.2, 0.2)	(0.9, 0.1, 0.1)	(0.8, 0.2, 0.2)	(0.6, 0.4, 0.4)	(0.8, 0.2, 0.2)	(0.6, 0.4, 0.4)	(0.7, 0.3, 0.3)	(0.3, 0.7, 0.3)	(0.4, 0.6, 0.4)	(0.8, 0.2, 0.2)	(0.9, 0.1, 0.1)	(0.7, 0.3, 0.3)	(0.8, 0.2, 0.2)
THR5	(0.8, 0.2, 0.2)	(0.4, 0.6, 0.4)	(0.7, 0.3, 0.3)	(0.9, 0.1, 0.1)	(0.4, 0.6, 0.4)	(0.7, 0.3, 0.3)	(0.3, 0.7, 0.3)	(0.6, 0.4, 0.4)	(0.8, 0.2, 0.2)	(0.3, 0.7, 0.3)	(0.7, 0.3, 0.3)	(0.8, 0.2, 0.2)	(0.6, 0.4, 0.4)	(0.7, 0.3, 0.3)	(0.3, 0.7, 0.3)	(0.4, 0.6, 0.4)	(0.8, 0.2, 0.2)	(0.9, 0.1, 0.1)	(0.9, 0.1, 0.1)	(0.4, 0.6, 0.4)
THR6	(0.8, 0.2, 0.2)	(0.6, 0.4, 0.4)	(0.7, 0.3, 0.3)	(0.3, 0.7, 0.3)	(0.4, 0.6, 0.4)	(0.8, 0.2, 0.2)	(0.9, 0.1, 0.1)	(0.6, 0.4, 0.4)	(0.8, 0.2, 0.2)	(0.6, 0.4, 0.4)	(0.9, 0.1, 0.1)	(0.4, 0.6, 0.4)	(0.9, 0.1, 0.1)	(0.8, 0.2, 0.2)	(0.4, 0.6, 0.4)	(0.7, 0.3, 0.3)	(0.9, 0.1, 0.1)	(0.4, 0.6, 0.4)	(0.7, 0.3, 0.3)	(0.9, 0.1, 0.1)
THR7	(0.8, 0.2, 0.2)	(0.6, 0.4, 0.4)	(0.7, 0.3, 0.3)	(0.3, 0.7, 0.3)	(0.4, 0.6, 0.4)	(0.8, 0.2, 0.2)	(0.9, 0.1, 0.1)	(0.9, 0.1, 0.1)	(0.4, 0.6, 0.4)	(0.3, 0.7, 0.3)	(0.4, 0.6, 0.4)	(0.7, 0.3, 0.3)	(0.9, 0.1, 0.1)	(0.8, 0.2, 0.2)	(0.6, 0.4, 0.4)	(0.7, 0.3, 0.3)	(0.3, 0.7, 0.3)	(0.4, 0.6, 0.4)	(0.8, 0.2, 0.2)	(0.9, 0.1, 0.1)
THR8	(0.4, 0.6, 0.4)	(0.8, 0.2, 0.2)	(0.6, 0.4, 0.4)	(0.7, 0.3, 0.3)	(0.3, 0.7, 0.3)	(0.4, 0.6, 0.4)	(0.8, 0.2, 0.2)	(0.9, 0.1, 0.1)	(0.4, 0.6, 0.4)	(0.8, 0.2, 0.2)	(0.9, 0.1, 0.1)	(0.8, 0.2, 0.2)	(0.6, 0.4, 0.4)	(0.7, 0.3, 0.3)	(0.3, 0.7, 0.3)	(0.4, 0.6, 0.4)	(0.8, 0.2, 0.2)	(0.9, 0.1, 0.1)	(0.7, 0.3, 0.3)	(0.1, 0.9, 0.1)
THR9	(0.8, 0.2, 0.2)	(0.6, 0.4, 0.4)	(0.7, 0.3, 0.3)	(0.3, 0.7, 0.3)	(0.4, 0.6, 0.4)	(0.8, 0.2, 0.2)	(0.9, 0.1, 0.1)	(0.8, 0.2, 0.2)	(0.1, 0.9, 0.1)	(0.8, 0.2, 0.2)	(0.6, 0.4, 0.4)	(0.7, 0.3, 0.3)	(0.3, 0.7, 0.3)	(0.4, 0.6, 0.4)	(0.8, 0.2, 0.2)	(0.9, 0.1, 0.1)	(0.2, 0.8, 0.2)	(0.6, 0.4, 0.4)	(0.3, 0.7, 0.3)	(0.8, 0.2, 0.2)
THR10	(0.8, 0.2, 0.2)	(0.6, 0.4, 0.4)	(0.7, 0.3, 0.3)	(0.3, 0.7, 0.3)	(0.4, 0.6, 0.4)	(0.8, 0.2, 0.2)	(0.9, 0.1, 0.1)	(0.4, 0.6, 0.4)	(0.3, 0.7, 0.3)	(0.8, 0.2, 0.2)	(0.6, 0.4, 0.4)	(0.7, 0.3, 0.3)	(0.3, 0.7, 0.3)	(0.4, 0.6, 0.4)	(0.8, 0.2, 0.2)	(0.9, 0.1, 0.1)	(0.4, 0.6, 0.4)	(0.3, 0.7, 0.3)	(0.8, 0.2, 0.2)	(0.9, 0.1, 0.1)

Table A3. The decision matrix between overall criteria and alternatives by the GRA method for third experts.

	GME1	GME2	GME3	GME4	GME5	GME6	GME7	GME8	GME9	GME10	GME11	GME12	GME13	GME14	GME15	GME16	GME17	GME18	GME19	GME20
STRA1	(0.1, 0.9, 0.1)	(0.8, 0.2, 0.2)	(0.6, 0.4, 0.4)	(0.7, 0.3, 0.3)	(0.2, 0.8, 0.2)	(0.9, 0.1, 0.1)	(0.8, 0.2, 0.2)	(0.9, 0.1, 0.1)	(0.9, 0.1, 0.1)	(0.1, 0.9, 0.1)	(0.8, 0.2, 0.2)	(0.6, 0.4, 0.4)	(0.7, 0.3, 0.3)	(0.1, 0.9, 0.1)	(0.8, 0.2, 0.2)	(0.6, 0.4, 0.4)	(0.7, 0.3, 0.3)	(0.2, 0.8, 0.2)	(0.9, 0.1, 0.1)	(0.8, 0.2, 0.2)
STRA2	(0.9, 0.1, 0.1)	(0.8, 0.2, 0.2)	(0.6, 0.4, 0.4)	(0.8, 0.2, 0.2)	(0.1, 0.9, 0.1)	(0.8, 0.2, 0.2)	(0.6, 0.4, 0.4)	(0.7, 0.3, 0.3)	(0.2, 0.8, 0.2)	(0.9, 0.1, 0.1)	(0.8, 0.2, 0.2)	(0.9, 0.1, 0.1)	(0.8, 0.2, 0.2)	(0.1, 0.9, 0.1)	(0.8, 0.2, 0.2)	(0.6, 0.4, 0.4)	(0.7, 0.3, 0.3)	(0.2, 0.8, 0.2)	(0.9, 0.1, 0.1)	(0.8, 0.2, 0.2)
STRA3	(0.8, 0.2, 0.2)	(0.1, 0.9, 0.1)	(0.8, 0.2, 0.2)	(0.6, 0.4, 0.4)	(0.7, 0.3, 0.3)	(0.2, 0.8, 0.2)	(0.9, 0.1, 0.1)	(0.8, 0.2, 0.2)	(0.8, 0.2, 0.2)	(0.1, 0.9, 0.1)	(0.1, 0.9, 0.1)	(0.8, 0.2, 0.2)	(0.6, 0.4, 0.4)	(0.7, 0.3, 0.3)	(0.2, 0.8, 0.2)	(0.9, 0.1, 0.1)	(0.8, 0.2, 0.2)	(0.4, 0.6, 0.4)	(0.8, 0.2, 0.2)	(0.9, 0.1, 0.1)
STRA4	(0.1, 0.9, 0.1)	(0.8, 0.2, 0.2)	(0.6, 0.4, 0.4)	(0.7, 0.3, 0.3)	(0.2, 0.8, 0.2)	(0.9, 0.1, 0.1)	(0.8, 0.2, 0.2)	(0.4, 0.6, 0.4)	(0.6, 0.4, 0.4)	(0.7, 0.3, 0.3)	(0.1, 0.9, 0.1)	(0.8, 0.2, 0.2)	(0.6, 0.4, 0.4)	(0.7, 0.3, 0.3)	(0.2, 0.8, 0.2)	(0.9, 0.1, 0.1)	(0.8, 0.2, 0.2)	(0.4, 0.6, 0.4)	(0.8, 0.2, 0.2)	(0.9, 0.1, 0.1)
STRA5	(0.4, 0.6, 0.4)	(0.1, 0.9, 0.1)	(0.8, 0.2, 0.2)	(0.6, 0.4, 0.4)	(0.7, 0.3, 0.3)	(0.2, 0.8, 0.2)	(0.9, 0.1, 0.1)	(0.8, 0.2, 0.2)	(0.1, 0.9, 0.1)	(0.1, 0.9, 0.1)	(0.8, 0.2, 0.2)	(0.6, 0.4, 0.4)	(0.7, 0.3, 0.3)	(0.2, 0.8, 0.2)	(0.9, 0.1, 0.1)	(0.8, 0.2, 0.2)	(0.3, 0.7, 0.3)	(0.4, 0.6, 0.4)	(0.8, 0.2, 0.2)	(0.9, 0.1, 0.1)
STRA6	(0.1, 0.9, 0.1)	(0.8, 0.2, 0.2)	(0.6, 0.4, 0.4)	(0.7, 0.3, 0.3)	(0.2, 0.8, 0.2)	(0.9, 0.1, 0.1)	(0.8, 0.2, 0.2)	(0.8, 0.2, 0.2)	(0.6, 0.4, 0.4)	(0.1, 0.9, 0.1)	(0.8, 0.2, 0.2)	(0.6, 0.4, 0.4)	(0.7, 0.3, 0.3)	(0.2, 0.8, 0.2)	(0.9, 0.1, 0.1)	(0.8, 0.2, 0.2)	(0.3, 0.7, 0.3)	(0.4, 0.6, 0.4)	(0.8, 0.2, 0.2)	(0.9, 0.1, 0.1)
STRA7	(0.4, 0.6, 0.4)	(0.8, 0.2, 0.2)	(0.1, 0.9, 0.1)	(0.2, 0.8, 0.2)	(0.6, 0.4, 0.4)	(0.1, 0.9, 0.1)	(0.2, 0.8, 0.2)	(0.8, 0.2, 0.2)	(0.6, 0.4, 0.4)	(0.7, 0.3, 0.3)	(0.3, 0.7, 0.3)	(0.4, 0.6, 0.4)	(0.8, 0.2, 0.2)	(0.9, 0.1, 0.1)	(0.1, 0.9, 0.1)	(0.2, 0.8, 0.2)	(0.6, 0.4, 0.4)	(0.1, 0.9, 0.1)	(0.2, 0.8, 0.2)	(0.6, 0.4, 0.4)
STRA8	(0.1, 0.9, 0.1)	(0.8, 0.2, 0.2)	(0.6, 0.4, 0.4)	(0.7, 0.3, 0.3)	(0.2, 0.8, 0.2)	(0.9, 0.1, 0.1)	(0.8, 0.2, 0.2)	(0.7, 0.3, 0.3)	(0.3, 0.7, 0.3)	(0.4, 0.6, 0.4)	(0.8, 0.2, 0.2)	(0.1, 0.9, 0.1)	(0.8, 0.2, 0.2)	(0.6, 0.4, 0.4)	(0.7, 0.3, 0.3)	(0.2, 0.8, 0.2)	(0.9, 0.1, 0.1)	(0.8, 0.2, 0.2)	(0.3, 0.7, 0.3)	(0.4, 0.6, 0.4)
STRA9	(0.1, 0.9, 0.1)	(0.8, 0.2, 0.2)	(0.6, 0.4, 0.4)	(0.7, 0.3, 0.3)	(0.2, 0.8, 0.2)	(0.9, 0.1, 0.1)	(0.8, 0.2, 0.2)	(0.8, 0.2, 0.2)	(0.9, 0.1, 0.1)	(0.6, 0.4, 0.4)	(0.7, 0.3, 0.3)	(0.3, 0.7, 0.3)	(0.1, 0.9, 0.1)	(0.8, 0.2, 0.2)	(0.6, 0.4, 0.4)	(0.7, 0.3, 0.3)	(0.2, 0.8, 0.2)	(0.9, 0.1, 0.1)	(0.8, 0.2, 0.2)	(0.9, 0.1, 0.1)
STRA10	(0.1, 0.9, 0.1)	(0.8, 0.2, 0.2)	(0.6, 0.4, 0.4)	(0.7, 0.3, 0.3)	(0.2, 0.8, 0.2)	(0.9, 0.1, 0.1)	(0.8, 0.2, 0.2)	(0.9, 0.1, 0.1)	(0.8, 0.2, 0.2)	(0.4, 0.6, 0.4)	(0.9, 0.1, 0.1)	(0.1, 0.9, 0.1)	(0.8, 0.2, 0.2)	(0.6, 0.4, 0.4)	(0.7, 0.3, 0.3)	(0.2, 0.8, 0.2)	(0.9, 0.1, 0.1)	(0.8, 0.2, 0.2)	(0.8, 0.2, 0.2)	(0.9, 0.1, 0.1)
OPP1	(0.8, 0.2, 0.2)	(0.1, 0.9, 0.1)	(0.8, 0.2, 0.2)	(0.6, 0.4, 0.4)	(0.7, 0.3, 0.3)	(0.2, 0.8, 0.2)	(0.1, 0.9, 0.1)	(0.8, 0.2, 0.2)	(0.6, 0.4, 0.4)	(0.7, 0.3, 0.3)	(0.2, 0.8, 0.2)	(0.9, 0.1, 0.1)	(0.8, 0.2, 0.2)	(0.2, 0.8, 0.2)	(0.9, 0.1, 0.1)	(0.8, 0.2, 0.2)	(0.3, 0.7, 0.3)	(0.4, 0.6, 0.4)	(0.8, 0.2, 0.2)	(0.9, 0.1, 0.1)
OPP2	(0.1, 0.9, 0.1)	(0.8, 0.2, 0.2)	(0.6, 0.4, 0.4)	(0.7, 0.3, 0.3)	(0.2, 0.8, 0.2)	(0.9, 0.1, 0.1)	(0.8, 0.2, 0.2)	(0.8, 0.2, 0.2)	(0.6, 0.4, 0.4)	(0.7, 0.3, 0.3)	(0.2, 0.8, 0.2)	(0.9, 0.1, 0.1)	(0.8, 0.2, 0.2)	(0.6, 0.4, 0.4)	(0.1, 0.9, 0.1)	(0.8, 0.2, 0.2)	(0.6, 0.4, 0.4)	(0.7, 0.3, 0.3)	(0.2, 0.8, 0.2)	(0.9, 0.1, 0.1)
OPP3	(0.1, 0.9, 0.1)	(0.8, 0.2, 0.2)	(0.6, 0.4, 0.4)	(0.7, 0.3, 0.3)	(0.2, 0.8, 0.2)	(0.9, 0.1, 0.1)	(0.8, 0.2, 0.2)	(0.8, 0.2, 0.2)	(0.9, 0.1, 0.1)	(0.4, 0.6, 0.4)	(0.1, 0.9, 0.1)	(0.8, 0.2, 0.2)	(0.6, 0.4, 0.4)	(0.7, 0.3, 0.3)	(0.2, 0.8, 0.2)	(0.9, 0.1, 0.1)	(0.8, 0.2, 0.2)	(0.9, 0.1, 0.1)	(0.7, 0.3, 0.3)	(0.4, 0.6, 0.4)
OPP4	(0.9, 0.1, 0.1)	(0.8, 0.2, 0.2)	(0.1, 0.9, 0.1)	(0.8, 0.2, 0.2)	(0.6, 0.4, 0.4)	(0.7, 0.3, 0.3)	(0.2, 0.8, 0.2)	(0.9, 0.1, 0.1)	(0.8, 0.2, 0.2)	(0.6, 0.4, 0.4)	(0.7, 0.3, 0.3)	(0.2, 0.8, 0.2)	(0.9, 0.1, 0.1)	(0.8, 0.2, 0.2)	(0.1, 0.9, 0.1)	(0.8, 0.2, 0.2)	(0.6, 0.4, 0.4)	(0.7, 0.3, 0.3)	(0.2, 0.8, 0.2)	(0.9, 0.1, 0.1)
OPP5	(0.8, 0.2, 0.2)	(0.9, 0.1, 0.1)	(0.1, 0.9, 0.1)	(0.8, 0.2, 0.2)	(0.6, 0.4, 0.4)	(0.7, 0.3, 0.3)	(0.2, 0.8, 0.2)	(0.9, 0.1, 0.1)	(0.8, 0.2, 0.2)	(0.6, 0.4, 0.4)	(0.7, 0.3, 0.3)	(0.3, 0.7, 0.3)	(0.1, 0.9, 0.1)	(0.8, 0.2, 0.2)	(0.6, 0.4, 0.4)	(0.7, 0.3, 0.3)	(0.2, 0.8, 0.2)	(0.9, 0.1, 0.1)	(0.8, 0.2, 0.2)	(0.8, 0.2, 0.2)
OPP6	(0.9, 0.1, 0.1)	(0.1, 0.9, 0.1)	(0.8, 0.2, 0.2)	(0.6, 0.4, 0.4)	(0.7, 0.3, 0.3)	(0.2, 0.8, 0.2)	(0.9, 0.1, 0.1)	(0.8, 0.2, 0.2)	(0.3, 0.7, 0.3)	(0.4, 0.6, 0.4)	(0.8, 0.2, 0.2)	(0.6, 0.4, 0.4)	(0.1, 0.9, 0.1)	(0.8, 0.2, 0.2)	(0.6, 0.4, 0.4)	(0.7, 0.3, 0.3)	(0.2, 0.8, 0.2)	(0.9, 0.1, 0.1)	(0.8, 0.2, 0.2)	(0.6, 0.4, 0.4)
OPP7	(0.9, 0.1, 0.1)	(0.1, 0.9, 0.1)	(0.8, 0.2, 0.2)	(0.6, 0.4, 0.4)	(0.7, 0.3, 0.3)	(0.2, 0.8, 0.2)	(0.9, 0.1, 0.1)	(0.8, 0.2, 0.2)	(0.3, 0.7, 0.3)	(0.4, 0.6, 0.4)	(0.8, 0.2, 0.2)	(0.1, 0.9, 0.1)	(0.8, 0.2, 0.2)	(0.6, 0.4, 0.4)	(0.7, 0.3, 0.3)	(0.2, 0.8, 0.2)	(0.9, 0.1, 0.1)	(0.8, 0.2, 0.2)	(0.8, 0.2, 0.2)	(0.9, 0.1, 0.1)
OPP8	(0.1, 0.9, 0.1)	(0.8, 0.2, 0.2)	(0.6, 0.4, 0.4)	(0.7, 0.3, 0.3)	(0.2, 0.8, 0.2)	(0.9, 0.1, 0.1)	(0.8, 0.2, 0.2)	(0.4, 0.6, 0.4)	(0.6, 0.4, 0.4)	(0.7, 0.3, 0.3)	(0.3, 0.7, 0.3)	(0.1, 0.9, 0.1)	(0.8, 0.2, 0.2)	(0.6, 0.4, 0.4)	(0.7, 0.3, 0.3)	(0.2, 0.8, 0.2)	(0.9, 0.1, 0.1)	(0.8, 0.2, 0.2)	(0.7, 0.3, 0.3)	(0.1, 0.9, 0.1)
OPP9	(0.9, 0.1, 0.1)	(0.1, 0.9, 0.1)	(0.8, 0.2, 0.2)	(0.6, 0.4, 0.4)	(0.7, 0.3, 0.3)	(0.2, 0.8, 0.2)	(0.9, 0.1, 0.1)	(0.8, 0.2, 0.2)	(0.4, 0.6, 0.4)	(0.6, 0.4, 0.4)	(0.7, 0.3, 0.3)	(0.1, 0.9, 0.1)	(0.8, 0.2, 0.2)	(0.6, 0.4, 0.4)	(0.7, 0.3, 0.3)	(0.2, 0.8, 0.2)	(0.9, 0.1, 0.1)	(0.8, 0.2, 0.2)	(0.7, 0.3, 0.3)	(0.1, 0.9, 0.1)
OPP10	(0.8, 0.2, 0.2)	(0.6, 0.4, 0.4)	(0.1, 0.9, 0.1)	(0.8, 0.2, 0.2)	(0.6, 0.4, 0.4)	(0.7, 0.3, 0.3)	(0.2, 0.8, 0.2)	(0.9, 0.1, 0.1)	(0.8, 0.2, 0.2)	(0.8, 0.2, 0.2)	(0.6, 0.4, 0.4)	(0.1, 0.9, 0.1)	(0.8, 0.2, 0.2)	(0.6, 0.4, 0.4)	(0.7, 0.3, 0.3)	(0.2, 0.8, 0.2)	(0.9, 0.1, 0.1)	(0.8, 0.2, 0.2)	(0.9, 0.1, 0.1)	(0.6, 0.4, 0.4)
OPP11	(0.4, 0.6, 0.4)	(0.6, 0.4, 0.4)	(0.1, 0.9, 0.1)	(0.8, 0.2, 0.2)	(0.1, 0.9, 0.1)	(0.8, 0.2, 0.2)	(0.6, 0.4, 0.4)	(0.7, 0.3, 0.3)	(0.2, 0.8, 0.2)	(0.9, 0.1, 0.1)	(0.8, 0.2, 0.2)	(0.4, 0.6, 0.4)	(0.1, 0.9, 0.1)	(0.8, 0.2, 0.2)	(0.6, 0.4, 0.4)	(0.7, 0.3, 0.3)	(0.2, 0.8, 0.2)	(0.9, 0.1, 0.1)	(0.8, 0.2, 0.2)	(0.9, 0.1, 0.1)
WEA1	(0.6, 0.4, 0.4)	(0.7, 0.3, 0.3)	(0.4, 0.6, 0.4)	(0.8, 0.2, 0.2)	(0.6, 0.4, 0.4)	(0.7, 0.3, 0.3)	(0.3, 0.7, 0.3)	(0.4, 0.6, 0.4)	(0.8, 0.2, 0.2)	(0.9, 0.1, 0.1)	(0.3, 0.7, 0.3)	(0.8, 0.2, 0.2)	(0.1, 0.9, 0.1)	(0.8, 0.2, 0.2)	(0.6, 0.4, 0.4)	(0.7, 0.3, 0.3)	(0.2, 0.8, 0.2)	(0.9, 0.1, 0.1)	(0.8, 0.2, 0.2)	(0.1, 0.9, 0.1)
WEA2	(0.8, 0.2, 0.2)	(0.6, 0.4, 0.4)	(0.7, 0.3, 0.3)	(0.3, 0.7, 0.3)	(0.4, 0.6, 0.4)	(0.8, 0.2, 0.2)	(0.9, 0.1, 0.1)	(0.9, 0.1, 0.1)	(0.1, 0.9, 0.1)	(0.8, 0.2, 0.2)	(0.1, 0.9, 0.1)	(0.1, 0.9, 0.1)	(0.8, 0.2, 0.2)	(0.6, 0.4, 0.4)	(0.7, 0.3, 0.3)	(0.2, 0.8, 0.2)	(0.9, 0.1, 0.1)	(0.8, 0.2, 0.2)	(0.3, 0.7, 0.3)	(0.1, 0.9, 0.1)
WEA3	(0.8, 0.2, 0.2)	(0.1, 0.9, 0.1)	(0.8, 0.2, 0.2)	(0.6, 0.4, 0.4)	(0.7, 0.3, 0.3)	(0.2, 0.8, 0.2)	(0.9, 0.1, 0.1)	(0.8, 0.2, 0.2)	(0.9, 0.1, 0.1)	(0.8, 0.2, 0.2)	(0.6, 0.4, 0.4)	(0.1, 0.9, 0.1)	(0.8, 0.2, 0.2)	(0.6, 0.4, 0.4)	(0.7, 0.3, 0.3)	(0.2, 0.8, 0.2)	(0.9, 0.1, 0.1)	(0.8, 0.2, 0.2)	(0.3, 0.7, 0.3)	(0.8, 0.2, 0.2)
WEA4	(0.9, 0.1, 0.1)	(0.8, 0.2, 0.2)	(0.8, 0.2, 0.2)	(0.6, 0.4, 0.4)	(0.7, 0.3, 0.3)	(0.3, 0.7, 0.3)	(0.4, 0.6, 0.4)	(0.8, 0.2, 0.2)	(0.9, 0.1, 0.1)	(0.4, 0.6, 0.4)	(0.8, 0.2, 0.2)	(0.6, 0.4, 0.4)	(0.7, 0.3, 0.3)	(0.8, 0.2, 0.2)	(0.6, 0.4, 0.4)	(0.7, 0.3, 0.3)	(0.3, 0.7, 0.3)	(0.4, 0.6, 0.4)	(0.8, 0.2, 0.2)	(0.9, 0.1, 0.1)
WEA5	(0.1, 0.9, 0.1)	(0.8, 0.2, 0.2)	(0.6, 0.4, 0.4)	(0.7, 0.3, 0.3)	(0.2, 0.8, 0.2)	(0.9, 0.1, 0.1)	(0.8, 0.2, 0.2)	(0.9, 0.1, 0.1)	(0.4, 0.6, 0.4)	(0.3, 0.7, 0.3)	(0.9, 0.1, 0.1)	(0.1, 0.9, 0.1)	(0.8, 0.2, 0.2)	(0.6, 0.4, 0.4)	(0.7, 0.3, 0.3)	(0.2, 0.8, 0.2)	(0.9, 0.1, 0.1)	(0.8, 0.2, 0.2)	(0.8, 0.2, 0.2)	(0.9, 0.1, 0.1)
WEA6	(0.1, 0.9, 0.1)	(0.8, 0.2, 0.2)	(0.6, 0.4, 0.4)	(0.7, 0.3, 0.3)	(0.2, 0.8, 0.2)	(0.9, 0.1, 0.1)	(0.8, 0.2, 0.2)	(0.7, 0.3, 0.3)	(0.8, 0.2, 0.2)	(0.6, 0.4, 0.4)	(0.7, 0.3, 0.3)	(0.1, 0.9, 0.1)	(0.8, 0.2, 0.2)	(0.6, 0.4, 0.4)	(0.7, 0.3, 0.3)	(0.2, 0.8, 0.2)	(0.9, 0.1, 0.1)	(0.8, 0.2, 0.2)	(0.8, 0.2, 0.2)	(0.9, 0.1, 0.1)
THR1	(0.1, 0.9, 0.1)	(0.8, 0.2, 0.2)	(0.6, 0.4, 0.4)	(0.7, 0.3, 0.3)	(0.2, 0.8, 0.2)	(0.9, 0.1, 0.1)	(0.8, 0.2, 0.2)	(0.8, 0.2, 0.2)	(0.9, 0.1, 0.1)	(0.4, 0.6, 0.4)	(0.8, 0.2, 0.2)	(0.9, 0.1, 0.1)	(0.8, 0.2, 0.2)	(0.6, 0.4, 0.4)	(0.1, 0.9, 0.1)	(0.8, 0.2, 0.2)	(0.6, 0.4, 0.4)	(0.7, 0.3, 0.3)	(0.2, 0.8, 0.2)	(0.9, 0.1, 0.1)
THR2	(0.1, 0.9, 0.1)	(0.8, 0.2, 0.2)	(0.6, 0.4, 0.4)	(0.7, 0.3, 0.3)	(0.2, 0.8, 0.2)	(0.9, 0.1, 0.1)	(0.8, 0.2, 0.2)	(0.8, 0.2, 0.2)	(0.9, 0.1, 0.1)	(0.7, 0.3, 0.3)	(0.8, 0.2, 0.2)	(0.1, 0.9, 0.1)	(0.8, 0.2, 0.2)	(0.6, 0.4, 0.4)	(0.7, 0.3, 0.3)	(0.2, 0.8, 0.2)	(0.9, 0.1, 0.1)	(0.8, 0.2, 0.2)	(0.7, 0.3, 0.3)	(0.4, 0.6, 0.4)
THR3	(0.9, 0.1, 0.1)	(0.8, 0.2, 0.2)	(0.8, 0.2, 0.2)	(0.1, 0.9, 0.1)	(0.8, 0.2, 0.2)	(0.6, 0.4, 0.4)	(0.7, 0.3, 0.3)	(0.2, 0.8, 0.2)	(0.9, 0.1, 0.1)	(0.8, 0.2, 0.2)	(0.8, 0.2, 0.2)	(0.6, 0.4, 0.4)	(0.7, 0.3, 0.3)	(0.1, 0.9, 0.1)	(0.8, 0.2, 0.2)	(0.6, 0.4, 0.4)	(0.7, 0.3, 0.3)	(0.2, 0.8, 0.2)	(0.9, 0.1, 0.1)	(0.8, 0.2, 0.2)
THR4	(0.8, 0.2, 0.2)	(0.4, 0.6, 0.4)	(0.8, 0.2, 0.2)	(0.6, 0.4, 0.4)	(0.7, 0.3, 0.3)	(0.3, 0.7, 0.3)	(0.4, 0.6, 0.4)	(0.8, 0.2, 0.2)	(0.9, 0.1, 0.1)	(0.8, 0.2, 0.2)	(0.6, 0.4, 0.4)	(0.8, 0.2, 0.2)	(0.6, 0.4, 0.4)	(0.7, 0.3, 0.3)	(0.3, 0.7, 0.3)	(0.4, 0.6, 0.4)	(0.8, 0.2, 0.2)	(0.9, 0.1, 0.1)	(0.7, 0.3, 0.3)	(0.8, 0.2, 0.2)
THR5	(0.8, 0.2, 0.2)	(0.4, 0.6, 0.4)	(0.7, 0.3, 0.3)	(0.9, 0.1, 0.1)	(0.1, 0.9, 0.1)	(0.8, 0.2, 0.2)	(0.6, 0.4, 0.4)	(0.7, 0.3, 0.3)	(0.2, 0.8, 0.2)	(0.9, 0.1, 0.1)	(0.8, 0.2, 0.2)	(0.8, 0.2, 0.2)	(0.1, 0.9, 0.1)	(0.8, 0.2, 0.2)	(0.6, 0.4, 0.4)	(0.7, 0.3, 0.3)	(0.2, 0.8, 0.2)	(0.9, 0.1, 0.1)	(0.8, 0.2, 0.2)	(0.4, 0.6, 0.4)
THR6	(0.8, 0.2, 0.2)	(0.6, 0.4, 0.4)	(0.7, 0.3, 0.3)	(0.3, 0.7, 0.3)	(0.4, 0.6, 0.4)	(0.8, 0.2, 0.2)	(0.9, 0.1, 0.1)	(0.6, 0.4, 0.4)	(0.8, 0.2, 0.2)	(0.6, 0.4, 0.4)	(0.9, 0.1, 0.1)	(0.4, 0.6, 0.4)	(0.9, 0.1, 0.1)	(0.8, 0.2, 0.2)	(0.4, 0.6, 0.4)	(0.7, 0.3, 0.3)	(0.9, 0.1, 0.1)	(0.4, 0.6, 0.4)	(0.7, 0.3, 0.3)	(0.9, 0.1, 0.1)
THR7	(0.8, 0.2, 0.2)	(0.6, 0.4, 0.4)	(0.7, 0.3, 0.3)	(0.3, 0.7, 0.3)	(0.4, 0.6, 0.4)	(0.1, 0.9, 0.1)	(0.8, 0.2, 0.2)	(0.6, 0.4, 0.4)	(0.7, 0.3, 0.3)	(0.2, 0.8, 0.2)	(0.9, 0.1, 0.1)	(0.8, 0.2, 0.2)	(0.9, 0.1, 0.1)	(0.8, 0.2, 0.2)	(0.6, 0.4, 0.4)	(0.7, 0.3, 0.3)	(0.3, 0.7, 0.3)	(0.4, 0.6, 0.4)	(0.8, 0.2, 0.2)	(0.9, 0.1, 0.1)
THR8	(0.4, 0.6, 0.4)	(0.8, 0.2, 0.2)	(0.1, 0.9, 0.1)	(0.8, 0.2, 0.2)	(0.6, 0.4, 0.4)	(0.7, 0.3, 0.3)	(0.2, 0.8, 0.2)	(0.9, 0.1, 0.1)	(0.8, 0.2, 0.2)	(0.9, 0.1, 0.1)	(0.8, 0.2, 0.2)	(0.8, 0.2, 0.2)	(0.6, 0.4, 0.4)	(0.7, 0.3, 0.3)	(0.3, 0.7, 0.3)	(0.4, 0.6, 0.4)	(0.8, 0.2, 0.2)	(0.9, 0.1, 0.1)	(0.7, 0.3, 0.3)	(0.1, 0.9, 0.1)
THR9	(0.8, 0.2, 0.2)	(0.6, 0.4, 0.4)	(0.1, 0.9, 0.1)	(0.8, 0.2, 0.2)	(0.6, 0.4, 0.4)	(0.7, 0.3, 0.3)	(0.2, 0.8, 0.2)	(0.9, 0.1, 0.1)	(0.8, 0.2, 0.2)	(0.8, 0.2, 0.2)	(0.6, 0.4, 0.4)	(0.1, 0.9, 0.1)	(0.8, 0.2, 0.2)	(0.6, 0.4, 0.4)	(0.7, 0.3, 0.3)	(0.2, 0.8, 0.2)	(0.9, 0.1, 0.1)	(0.8, 0.2, 0.2)	(0.3, 0.7, 0.3)	(0.8, 0.2, 0.2)
THR10	(0.8, 0.2, 0.2)	(0.6, 0.4, 0.4)	(0.1, 0.9, 0.1)	(0.8, 0.2, 0.2)	(0.6, 0.4, 0.4)	(0.7, 0.3, 0.3)	(0.2, 0.8, 0.2)	(0.9, 0.1, 0.1)	(0.8, 0.2, 0.2)	(0.8, 0.2, 0.2)	(0.6, 0.4, 0.4)	(0.1, 0.9, 0.1)	(0.8, 0.2, 0.2)	(0.6, 0.4, 0.4)	(0.7, 0.3, 0.3)	(0.2, 0.8, 0.2)	(0.9, 0.1, 0.1)	(0.8, 0.2, 0.2)	(0.8, 0.2, 0.2)	(0.9, 0.1, 0.1)

Table A4. The decision matrix between overall criteria and alternatives by the GRA method for fourth experts.

	GME1	GME2	GME3	GME4	GME5	GME6	GME7	GME8	GME9	GME10	GME11	GME12	GME13	GME14	GME15	GME16	GME17	GME18	GME19	GME20
STRA1	(0.7, 0.3, 0.3)	(0.3, 0.7, 0.3)	(0.6, 0.4, 0.4)	(0.4, 0.6, 0.4)	(0.1, 0.9, 0.1)	(0.2, 0.8, 0.2)	(0.7, 0.3, 0.3)	(0.6, 0.4, 0.4)	(0.3, 0.7, 0.3)	(0.8, 0.2, 0.2)	(0.9, 0.1, 0.1)	(0.4, 0.6, 0.4)	(0.7, 0.3, 0.3)	(0.3, 0.7, 0.3)	(0.6, 0.4, 0.4)	(0.4, 0.6, 0.4)	(0.1, 0.9, 0.1)	(0.2, 0.8, 0.2)	(0.7, 0.3, 0.3)	(0.6, 0.4, 0.4)
STRA2	(0.8, 0.2, 0.2)	(0.7, 0.3, 0.3)	(0.3, 0.7, 0.3)	(0.6, 0.4, 0.4)	(0.4, 0.6, 0.4)	(0.1, 0.9, 0.1)	(0.2, 0.8, 0.2)	(0.7, 0.3, 0.3)	(0.6, 0.4, 0.4)	(0.4, 0.6, 0.4)	(0.9, 0.1, 0.1)	(0.2, 0.8, 0.2)	(0.7, 0.3, 0.3)	(0.3, 0.7, 0.3)	(0.6, 0.4, 0.4)	(0.4, 0.6, 0.4)	(0.1, 0.9, 0.1)	(0.2, 0.8, 0.2)	(0.7, 0.3, 0.3)	(0.6, 0.4, 0.4)
STRA3	(0.7, 0.3, 0.3)	(0.3, 0.7, 0.3)	(0.6, 0.4, 0.4)	(0.4, 0.6, 0.4)	(0.1, 0.9, 0.1)	(0.2, 0.8, 0.2)	(0.7, 0.3, 0.3)	(0.6, 0.4, 0.4)	(0.2, 0.8, 0.2)	(0.8, 0.2, 0.2)	(0.6, 0.4, 0.4)	(0.8, 0.2, 0.2)	(0.7, 0.3, 0.3)	(0.3, 0.7, 0.3)	(0.6, 0.4, 0.4)	(0.4, 0.6, 0.4)	(0.1, 0.9, 0.1)	(0.2, 0.8, 0.2)	(0.7, 0.3, 0.3)	(0.6, 0.4, 0.4)
STRA4	(0.9, 0.1, 0.1)	(0.7, 0.3, 0.3)	(0.3, 0.7, 0.3)	(0.6, 0.4, 0.4)	(0.4, 0.6, 0.4)	(0.1, 0.9, 0.1)	(0.2, 0.8, 0.2)	(0.7, 0.3, 0.3)	(0.6, 0.4, 0.4)	(0.4, 0.6, 0.4)	(0.4, 0.6, 0.4)	(0.3, 0.7, 0.3)	(0.7, 0.3, 0.3)	(0.3, 0.7, 0.3)	(0.6, 0.4, 0.4)	(0.4, 0.6, 0.4)	(0.1, 0.9, 0.1)	(0.2, 0.8, 0.2)	(0.7, 0.3, 0.3)	(0.6, 0.4, 0.4)
STRA5	(0.1, 0.9, 0.1)	(0.8, 0.2, 0.2)	(0.4, 0.6, 0.4)	(0.4, 0.6, 0.4)	(0.4, 0.6, 0.4)	(0.8, 0.2, 0.2)	(0.9, 0.1, 0.1)	(0.4, 0.6, 0.4)	(0.3, 0.7, 0.3)	(0.8, 0.2, 0.2)	(0.6, 0.4, 0.4)	(0.9, 0.1, 0.1)	(0.7, 0.3, 0.3)	(0.3, 0.7, 0.3)	(0.6, 0.4, 0.4)	(0.4, 0.6, 0.4)	(0.1, 0.9, 0.1)	(0.2, 0.8, 0.2)	(0.7, 0.3, 0.3)	(0.6, 0.4, 0.4)
STRA6	(0.8, 0.2, 0.2)	(0.7, 0.3, 0.3)	(0.3, 0.7, 0.3)	(0.6, 0.4, 0.4)	(0.4, 0.6, 0.4)	(0.1, 0.9, 0.1)	(0.2, 0.8, 0.2)	(0.7, 0.3, 0.3)	(0.6, 0.4, 0.4)	(0.4, 0.6, 0.4)	(0.1, 0.9, 0.1)	(0.9, 0.1, 0.1)	(0.7, 0.3, 0.3)	(0.3, 0.7, 0.3)	(0.6, 0.4, 0.4)	(0.4, 0.6, 0.4)	(0.1, 0.9, 0.1)	(0.2, 0.8, 0.2)	(0.7, 0.3, 0.3)	(0.6, 0.4, 0.4)
STRA7	(0.8, 0.2, 0.2)	(0.7, 0.3, 0.3)	(0.7, 0.3, 0.3)	(0.3, 0.7, 0.3)	(0.6, 0.4, 0.4)	(0.4, 0.6, 0.4)	(0.1, 0.9, 0.1)	(0.2, 0.8, 0.2)	(0.7, 0.3, 0.3)	(0.6, 0.4, 0.4)	(0.9, 0.1, 0.1)	(0.4, 0.6, 0.4)	(0.7, 0.3, 0.3)	(0.3, 0.7, 0.3)	(0.6, 0.4, 0.4)	(0.4, 0.6, 0.4)	(0.1, 0.9, 0.1)	(0.2, 0.8, 0.2)	(0.7, 0.3, 0.3)	(0.6, 0.4, 0.4)
STRA8	(0.7, 0.3, 0.3)	(0.3, 0.7, 0.3)	(0.6, 0.4, 0.4)	(0.4, 0.6, 0.4)	(0.1, 0.9, 0.1)	(0.2, 0.8, 0.2)	(0.7, 0.3, 0.3)	(0.6, 0.4, 0.4)	(0.7, 0.3, 0.3)	(0.6, 0.4, 0.4)	(0.3, 0.7, 0.3)	(0.7, 0.3, 0.3)	(0.3, 0.7, 0.3)	(0.6, 0.4, 0.4)	(0.4, 0.6, 0.4)	(0.1, 0.9, 0.1)	(0.2, 0.8, 0.2)	(0.7, 0.3, 0.3)	(0.6, 0.4, 0.4)	(0.6, 0.4, 0.4)
STRA9	(0.4, 0.6, 0.4)	(0.7, 0.3, 0.3)	(0.3, 0.7, 0.3)	(0.6, 0.4, 0.4)	(0.4, 0.6, 0.4)	(0.1, 0.9, 0.1)	(0.2, 0.8, 0.2)	(0.7, 0.3, 0.3)	(0.6, 0.4, 0.4)	(0.3, 0.7, 0.3)	(0.6, 0.4, 0.4)	(0.4, 0.6, 0.4)	(0.1, 0.9, 0.1)	(0.2, 0.8, 0.2)	(0.7, 0.3, 0.3)	(0.6, 0.4, 0.4)	(0.2, 0.8, 0.2)	(0.7, 0.3, 0.3)	(0.6, 0.4, 0.4)	(0.9, 0.1, 0.1)
STRA10	(0.8, 0.2, 0.2)	(0.8, 0.2, 0.2)	(0.9, 0.1, 0.1)	(0.4, 0.6, 0.4)	(0.3, 0.7, 0.3)	(0.8, 0.2, 0.2)	(0.9, 0.1, 0.1)	(0.8, 0.2, 0.2)	(0.8, 0.2, 0.2)	(0.9, 0.1, 0.1)	(0.4, 0.6, 0.4)	(0.3, 0.7, 0.3)	(0.3, 0.7, 0.3)	(0.6, 0.4, 0.4)	(0.8, 0.2, 0.2)	(0.9, 0.1, 0.1)	(0.4, 0.6, 0.4)	(0.3, 0.7, 0.3)	(0.3, 0.7, 0.3)	(0.1, 0.9, 0.1)
OPP1	(0.8, 0.2, 0.2)	(0.9, 0.1, 0.1)	(0.7, 0.3, 0.3)	(0.3, 0.7, 0.3)	(0.6, 0.4, 0.4)	(0.4, 0.6, 0.4)	(0.1, 0.9, 0.1)	(0.2, 0.8, 0.2)	(0.7, 0.3, 0.3)	(0.6, 0.4, 0.4)	(0.1, 0.9, 0.1)	(0.8, 0.2, 0.2)	(0.9, 0.1, 0.1)	(0.1, 0.9, 0.1)	(0.4, 0.6, 0.4)	(0.4, 0.6, 0.4)	(0.3, 0.7, 0.3)	(0.8, 0.2, 0.2)	(0.3, 0.7, 0.3)	(0.1, 0.9, 0.1)
OPP2	(0.8, 0.2, 0.2)	(0.9, 0.1, 0.1)	(0.4, 0.6, 0.4)	(0.3, 0.7, 0.3)	(0.6, 0.4, 0.4)	(0.8, 0.2, 0.2)	(0.9, 0.1, 0.1)	(0.4, 0.6, 0.4)	(0.7, 0.3, 0.3)	(0.3, 0.7, 0.3)	(0.6, 0.4, 0.4)	(0.4, 0.6, 0.4)	(0.1, 0.9, 0.1)	(0.2, 0.8, 0.2)	(0.7, 0.3, 0.3)	(0.6, 0.4, 0.4)	(0.8, 0.2, 0.2)	(0.1, 0.9, 0.1)	(0.2, 0.8, 0.2)	(0.6, 0.4, 0.4)
OPP3	(0.8, 0.2, 0.2)	(0.9, 0.1, 0.1)	(0.4, 0.6, 0.4)	(0.3, 0.7, 0.3)	(0.8, 0.2, 0.2)	(0.9, 0.1, 0.1)	(0.4, 0.6, 0.4)	(0.3, 0.7, 0.3)	(0.6, 0.4, 0.4)	(0.7, 0.3, 0.3)	(0.4, 0.6, 0.4)	(0.8, 0.2, 0.2)	(0.7, 0.3, 0.3)	(0.4, 0.6, 0.4)	(0.8, 0.2, 0.2)	(0.9, 0.1, 0.1)	(0.4, 0.6, 0.4)	(0.3, 0.7, 0.3)	(0.8, 0.2, 0.2)	(0.9, 0.1, 0.1)
OPP4	(0.9, 0.1, 0.1)	(0.8, 0.2, 0.2)	(0.7, 0.3, 0.3)	(0.3, 0.7, 0.3)	(0.6, 0.4, 0.4)	(0.4, 0.6, 0.4)	(0.1, 0.9, 0.1)	(0.2, 0.8, 0.2)	(0.7, 0.3, 0.3)	(0.6, 0.4, 0.4)	(0.6, 0.4, 0.4)	(0.7, 0.3, 0.3)	(0.8, 0.2, 0.2)	(0.9, 0.1, 0.1)	(0.4, 0.6, 0.4)	(0.3, 0.7, 0.3)	(0.6, 0.4, 0.4)	(0.7, 0.3, 0.3)	(0.4, 0.6, 0.4)	(0.8, 0.2, 0.2)
OPP5	(0.6, 0.4, 0.4)	(0.7, 0.3, 0.3)	(0.4, 0.6, 0.4)	(0.8, 0.2, 0.2)	(0.6, 0.4, 0.4)	(0.7, 0.3, 0.3)	(0.4, 0.6, 0.4)	(0.6, 0.4, 0.4)	(0.7, 0.3, 0.3)	(0.7, 0.3, 0.3)	(0.3, 0.7, 0.3)	(0.6, 0.4, 0.4)	(0.4, 0.6, 0.4)	(0.1, 0.9, 0.1)	(0.2, 0.8, 0.2)	(0.7, 0.3, 0.3)	(0.6, 0.4, 0.4)	(0.8, 0.2, 0.2)	(0.4, 0.6, 0.4)	(0.8, 0.2, 0.2)
OPP6	(0.7, 0.3, 0.3)	(0.3, 0.7, 0.3)	(0.6, 0.4, 0.4)	(0.4, 0.6, 0.4)	(0.1, 0.9, 0.1)	(0.2, 0.8, 0.2)	(0.7, 0.3, 0.3)	(0.6, 0.4, 0.4)	(0.6, 0.4, 0.4)	(0.7, 0.3, 0.3)	(0.6, 0.4, 0.4)	(0.7, 0.3, 0.3)	(0.4, 0.6, 0.4)	(0.8, 0.2, 0.2)	(0.4, 0.6, 0.4)	(0.6, 0.4, 0.4)	(0.7, 0.3, 0.3)	(0.4, 0.6, 0.4)	(0.8, 0.2, 0.2)	(0.8, 0.2, 0.2)
OPP7	(0.3, 0.7, 0.3)	(0.6, 0.4, 0.4)	(0.7, 0.3, 0.3)	(0.3, 0.7, 0.3)	(0.6, 0.4, 0.4)	(0.4, 0.6, 0.4)	(0.1, 0.9, 0.1)	(0.2, 0.8, 0.2)	(0.7, 0.3, 0.3)	(0.6, 0.4, 0.4)	(0.8, 0.2, 0.2)	(0.9, 0.1, 0.1)	(0.7, 0.3, 0.3)	(0.3, 0.7, 0.3)	(0.6, 0.4, 0.4)	(0.4, 0.6, 0.4)	(0.1, 0.9, 0.1)	(0.2, 0.8, 0.2)	(0.7, 0.3, 0.3)	(0.6, 0.4, 0.4)
OPP8	(0.6, 0.4, 0.4)	(0.7, 0.3, 0.3)	(0.3, 0.7, 0.3)	(0.6, 0.4, 0.4)	(0.4, 0.6, 0.4)	(0.1, 0.9, 0.1)	(0.2, 0.8, 0.2)	(0.7, 0.3, 0.3)	(0.6, 0.4, 0.4)	(0.4, 0.6, 0.4)	(0.1, 0.9, 0.1)	(0.9, 0.1, 0.1)	(0.7, 0.3, 0.3)	(0.3, 0.7, 0.3)	(0.6, 0.4, 0.4)	(0.4, 0.6, 0.4)	(0.1, 0.9, 0.1)	(0.2, 0.8, 0.2)	(0.7, 0.3, 0.3)	(0.6, 0.4, 0.4)
OPP9	(0.6, 0.4, 0.4)	(0.7, 0.3, 0.3)	(0.3, 0.7, 0.3)	(0.6, 0.4, 0.4)	(0.4, 0.6, 0.4)	(0.1, 0.9, 0.1)	(0.2, 0.8, 0.2)	(0.7, 0.3, 0.3)	(0.6, 0.4, 0.4)	(0.7, 0.3, 0.3)	(0.4, 0.6, 0.4)	(0.7, 0.3, 0.3)	(0.3, 0.7, 0.3)	(0.6, 0.4, 0.4)	(0.4, 0.6, 0.4)	(0.1, 0.9, 0.1)	(0.2, 0.8, 0.2)	(0.7, 0.3, 0.3)	(0.6, 0.4, 0.4)	(0.8, 0.2, 0.2)
OPP10	(0.8, 0.2, 0.2)	(0.6, 0.4, 0.4)	(0.6, 0.4, 0.4)	(0.7, 0.3, 0.3)	(0.4, 0.6, 0.4)	(0.8, 0.2, 0.2)	(0.3, 0.7, 0.3)	(0.6, 0.4, 0.4)	(0.7, 0.3, 0.3)	(0.4, 0.6, 0.4)	(0.6, 0.4, 0.4)	(0.7, 0.3, 0.3)	(0.3, 0.7, 0.3)	(0.6, 0.4, 0.4)	(0.4, 0.6, 0.4)	(0.1, 0.9, 0.1)	(0.2, 0.8, 0.2)	(0.7, 0.3, 0.3)	(0.6, 0.4, 0.4)	(0.6, 0.4, 0.4)
OPP11	(0.4, 0.6, 0.4)	(0.6, 0.4, 0.4)	(0.7, 0.3, 0.3)	(0.3, 0.7, 0.3)	(0.6, 0.4, 0.4)	(0.4, 0.6, 0.4)	(0.1, 0.9, 0.1)	(0.2, 0.8, 0.2)	(0.7, 0.3, 0.3)	(0.6, 0.4, 0.4)	(0.9, 0.1, 0.1)	(0.4, 0.6, 0.4)	(0.3, 0.7, 0.3)	(0.8, 0.2, 0.2)	(0.8, 0.2, 0.2)	(0.9, 0.1, 0.1)	(0.4, 0.6, 0.4)	(0.3, 0.7, 0.3)	(0.8, 0.2, 0.2)	(0.9, 0.1, 0.1)
WEA1	(0.6, 0.4, 0.4)	(0.7, 0.3, 0.3)	(0.4, 0.6, 0.4)	(0.6, 0.4, 0.4)	(0.7, 0.3, 0.3)	(0.6, 0.4, 0.4)	(0.7, 0.3, 0.3)	(0.4, 0.6, 0.4)	(0.8, 0.2, 0.2)	(0.7, 0.3, 0.3)	(0.3, 0.7, 0.3)	(0.6, 0.4, 0.4)	(0.4, 0.6, 0.4)	(0.1, 0.9, 0.1)	(0.2, 0.8, 0.2)	(0.7, 0.3, 0.3)	(0.6, 0.4, 0.4)	(0.8, 0.2, 0.2)	(0.3, 0.7, 0.3)	(0.1, 0.9, 0.1)
WEA2	(0.6, 0.4, 0.4)	(0.8, 0.2, 0.2)	(0.9, 0.1, 0.1)	(0.4, 0.6, 0.4)	(0.7, 0.3, 0.3)	(0.3, 0.7, 0.3)	(0.6, 0.4, 0.4)	(0.4, 0.6, 0.4)	(0.1, 0.9, 0.1)	(0.2, 0.8, 0.2)	(0.7, 0.3, 0.3)	(0.6, 0.4, 0.4)	(0.9, 0.1, 0.1)	(0.1, 0.9, 0.1)	(0.8, 0.2, 0.2)	(0.9, 0.1, 0.1)	(0.4, 0.6, 0.4)	(0.3, 0.7, 0.3)	(0.3, 0.7, 0.3)	(0.1, 0.9, 0.1)
WEA3	(0.8, 0.2, 0.2)	(0.1, 0.9, 0.1)	(0.2, 0.8, 0.2)	(0.6, 0.4, 0.4)	(0.7, 0.3, 0.3)	(0.3, 0.7, 0.3)	(0.6, 0.4, 0.4)	(0.4, 0.6, 0.4)	(0.1, 0.9, 0.1)	(0.2, 0.8, 0.2)	(0.7, 0.3, 0.3)	(0.6, 0.4, 0.4)	(0.1, 0.9, 0.1)	(0.2, 0.8, 0.2)	(0.6, 0.4, 0.4)	(0.8, 0.2, 0.2)	(0.9, 0.1, 0.1)	(0.4, 0.6, 0.4)	(0.3, 0.7, 0.3)	(0.8, 0.2, 0.2)
WEA4	(0.9, 0.1, 0.1)	(0.8, 0.2, 0.2)	(0.9, 0.1, 0.1)	(0.4, 0.6, 0.4)	(0.8, 0.2, 0.2)	(0.7, 0.3, 0.3)	(0.3, 0.7, 0.3)	(0.6, 0.4, 0.4)	(0.4, 0.6, 0.4)	(0.1, 0.9, 0.1)	(0.2, 0.8, 0.2)	(0.7, 0.3, 0.3)	(0.3, 0.7, 0.3)	(0.6, 0.4, 0.4)	(0.4, 0.6, 0.4)	(0.1, 0.9, 0.1)	(0.2, 0.8, 0.2)	(0.7, 0.3, 0.3)	(0.6, 0.4, 0.4)	(0.7, 0.3, 0.3)
WEA5	(0.7, 0.3, 0.3)	(0.3, 0.7, 0.3)	(0.6, 0.4, 0.4)	(0.4, 0.6, 0.4)	(0.1, 0.9, 0.1)	(0.2, 0.8, 0.2)	(0.7, 0.3, 0.3)	(0.6, 0.4, 0.4)	(0.4, 0.6, 0.4)	(0.3, 0.7, 0.3)	(0.9, 0.1, 0.1)	(0.7, 0.3, 0.3)	(0.3, 0.7, 0.3)	(0.6, 0.4, 0.4)	(0.4, 0.6, 0.4)	(0.1, 0.9, 0.1)	(0.2, 0.8, 0.2)	(0.7, 0.3, 0.3)	(0.6, 0.4, 0.4)	(0.8, 0.2, 0.2)
WEA6	(0.8, 0.2, 0.2)	(0.7, 0.3, 0.3)	(0.3, 0.7, 0.3)	(0.6, 0.4, 0.4)	(0.4, 0.6, 0.4)	(0.1, 0.9, 0.1)	(0.2, 0.8, 0.2)	(0.7, 0.3, 0.3)	(0.6, 0.4, 0.4)	(0.7, 0.3, 0.3)	(0.9, 0.1, 0.1)	(0.7, 0.3, 0.3)	(0.3, 0.7, 0.3)	(0.6, 0.4, 0.4)	(0.4, 0.6, 0.4)	(0.1, 0.9, 0.1)	(0.2, 0.8, 0.2)	(0.7, 0.3, 0.3)	(0.6, 0.4, 0.4)	(0.4, 0.6, 0.4)
THR1	(0.9, 0.1, 0.1)	(0.4, 0.6, 0.4)	(0.7, 0.3, 0.3)	(0.9, 0.1, 0.1)	(0.4, 0.6, 0.4)	(0.7, 0.3, 0.3)	(0.3, 0.7, 0.3)	(0.6, 0.4, 0.4)	(0.4, 0.6, 0.4)	(0.1, 0.9, 0.1)	(0.2, 0.8, 0.2)	(0.7, 0.3, 0.3)	(0.6, 0.4, 0.4)	(0.6, 0.4, 0.4)	(0.4, 0.6, 0.4)	(0.8, 0.2, 0.2)	(0.4, 0.6, 0.4)	(0.4, 0.6, 0.4)	(0.3, 0.7, 0.3)	(0.8, 0.2, 0.2)
THR2	(0.1, 0.9, 0.1)	(0.7, 0.3, 0.3)	(0.3, 0.7, 0.3)	(0.6, 0.4, 0.4)	(0.4, 0.6, 0.4)	(0.1, 0.9, 0.1)	(0.2, 0.8, 0.2)	(0.7, 0.3, 0.3)	(0.6, 0.4, 0.4)	(0.7, 0.3, 0.3)	(0.9, 0.1, 0.1)	(0.4, 0.6, 0.4)	(0.7, 0.3, 0.3)	(0.8, 0.2, 0.2)	(0.4, 0.6, 0.4)	(0.7, 0.3, 0.3)	(0.9, 0.1, 0.1)	(0.4, 0.6, 0.4)	(0.7, 0.3, 0.3)	(0.4, 0.6, 0.4)
THR3	(0.9, 0.1, 0.1)	(0.7, 0.3, 0.3)	(0.3, 0.7, 0.3)	(0.6, 0.4, 0.4)	(0.4, 0.6, 0.4)	(0.1, 0.9, 0.1)	(0.2, 0.8, 0.2)	(0.7, 0.3, 0.3)	(0.6, 0.4, 0.4)	(0.4, 0.6, 0.4)	(0.1, 0.9, 0.1)	(0.9, 0.1, 0.1)	(0.8, 0.2, 0.2)	(0.4, 0.6, 0.4)	(0.4, 0.6, 0.4)	(0.3, 0.7, 0.3)	(0.8, 0.2, 0.2)	(0.6, 0.4, 0.4)	(0.9, 0.1, 0.1)	(0.4, 0.6, 0.4)
THR4	(0.8, 0.2, 0.2)	(0.4, 0.6, 0.4)	(0.7, 0.3, 0.3)	(0.3, 0.7, 0.3)	(0.6, 0.4, 0.4)	(0.4, 0.6, 0.4)	(0.1, 0.9, 0.1)	(0.2, 0.8, 0.2)	(0.7, 0.3, 0.3)	(0.6, 0.4, 0.4)	(0.9, 0.1, 0.1)	(0.4, 0.6, 0.4)	(0.9, 0.1, 0.1)	(0.8, 0.2, 0.2)	(0.4, 0.6, 0.4)	(0.7, 0.3, 0.3)	(0.9, 0.1, 0.1)	(0.4, 0.6, 0.4)	(0.7, 0.3, 0.3)	(0.8, 0.2, 0.2)
THR5	(0.8, 0.2, 0.2)	(0.4, 0.6, 0.4)	(0.7, 0.3, 0.3)	(0.7, 0.3, 0.3)	(0.3, 0.7, 0.3)	(0.6, 0.4, 0.4)	(0.4, 0.6, 0.4)	(0.1, 0.9, 0.1)	(0.2, 0.8, 0.2)	(0.7, 0.3, 0.3)	(0.3, 0.7, 0.3)	(0.6, 0.4, 0.4)	(0.4, 0.6, 0.4)	(0.1, 0.9, 0.1)	(0.2, 0.8, 0.2)	(0.7, 0.3, 0.3)	(0.6, 0.4, 0.4)	(0.7, 0.3, 0.3)	(0.9, 0.1, 0.1)	(0.4, 0.6, 0.4)
THR6	(0.7, 0.3, 0.3)	(0.3, 0.7, 0.3)	(0.6, 0.4, 0.4)	(0.4, 0.6, 0.4)	(0.1, 0.9, 0.1)	(0.2, 0.8, 0.2)	(0.7, 0.3, 0.3)	(0.6, 0.4, 0.4)	(0.8, 0.2, 0.2)	(0.7, 0.3, 0.3)	(0.3, 0.7, 0.3)	(0.6, 0.4, 0.4)	(0.4, 0.6, 0.4)	(0.1, 0.9, 0.1)	(0.2, 0.8, 0.2)	(0.7, 0.3, 0.3)	(0.6, 0.4, 0.4)	(0.4, 0.6, 0.4)	(0.7, 0.3, 0.3)	(0.9, 0.1, 0.1)
THR7	(0.9, 0.1, 0.1)	(0.7, 0.3, 0.3)	(0.3, 0.7, 0.3)	(0.6, 0.4, 0.4)	(0.4, 0.6, 0.4)	(0.1, 0.9, 0.1)	(0.2, 0.8, 0.2)	(0.7, 0.3, 0.3)	(0.3, 0.7, 0.3)	(0.6, 0.4, 0.4)	(0.4, 0.6, 0.4)	(0.1, 0.9, 0.1)	(0.2, 0.8, 0.2)	(0.7, 0.3, 0.3)	(0.6, 0.4, 0.4)	(0.9, 0.1, 0.1)	(0.4, 0.6, 0.4)	(0.7, 0.3, 0.3)	(0.3, 0.7, 0.3)	(0.1, 0.9, 0.1)
THR8	(0.4, 0.6, 0.4)	(0.7, 0.3, 0.3)	(0.9, 0.1, 0.1)	(0.7, 0.3, 0.3)	(0.3, 0.7, 0.3)	(0.6, 0.4, 0.4)	(0.4, 0.6, 0.4)	(0.1, 0.9, 0.1)	(0.2, 0.8, 0.2)	(0.7, 0.3, 0.3)	(0.6, 0.4, 0.4)	(0.1, 0.9, 0.1)	(0.2, 0.8, 0.2)	(0.7, 0.3, 0.3)	(0.6, 0.4, 0.4)	(0.7, 0.3, 0.3)	(0.9, 0.1, 0.1)	(0.4, 0.6, 0.4)	(0.7, 0.3, 0.3)	(0.1, 0.9, 0.1)
THR9	(0.9, 0.1, 0.1)	(0.8, 0.2, 0.2)	(0.9, 0.1, 0.1)	(0.7, 0.3, 0.3)	(0.3, 0.7, 0.3)	(0.6, 0.4, 0.4)	(0.4, 0.6, 0.4)	(0.1, 0.9, 0.1)	(0.2, 0.8, 0.2)	(0.7, 0.3, 0.3)	(0.6, 0.4, 0.4)	(0.8, 0.2, 0.2)	(0.9, 0.1, 0.1)	(0.4, 0.6, 0.4)	(0.3, 0.7, 0.3)	(0.1, 0.9, 0.1)	(0.2, 0.8, 0.2)	(0.6, 0.4, 0.4)	(0.3, 0.7, 0.3)	(0.8, 0.2, 0.2)
THR10	(0.9, 0.1, 0.1)	(0.4, 0.6, 0.4)	(0.8, 0.2, 0.2)	(0.9, 0.1, 0.1)	(0.7, 0.3, 0.3)	(0.3, 0.7, 0.3)	(0.6, 0.4, 0.4)	(0.4, 0.6, 0.4)	(0.1, 0.9, 0.1)	(0.2, 0.8, 0.2)	(0.7, 0.3, 0.3)	(0.6, 0.4, 0.4)	(0.9, 0.1, 0.1)	(0.4, 0.6, 0.4)	(0.3, 0.7, 0.3)	(0.4, 0.6, 0.4)	(0.4, 0.6, 0.4)	(0.3, 0.7, 0.3)	(0.8, 0.2, 0.2)	(0.9, 0.1, 0.1)

Table A5. The decision matrix between overall criteria and alternatives by the GRA method for fifth experts.

	GME1	GME2	GME3	GME4	GME5	GME6	GME7	GME8	GME9	GME10	GME11	GME12	GME13	GME14	GME15	GME16	GME17	GME18	GME19	GME20
STRA1	(0.6, 0.4, 0.4)	(0.9, 0.1, 0.1)	(0.9, 0.1, 0.1)	(0.8, 0.2, 0.2)	(0.1, 0.9, 0.1)	(0.2, 0.8, 0.2)	(0.6, 0.4, 0.4)	(0.3, 0.7, 0.3)	(0.3, 0.7, 0.3)	(0.8, 0.2, 0.2)	(0.9, 0.1, 0.1)	(0.6, 0.4, 0.4)	(0.9, 0.1, 0.1)	(0.9, 0.1, 0.1)	(0.8, 0.2, 0.2)	(0.1, 0.9, 0.1)	(0.2, 0.8, 0.2)	(0.6, 0.4, 0.4)	(0.3, 0.7, 0.3)	(0.9, 0.1, 0.1)
STRA2	(0.8, 0.2, 0.2)	(0.9, 0.1, 0.1)	(0.4, 0.6, 0.4)	(0.3, 0.7, 0.3)	(0.2, 0.8, 0.2)	(0.4, 0.6, 0.4)	(0.8, 0.2, 0.2)	(0.4, 0.6, 0.4)	(0.1, 0.9, 0.1)	(0.4, 0.6, 0.4)	(0.9, 0.1, 0.1)	(0.2, 0.8, 0.2)	(0.4, 0.6, 0.4)	(0.6, 0.4, 0.4)	(0.9, 0.1, 0.1)	(0.9, 0.1, 0.1)	(0.8, 0.2, 0.2)	(0.1, 0.9, 0.1)	(0.2, 0.8, 0.2)	(0.6, 0.4, 0.4)
STRA3	(0.8, 0.2, 0.2)	(0.9, 0.1, 0.1)	(0.4, 0.6, 0.4)	(0.3, 0.7, 0.3)	(0.6, 0.4, 0.4)	(0.9, 0.1, 0.1)	(0.9, 0.1, 0.1)	(0.8, 0.2, 0.2)	(0.1, 0.9, 0.1)	(0.2, 0.8, 0.2)	(0.6, 0.4, 0.4)	(0.3, 0.7, 0.3)	(0.1, 0.9, 0.1)	(0.6, 0.4, 0.4)	(0.9, 0.1, 0.1)	(0.9, 0.1, 0.1)	(0.8, 0.2, 0.2)	(0.1, 0.9, 0.1)	(0.2, 0.8, 0.2)	(0.6, 0.4, 0.4)
STRA4	(0.9, 0.1, 0.1)	(0.8, 0.2, 0.2)	(0.4, 0.6, 0.4)	(0.4, 0.6, 0.4)	(0.6, 0.4, 0.4)	(0.9, 0.1, 0.1)	(0.9, 0.1, 0.1)	(0.8, 0.2, 0.2)	(0.1, 0.9, 0.1)	(0.2, 0.8, 0.2)	(0.6, 0.4, 0.4)	(0.9, 0.1, 0.1)	(0.9, 0.1, 0.1)	(0.8, 0.2, 0.2)	(0.1, 0.9, 0.1)	(0.2, 0.8, 0.2)	(0.6, 0.4, 0.4)	(0.3, 0.7, 0.3)	(0.3, 0.7, 0.3)	(0.8, 0.2, 0.2)
STRA5	(0.1, 0.9, 0.1)	(0.6, 0.4, 0.4)	(0.9, 0.1, 0.1)	(0.9, 0.1, 0.1)	(0.8, 0.2, 0.2)	(0.1, 0.9, 0.1)	(0.2, 0.8, 0.2)	(0.6, 0.4, 0.4)	(0.3, 0.7, 0.3)	(0.8, 0.2, 0.2)	(0.6, 0.4, 0.4)	(0.9, 0.1, 0.1)	(0.9, 0.1, 0.1)	(0.8, 0.2, 0.2)	(0.1, 0.9, 0.1)	(0.2, 0.8, 0.2)	(0.6, 0.4, 0.4)	(0.3, 0.7, 0.3)	(0.9, 0.1, 0.1)	(0.4, 0.6, 0.4)
STRA6	(0.8, 0.2, 0.2)	(0.6, 0.4, 0.4)	(0.9, 0.1, 0.1)	(0.9, 0.1, 0.1)	(0.8, 0.2, 0.2)	(0.1, 0.9, 0.1)	(0.2, 0.8, 0.2)	(0.6, 0.4, 0.4)	(0.3, 0.7, 0.3)	(0.4, 0.6, 0.4)	(0.1, 0.9, 0.1)	(0.9, 0.1, 0.1)	(0.8, 0.2, 0.2)	(0.6, 0.4, 0.4)	(0.9, 0.1, 0.1)	(0.9, 0.1, 0.1)	(0.8, 0.2, 0.2)	(0.1, 0.9, 0.1)	(0.2, 0.8, 0.2)	(0.6, 0.4, 0.4)
STRA7	(0.6, 0.4, 0.4)	(0.9, 0.1, 0.1)	(0.9, 0.1, 0.1)	(0.8, 0.2, 0.2)	(0.1, 0.9, 0.1)	(0.2, 0.8, 0.2)	(0.6, 0.4, 0.4)	(0.3, 0.7, 0.3)	(0.8, 0.2, 0.2)	(0.6, 0.4, 0.4)	(0.9, 0.1, 0.1)	(0.4, 0.6, 0.4)	(0.9, 0.1, 0.1)	(0.6, 0.4, 0.4)	(0.9, 0.1, 0.1)	(0.9, 0.1, 0.1)	(0.8, 0.2, 0.2)	(0.1, 0.9, 0.1)	(0.2, 0.8, 0.2)	(0.6, 0.4, 0.4)
STRA8	(0.8, 0.2, 0.2)	(0.6, 0.4, 0.4)	(0.9, 0.1, 0.1)	(0.9, 0.1, 0.1)	(0.8, 0.2, 0.2)	(0.1, 0.9, 0.1)	(0.2, 0.8, 0.2)	(0.6, 0.4, 0.4)	(0.3, 0.7, 0.3)	(0.3, 0.7, 0.3)	(0.7, 0.3, 0.3)	(0.8, 0.2, 0.2)	(0.3, 0.7, 0.3)	(0.6, 0.4, 0.4)	(0.9, 0.1, 0.1)	(0.9, 0.1, 0.1)	(0.8, 0.2, 0.2)	(0.1, 0.9, 0.1)	(0.2, 0.8, 0.2)	(0.6, 0.4, 0.4)
STRA9	(0.4, 0.6, 0.4)	(0.4, 0.6, 0.4)	(0.6, 0.4, 0.4)	(0.9, 0.1, 0.1)	(0.9, 0.1, 0.1)	(0.8, 0.2, 0.2)	(0.1, 0.9, 0.1)	(0.2, 0.8, 0.2)	(0.6, 0.4, 0.4)	(0.3, 0.7, 0.3)	(0.8, 0.2, 0.2)	(0.1, 0.9, 0.1)	(0.2, 0.8, 0.2)	(0.6, 0.4, 0.4)	(0.3, 0.7, 0.3)	(0.4, 0.6, 0.4)	(0.4, 0.6, 0.4)	(0.3, 0.7, 0.3)	(0.8, 0.2, 0.2)	(0.9, 0.1, 0.1)
STRA10	(0.8, 0.2, 0.2)	(0.8, 0.2, 0.2)	(0.6, 0.4, 0.4)	(0.9, 0.1, 0.1)	(0.9, 0.1, 0.1)	(0.8, 0.2, 0.2)	(0.1, 0.9, 0.1)	(0.2, 0.8, 0.2)	(0.6, 0.4, 0.4)	(0.3, 0.7, 0.3)	(0.4, 0.6, 0.4)	(0.6, 0.4, 0.4)	(0.9, 0.1, 0.1)	(0.9, 0.1, 0.1)	(0.8, 0.2, 0.2)	(0.1, 0.9, 0.1)	(0.2, 0.8, 0.2)	(0.6, 0.4, 0.4)	(0.3, 0.7, 0.3)	(0.1, 0.9, 0.1)
OPP1	(0.8, 0.2, 0.2)	(0.9, 0.1, 0.1)	(0.4, 0.6, 0.4)	(0.3, 0.7, 0.3)	(0.3, 0.7, 0.3)	(0.8, 0.2, 0.2)	(0.9, 0.1, 0.1)	(0.4, 0.6, 0.4)	(0.3, 0.7, 0.3)	(0.8, 0.2, 0.2)	(0.1, 0.9, 0.1)	(0.8, 0.2, 0.2)	(0.9, 0.1, 0.1)	(0.1, 0.9, 0.1)	(0.4, 0.6, 0.4)	(0.4, 0.6, 0.4)	(0.3, 0.7, 0.3)	(0.8, 0.2, 0.2)	(0.3, 0.7, 0.3)	(0.1, 0.9, 0.1)
OPP2	(0.8, 0.2, 0.2)	(0.9, 0.1, 0.1)	(0.4, 0.6, 0.4)	(0.3, 0.7, 0.3)	(0.6, 0.4, 0.4)	(0.8, 0.2, 0.2)	(0.9, 0.1, 0.1)	(0.4, 0.6, 0.4)	(0.3, 0.7, 0.3)	(0.4, 0.6, 0.4)	(0.4, 0.6, 0.4)	(0.6, 0.4, 0.4)	(0.9, 0.1, 0.1)	(0.9, 0.1, 0.1)	(0.8, 0.2, 0.2)	(0.1, 0.9, 0.1)	(0.2, 0.8, 0.2)	(0.6, 0.4, 0.4)	(0.3, 0.7, 0.3)	(0.6, 0.4, 0.4)
OPP3	(0.8, 0.2, 0.2)	(0.6, 0.4, 0.4)	(0.9, 0.1, 0.1)	(0.9, 0.1, 0.1)	(0.8, 0.2, 0.2)	(0.1, 0.9, 0.1)	(0.2, 0.8, 0.2)	(0.6, 0.4, 0.4)	(0.3, 0.7, 0.3)	(0.7, 0.3, 0.3)	(0.4, 0.6, 0.4)	(0.8, 0.2, 0.2)	(0.7, 0.3, 0.3)	(0.4, 0.6, 0.4)	(0.8, 0.2, 0.2)	(0.9, 0.1, 0.1)	(0.4, 0.6, 0.4)	(0.3, 0.7, 0.3)	(0.8, 0.2, 0.2)	(0.9, 0.1, 0.1)
OPP4	(0.9, 0.1, 0.1)	(0.6, 0.4, 0.4)	(0.9, 0.1, 0.1)	(0.9, 0.1, 0.1)	(0.8, 0.2, 0.2)	(0.1, 0.9, 0.1)	(0.2, 0.8, 0.2)	(0.6, 0.4, 0.4)	(0.3, 0.7, 0.3)	(0.4, 0.6, 0.4)	(0.6, 0.4, 0.4)	(0.7, 0.3, 0.3)	(0.8, 0.2, 0.2)	(0.9, 0.1, 0.1)	(0.4, 0.6, 0.4)	(0.3, 0.7, 0.3)	(0.6, 0.4, 0.4)	(0.7, 0.3, 0.3)	(0.4, 0.6, 0.4)	(0.8, 0.2, 0.2)
OPP5	(0.6, 0.4, 0.4)	(0.7, 0.3, 0.3)	(0.4, 0.6, 0.4)	(0.8, 0.2, 0.2)	(0.6, 0.4, 0.4)	(0.7, 0.3, 0.3)	(0.4, 0.6, 0.4)	(0.6, 0.4, 0.4)	(0.9, 0.1, 0.1)	(0.9, 0.1, 0.1)	(0.8, 0.2, 0.2)	(0.1, 0.9, 0.1)	(0.2, 0.8, 0.2)	(0.6, 0.4, 0.4)	(0.3, 0.7, 0.3)	(0.7, 0.3, 0.3)	(0.4, 0.6, 0.4)	(0.8, 0.2, 0.2)	(0.4, 0.6, 0.4)	(0.8, 0.2, 0.2)
OPP6	(0.4, 0.6, 0.4)	(0.7, 0.3, 0.3)	(0.4, 0.6, 0.4)	(0.8, 0.2, 0.2)	(0.4, 0.6, 0.4)	(0.8, 0.2, 0.2)	(0.4, 0.6, 0.4)	(0.6, 0.4, 0.4)	(0.9, 0.1, 0.1)	(0.9, 0.1, 0.1)	(0.8, 0.2, 0.2)	(0.1, 0.9, 0.1)	(0.2, 0.8, 0.2)	(0.6, 0.4, 0.4)	(0.3, 0.7, 0.3)	(0.6, 0.4, 0.4)	(0.7, 0.3, 0.3)	(0.4, 0.6, 0.4)	(0.8, 0.2, 0.2)	(0.8, 0.2, 0.2)
OPP7	(0.3, 0.7, 0.3)	(0.6, 0.4, 0.4)	(0.7, 0.3, 0.3)	(0.4, 0.6, 0.4)	(0.8, 0.2, 0.2)	(0.4, 0.6, 0.4)	(0.3, 0.7, 0.3)	(0.6, 0.4, 0.4)	(0.7, 0.3, 0.3)	(0.4, 0.6, 0.4)	(0.8, 0.2, 0.2)	(0.6, 0.4, 0.4)	(0.9, 0.1, 0.1)	(0.9, 0.1, 0.1)	(0.8, 0.2, 0.2)	(0.1, 0.9, 0.1)	(0.2, 0.8, 0.2)	(0.6, 0.4, 0.4)	(0.3, 0.7, 0.3)	(0.4, 0.6, 0.4)
OPP8	(0.6, 0.4, 0.4)	(0.7, 0.3, 0.3)	(0.4, 0.6, 0.4)	(0.8, 0.2, 0.2)	(0.6, 0.4, 0.4)	(0.9, 0.1, 0.1)	(0.9, 0.1, 0.1)	(0.8, 0.2, 0.2)	(0.1, 0.9, 0.1)	(0.2, 0.8, 0.2)	(0.6, 0.4, 0.4)	(0.3, 0.7, 0.3)	(0.6, 0.4, 0.4)	(0.7, 0.3, 0.3)	(0.4, 0.6, 0.4)	(0.8, 0.2, 0.2)	(0.7, 0.3, 0.3)	(0.4, 0.6, 0.4)	(0.8, 0.2, 0.2)	(0.4, 0.6, 0.4)
OPP9	(0.6, 0.4, 0.4)	(0.7, 0.3, 0.3)	(0.4, 0.6, 0.4)	(0.8, 0.2, 0.2)	(0.6, 0.4, 0.4)	(0.7, 0.3, 0.3)	(0.6, 0.4, 0.4)	(0.9, 0.1, 0.1)	(0.9, 0.1, 0.1)	(0.8, 0.2, 0.2)	(0.1, 0.9, 0.1)	(0.2, 0.8, 0.2)	(0.6, 0.4, 0.4)	(0.3, 0.7, 0.3)	(0.9, 0.1, 0.1)	(0.6, 0.4, 0.4)	(0.7, 0.3, 0.3)	(0.4, 0.6, 0.4)	(0.8, 0.2, 0.2)	(0.8, 0.2, 0.2)
OPP10	(0.8, 0.2, 0.2)	(0.6, 0.4, 0.4)	(0.6, 0.4, 0.4)	(0.7, 0.3, 0.3)	(0.4, 0.6, 0.4)	(0.8, 0.2, 0.2)	(0.3, 0.7, 0.3)	(0.6, 0.4, 0.4)	(0.9, 0.1, 0.1)	(0.9, 0.1, 0.1)	(0.8, 0.2, 0.2)	(0.1, 0.9, 0.1)	(0.2, 0.8, 0.2)	(0.6, 0.4, 0.4)	(0.3, 0.7, 0.3)	(0.4, 0.6, 0.4)	(0.3, 0.7, 0.3)	(0.8, 0.2, 0.2)	(0.8, 0.2, 0.2)	(0.6, 0.4, 0.4)
OPP11	(0.6, 0.4, 0.4)	(0.9, 0.1, 0.1)	(0.9, 0.1, 0.1)	(0.8, 0.2, 0.2)	(0.1, 0.9, 0.1)	(0.2, 0.8, 0.2)	(0.6, 0.4, 0.4)	(0.3, 0.7, 0.3)	(0.7, 0.3, 0.3)	(0.8, 0.2, 0.2)	(0.9, 0.1, 0.1)	(0.4, 0.6, 0.4)	(0.3, 0.7, 0.3)	(0.8, 0.2, 0.2)	(0.8, 0.2, 0.2)	(0.9, 0.1, 0.1)	(0.4, 0.6, 0.4)	(0.3, 0.7, 0.3)	(0.8, 0.2, 0.2)	(0.9, 0.1, 0.1)
WEA1	(0.6, 0.4, 0.4)	(0.9, 0.1, 0.1)	(0.9, 0.1, 0.1)	(0.8, 0.2, 0.2)	(0.1, 0.9, 0.1)	(0.2, 0.8, 0.2)	(0.6, 0.4, 0.4)	(0.3, 0.7, 0.3)	(0.8, 0.2, 0.2)	(0.6, 0.4, 0.4)	(0.9, 0.1, 0.1)	(0.9, 0.1, 0.1)	(0.8, 0.2, 0.2)	(0.1, 0.9, 0.1)	(0.2, 0.8, 0.2)	(0.6, 0.4, 0.4)	(0.3, 0.7, 0.3)	(0.8, 0.2, 0.2)	(0.3, 0.7, 0.3)	(0.1, 0.9, 0.1)
WEA2	(0.6, 0.4, 0.4)	(0.8, 0.2, 0.2)	(0.9, 0.1, 0.1)	(0.4, 0.6, 0.4)	(0.3, 0.7, 0.3)	(0.4, 0.6, 0.4)	(0.8, 0.2, 0.2)	(0.9, 0.1, 0.1)	(0.1, 0.9, 0.1)	(0.6, 0.4, 0.4)	(0.9, 0.1, 0.1)	(0.9, 0.1, 0.1)	(0.8, 0.2, 0.2)	(0.1, 0.9, 0.1)	(0.2, 0.8, 0.2)	(0.6, 0.4, 0.4)	(0.3, 0.7, 0.3)	(0.3, 0.7, 0.3)	(0.3, 0.7, 0.3)	(0.1, 0.9, 0.1)
WEA3	(0.8, 0.2, 0.2)	(0.6, 0.4, 0.4)	(0.9, 0.1, 0.1)	(0.9, 0.1, 0.1)	(0.8, 0.2, 0.2)	(0.1, 0.9, 0.1)	(0.2, 0.8, 0.2)	(0.6, 0.4, 0.4)	(0.3, 0.7, 0.3)	(0.4, 0.6, 0.4)	(0.3, 0.7, 0.3)	(0.8, 0.2, 0.2)	(0.1, 0.9, 0.1)	(0.2, 0.8, 0.2)	(0.6, 0.4, 0.4)	(0.8, 0.2, 0.2)	(0.9, 0.1, 0.1)	(0.4, 0.6, 0.4)	(0.3, 0.7, 0.3)	(0.8, 0.2, 0.2)
WEA4	(0.9, 0.1, 0.1)	(0.8, 0.2, 0.2)	(0.9, 0.1, 0.1)	(0.4, 0.6, 0.4)	(0.8, 0.2, 0.2)	(0.9, 0.1, 0.1)	(0.4, 0.6, 0.4)	(0.3, 0.7, 0.3)	(0.9, 0.1, 0.1)	(0.4, 0.6, 0.4)	(0.7, 0.3, 0.3)	(0.8, 0.2, 0.2)	(0.9, 0.1, 0.1)	(0.4, 0.6, 0.4)	(0.3, 0.7, 0.3)	(0.4, 0.6, 0.4)	(0.7, 0.3, 0.3)	(0.9, 0.1, 0.1)	(0.4, 0.6, 0.4)	(0.7, 0.3, 0.3)
WEA5	(0.6, 0.4, 0.4)	(0.9, 0.1, 0.1)	(0.9, 0.1, 0.1)	(0.8, 0.2, 0.2)	(0.1, 0.9, 0.1)	(0.2, 0.8, 0.2)	(0.6, 0.4, 0.4)	(0.3, 0.7, 0.3)	(0.4, 0.6, 0.4)	(0.3, 0.7, 0.3)	(0.9, 0.1, 0.1)	(0.2, 0.8, 0.2)	(0.4, 0.6, 0.4)	(0.8, 0.2, 0.2)	(0.3, 0.7, 0.3)	(0.6, 0.4, 0.4)	(0.4, 0.6, 0.4)	(0.4, 0.6, 0.4)	(0.3, 0.7, 0.3)	(0.8, 0.2, 0.2)
WEA6	(0.8, 0.2, 0.2)	(0.4, 0.6, 0.4)	(0.7, 0.3, 0.3)	(0.9, 0.1, 0.1)	(0.4, 0.6, 0.4)	(0.7, 0.3, 0.3)	(0.4, 0.6, 0.4)	(0.7, 0.3, 0.3)	(0.4, 0.6, 0.4)	(0.7, 0.3, 0.3)	(0.9, 0.1, 0.1)	(0.4, 0.6, 0.4)	(0.7, 0.3, 0.3)	(0.2, 0.8, 0.2)	(0.4, 0.6, 0.4)	(0.7, 0.3, 0.3)	(0.4, 0.6, 0.4)	(0.7, 0.3, 0.3)	(0.9, 0.1, 0.1)	(0.4, 0.6, 0.4)
THR1	(0.6, 0.4, 0.4)	(0.9, 0.1, 0.1)	(0.9, 0.1, 0.1)	(0.8, 0.2, 0.2)	(0.1, 0.9, 0.1)	(0.2, 0.8, 0.2)	(0.6, 0.4, 0.4)	(0.3, 0.7, 0.3)	(0.8, 0.2, 0.2)	(0.6, 0.4, 0.4)	(0.9, 0.1, 0.1)	(0.9, 0.1, 0.1)	(0.8, 0.2, 0.2)	(0.1, 0.9, 0.1)	(0.2, 0.8, 0.2)	(0.6, 0.4, 0.4)	(0.3, 0.7, 0.3)	(0.4, 0.6, 0.4)	(0.3, 0.7, 0.3)	(0.8, 0.2, 0.2)
THR2	(0.1, 0.9, 0.1)	(0.6, 0.4, 0.4)	(0.9, 0.1, 0.1)	(0.9, 0.1, 0.1)	(0.8, 0.2, 0.2)	(0.1, 0.9, 0.1)	(0.2, 0.8, 0.2)	(0.6, 0.4, 0.4)	(0.3, 0.7, 0.3)	(0.7, 0.3, 0.3)	(0.9, 0.1, 0.1)	(0.4, 0.6, 0.4)	(0.7, 0.3, 0.3)	(0.8, 0.2, 0.2)	(0.4, 0.6, 0.4)	(0.7, 0.3, 0.3)	(0.9, 0.1, 0.1)	(0.4, 0.6, 0.4)	(0.7, 0.3, 0.3)	(0.4, 0.6, 0.4)
THR3	(0.9, 0.1, 0.1)	(0.8, 0.2, 0.2)	(0.6, 0.4, 0.4)	(0.9, 0.1, 0.1)	(0.4, 0.6, 0.4)	(0.9, 0.1, 0.1)	(0.8, 0.2, 0.2)	(0.6, 0.4, 0.4)	(0.9, 0.1, 0.1)	(0.4, 0.6, 0.4)	(0.1, 0.9, 0.1)	(0.9, 0.1, 0.1)	(0.8, 0.2, 0.2)	(0.4, 0.6, 0.4)	(0.4, 0.6, 0.4)	(0.3, 0.7, 0.3)	(0.8, 0.2, 0.2)	(0.6, 0.4, 0.4)	(0.9, 0.1, 0.1)	(0.4, 0.6, 0.4)
THR4	(0.8, 0.2, 0.2)	(0.6, 0.4, 0.4)	(0.9, 0.1, 0.1)	(0.9, 0.1, 0.1)	(0.8, 0.2, 0.2)	(0.1, 0.9, 0.1)	(0.2, 0.8, 0.2)	(0.6, 0.4, 0.4)	(0.3, 0.7, 0.3)	(0.6, 0.4, 0.4)	(0.9, 0.1, 0.1)	(0.4, 0.6, 0.4)	(0.9, 0.1, 0.1)	(0.8, 0.2, 0.2)	(0.4, 0.6, 0.4)	(0.7, 0.3, 0.3)	(0.9, 0.1, 0.1)	(0.4, 0.6, 0.4)	(0.7, 0.3, 0.3)	(0.8, 0.2, 0.2)
THR5	(0.6, 0.4, 0.4)	(0.9, 0.1, 0.1)	(0.9, 0.1, 0.1)	(0.6, 0.4, 0.4)	(0.9, 0.1, 0.1)	(0.9, 0.1, 0.1)	(0.8, 0.2, 0.2)	(0.1, 0.9, 0.1)	(0.2, 0.8, 0.2)	(0.6, 0.4, 0.4)	(0.3, 0.7, 0.3)	(0.8, 0.2, 0.2)	(0.3, 0.7, 0.3)	(0.6, 0.4, 0.4)	(0.4, 0.6, 0.4)	(0.4, 0.6, 0.4)	(0.4, 0.6, 0.4)	(0.7, 0.3, 0.3)	(0.9, 0.1, 0.1)	(0.4, 0.6, 0.4)
THR6	(0.4, 0.6, 0.4)	(0.4, 0.6, 0.4)	(0.4, 0.6, 0.4)	(0.6, 0.4, 0.4)	(0.9, 0.1, 0.1)	(0.9, 0.1, 0.1)	(0.8, 0.2, 0.2)	(0.1, 0.9, 0.1)	(0.2, 0.8, 0.2)	(0.6, 0.4, 0.4)	(0.3, 0.7, 0.3)	(0.6, 0.4, 0.4)	(0.3, 0.7, 0.3)	(0.8, 0.2, 0.2)	(0.4, 0.6, 0.4)	(0.7, 0.3, 0.3)	(0.9, 0.1, 0.1)	(0.4, 0.6, 0.4)	(0.7, 0.3, 0.3)	(0.9, 0.1, 0.1)
THR7	(0.9, 0.1, 0.1)	(0.8, 0.2, 0.2)	(0.4, 0.6, 0.4)	(0.4, 0.6, 0.4)	(0.6, 0.4, 0.4)	(0.9, 0.1, 0.1)	(0.9, 0.1, 0.1)	(0.8, 0.2, 0.2)	(0.1, 0.9, 0.1)	(0.2, 0.8, 0.2)	(0.6, 0.4, 0.4)	(0.3, 0.7, 0.3)	(0.2, 0.8, 0.2)	(0.6, 0.4, 0.4)	(0.3, 0.7, 0.3)	(0.9, 0.1, 0.1)	(0.4, 0.6, 0.4)	(0.7, 0.3, 0.3)	(0.3, 0.7, 0.3)	(0.1, 0.9, 0.1)
THR8	(0.4, 0.6, 0.4)	(0.7, 0.3, 0.3)	(0.9, 0.1, 0.1)	(0.4, 0.6, 0.4)	(0.7, 0.3, 0.3)	(0.9, 0.1, 0.1)	(0.8, 0.2, 0.2)	(0.6, 0.4, 0.4)	(0.9, 0.1, 0.1)	(0.9, 0.1, 0.1)	(0.8, 0.2, 0.2)	(0.1, 0.9, 0.1)	(0.2, 0.8, 0.2)	(0.6, 0.4, 0.4)	(0.3, 0.7, 0.3)	(0.7, 0.3, 0.3)	(0.9, 0.1, 0.1)	(0.4, 0.6, 0.4)	(0.7, 0.3, 0.3)	(0.1, 0.9, 0.1)
THR9	(0.9, 0.1, 0.1)	(0.8, 0.2, 0.2)	(0.9, 0.1, 0.1)	(0.4, 0.6, 0.4)	(0.3, 0.7, 0.3)	(0.4, 0.6, 0.4)	(0.8, 0.2, 0.2)	(0.6, 0.4, 0.4)	(0.9, 0.1, 0.1)	(0.9, 0.1, 0.1)	(0.8, 0.2, 0.2)	(0.1, 0.9, 0.1)	(0.2, 0.8, 0.2)	(0.6, 0.4, 0.4)	(0.3, 0.7, 0.3)	(0.1, 0.9, 0.1)	(0.2, 0.8, 0.2)	(0.6, 0.4, 0.4)	(0.3, 0.7, 0.3)	(0.8, 0.2, 0.2)
THR10	(0.6, 0.4, 0.4)	(0.9, 0.1, 0.1)	(0.9, 0.1, 0.1)	(0.8, 0.2, 0.2)	(0.1, 0.9, 0.1)	(0.2, 0.8, 0.2)	(0.6, 0.4, 0.4)	(0.3, 0.7, 0.3)	(0.3, 0.7, 0.3)	(0.8, 0.2, 0.2)	(0.6, 0.4, 0.4)	(0.8, 0.2, 0.2)	(0.9, 0.1, 0.1)	(0.4, 0.6, 0.4)	(0.3, 0.7, 0.3)	(0.4, 0.6, 0.4)	(0.4, 0.6, 0.4)	(0.3, 0.7, 0.3)	(0.8, 0.2, 0.2)	(0.9, 0.1, 0.1)
